# The association between inflammation and pubertal development: Insights from NHANES and Mendelian randomization analyses

**DOI:** 10.1097/MD.0000000000051303

**Published:** 2026-10-23

**Authors:** Daye Chen, Lei Qian, Peng Mei, Jie Qin, Ben Liu

**Affiliations:** a Department of Pediatrics, Xishan People’s Hospital of Wuxi City, Wuxi, China; b Department of Pediatrics, The Yancheng Clinical College of Xuzhou Medical University, Yancheng, China; c Department of Pediatrics, The First People’s Hospital of Yancheng, Yancheng, China; d Pediatric Intensive Care Unit, The First People’s Hospital of Yancheng, Yancheng, China.

**Keywords:** inflammation index, inflammatory proteins, Mendelian randomization analysis, NHANES, precocious puberty, pubertal status

## Abstract

Our research seeks to elucidate the interplay between inflammation and pubertal development using association and Mendelian randomization (MR) analyses, aiming to uncover the underlying potential causal relationship. Using data from the 2013 to 2016 and 2021 to 2023 National Health and Nutrition Examination Survey cycles, we assessed 3571 children and adolescents. To evaluate associations with pubertal status, logistic regression was performed for a panel of immune-inflammatory biomarkers: platelet-to-lymphocyte ratio, monocyte-to-lymphocyte ratio, neutrophil-to-lymphocyte ratio, systemic inflammation response index, systemic immune-inflammation index, and pan-immune-inflammation value. Bidirectional 2-sample MR analysis was conducted: genetic instruments for exposures were from a published genome-wide protein quantitative trait locus study (91 inflammatory proteins), and outcomes were precocious puberty (PP) summary statistics from the FinnGen consortium R11 database to infer causality. The median age of participants was 10.00 years, with 1838 males (51.47%) and 1733 females (48.53%). In association analysis, neutrophil-to-lymphocyte ratio showed a significant positive association with pubertal status across all models (fully adjusted: odds ratio [OR] = 1.198, 95% confidence interval [CI]: 1.095–1.311, *P* < .001). Systemic inflammation response index and systemic immune-inflammation index presented significant positive associations only in the fully adjusted model. Forward MR analysis suggested that genetically predicted higher levels of matrix metalloproteinase-10 (OR = 2.213, 95% CI: 1.218–4.024, *P* = .009) and osteoprotegerin (OR = 2.010, 95% CI: 1.082–3.734, *P* = .027) were associated with increased risk of PP, while higher tumor necrosis factor-β (TNF-β, OR = 0.678, 95% CI: 0.479–0.959, *P* = .028) decreased it. Reverse MR showed PP was positively associated with C-X-C motif chemokine ligand 1 (CXCL-1), fibroblast growth factor 5 (FGF-5), fibroblast growth factor 19 (FGF-19), interleukin-7, interleukin-15 receptor subunit alpha, neurturin, and negatively associated with tumor necrosis factor-related activation-induced cytokine. Inflammatory processes may contribute to PP pathogenesis via pathways influencing pubertal timing and progression, providing new insights into pubertal development mechanisms and a scientific basis for future interventions.

## 1. Introduction

Adolescence constitutes a critical period of human development, serving as a bridge between childhood and adulthood and characterized by profound transformations in the physical, psychological, and social domains. Its physiological features include gonadal maturation, the acquisition of reproductive capacity, and transformations in body morphology and function driven by rising levels of growth and sex hormones.^[1,2]^ Precocious puberty (PP) is defined by the onset of secondary sexual characteristics before age 8 in girls and 9 in boys, which is predominantly driven by the premature activation of the hypothalamic-pituitary-gonadal (HPG) axis.^[3,4]^ The adverse effects of PP are not confined to the physiological domain, which includes rapid growth velocity and compromised final height, but also encompass the social and psychological realms.^[5]^ These include emotional volatility, anxiety, depression, and social isolation, which may stem from difficulties adapting to peers and societal expectations, thereby impacting adolescents’ self-identity and mental health.^[6,7]^

The association between inflammation and puberty involves complex and diverse mechanisms. Research indicates that during normal pubertal development, pro-inflammatory cytokines can modulate hypothalamic neuronal activity, influencing the pulsatile secretion of gonadotropin-releasing hormone.^[8]^ Concurrently, tumor necrosis factor-alpha (TNF-α), secreted by adipose tissue in obese adolescents, participates in regulating pubertal onset by altering leptin sensitivity.^[9]^ Furthermore, recent studies have substantiated a direct link between inflammation and PP. At the clinical level, chronic low-grade inflammation associated with obesity can lead to premature activation of gonadotropin-releasing hormone neurons; for instance, serum C-reactive protein levels in obese children show a positive association with the incidence of PP.^[10]^ Experimental research further elucidates specific mechanisms, demonstrating that hypothalamic inflammation can accelerate the initiation of puberty via activation of the nuclear factor kappa B (NF-κB) signaling pathway.^[11]^

Immune-inflammatory biomarkers (IIBs), such as lymphocyte number (LYM), neutrophil, platelet, and monocyte (MON), are crucial for assessing disease status as they reflect the host’s immune-inflammatory balance. This has led to the widespread use of derived indices like the neutrophil-to-lymphocyte ratio (NLR), monocyte-to-lymphocyte ratio (MLR), platelet-to-lymphocyte ratio (PLR), systemic inflammation response index (SIRI), systemic immune-inflammation index (SII), and pan-immune-inflammation value (PIV) for disease risk and prognosis assessment (Fig. 1).^[12]^ Studies have demonstrated the significant clinical value of these systemic immune-inflammatory indices across various pathologies. Calculated from routine blood tests, they are cost-effective, readily accessible, and impart new prognostic significance to standard blood cell counts. Specifically, NLR, PLR, and SII have been identified as independent predictors of survival outcomes in anal cancer,^[13]^ while PLR helps identify highly aggressive subtypes of parotid gland tumors.^[14]^ Moreover, SIRI and SII reflect the inflammatory status and endothelial function in hypertensive patients,^[15]^ and NLR in multiple sclerosis patients correlates with disease activity and disability levels.^[16]^ In infectious diseases like tuberculosis, dynamic changes in these inflammatory markers provide references for monitoring disease progression.^[17]^ Overall, IIBs indicators exhibit notable clinical utility, offering simple yet effective tools for disease risk stratification, prognosis evaluation, and personalized management, particularly in settings with limited medical resources.

**Figure 1.**
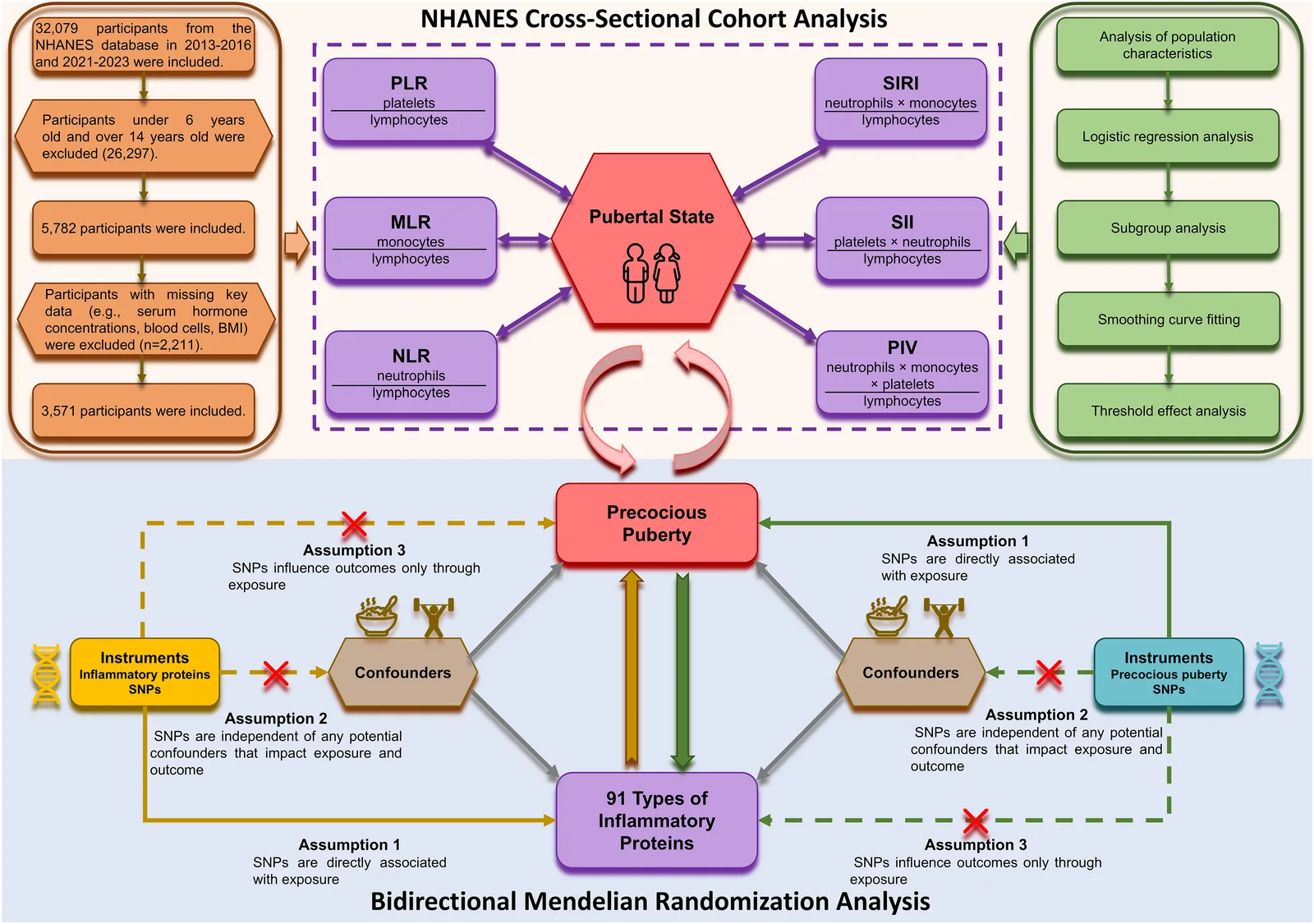
Flowchart of the study design and analysis. This flowchart depicts the overall study design, including the NHANES analysis and the bidirectional Mendelian randomization (MR) analysis. For the NHANES analysis, participants from the 2013 to 2016 and 2021 to 2023 cycles were selected. Of the 32,079 initial participants, 26,297 were excluded due to age (<6 or >14 years). After excluding 2211 participants with missing key information (e.g., hormone levels, blood cell counts, BMI, and other covariates), 3571 participants were included in the final analysis. Immune-inflammatory markers (PLR, MLR, NLR, SII, SIRI, and PIV) were calculated and analyzed in relation to hormone-defined pubertal status using logistic regression models and subgroup analyses. Furthermore, a bidirectional 2-sample MR analysis was conducted to explore potential genetic associations between inflammatory proteins and precocious puberty (PP). The MR analysis was performed based on the standard instrumental variable assumptions. BMI = body mass index, MLR = monocyte-to-lymphocyte ratio, MR = Mendelian randomization, NHANES = National Health and Nutrition Examination Survey, NLR = neutrophil-to-lymphocyte ratio, PIV = pan-immune-inflammation value, PLR = platelet-to-lymphocyte ratio, PP = precocious puberty, SII = systemic immune-inflammation index, SIRI = systemic inflammation response index, SNP = single nucleotide polymorphism.

To elucidate the causal relationship between inflammatory indices and pubertal development, this research harmonizes evidence from cross-sectional National Health and Nutrition Examination Survey (NHANES) analyses and bidirectional 2-sample Mendelian randomization (MR). The research not only reveals associations between IIBs and pubertal status but also, for the first time, identifies potential genetic associations between 91 inflammatory proteins and PP and provides exploratory insights into possible biological mechanisms. This work provides initial clues for understanding the interplay between the immune and endocrine systems during pubertal development and offers a reference for subsequent related research.

## 2. Materials and methods

### 2.1. Study design

We first investigated associations between inflammatory indices and pubertal status in NHANES, then applied bidirectional 2-sample MR to assess the causality of 91 inflammatory proteins for PP. The detailed flowchart is provided in Figure 1.

### 2.2. Study population

The NHANES database provides nationally representative public health information for the United States, offering data to address public health issues. The program has been continuously conducted for over 2 decades since 1999. NHANES data are publicly available, and researchers can access the most recent data via its official website. All data collection procedures received ethics committee approval, and all participants or their guardians provided written informed consent prior to participation.^[18]^

By searching the NHANES database, we identified survey cycles that concurrently contained data on sex hormones (TT and estradiol) and complete blood cell counts. Ultimately, data from 2013 to 2016 and 2021 to 2023, comprising 32,079 participants, were retrieved. As shown in Figure 1, we first excluded 26,297 participants outside the age range of 6 to 14 years. A total of 2211 participants were excluded owing to incomplete data regarding serum hormone concentrations, blood cell counts, physical activity (PA) questionnaires, or key covariates such as body mass index (BMI), total cholesterol (TC), 25(OH)D, and high-density lipoprotein (HDL). Following these exclusions, a total of 3571 eligible participants with complete data were included in the final analytic cohort.

### 2.3. Study variables

Assay of serum total testosterone (TT) and estradiol (*E*_2_) was performed via isotope dilution high-performance liquid chromatography-tandem mass spectrometry. Based on these hormone measurements, participants were defined as pubertal (the primary outcome) according to the following criteria: for males, TT ≥ 50 ng/dL; for females, *E*_2_ ≥ 20 pg/mL or a history of menarche.^[19]^ Complete blood count parameters were assessed on Beckman Coulter analyzers employing automated dilution and mixing, with all results expressed as ×10^9^/L. Inflammatory indices (PLR, MLR, NLR, SIRI, SII, PIV) were derived from these counts using the formulas in Figure 1 and served as the key exposure variables. To account for potential confounding, we included a comprehensive set of covariates based on clinical knowledge and literature, encompassing demographic characteristics, examination and laboratory data, hematological parameters, and lifestyle factors. Demographic characteristics comprised age (continuous, in years), sex (male/female), race/ethnicity (categorized based on NHANES definitions), country of birth (US-born or foreign-born), and poverty–income ratio (PIR, a continuous measure of socioeconomic status). Examination and laboratory data included body mass index (BMI, calculated as weight in kilograms divided by height in meters squared), TC, HDL, and 25-hydroxyvitamin D [25(OH)D]. Hematological parameters, analyzed as continuous variables, consisted of white blood cell (WBC) count, red blood cell (RBC) count, and hemoglobin (HGB). Regarding lifestyle factors, PA level was evaluated using the NHANES youth questionnaire, and participants were stratified into 3 groups by weekly activity days (>60 minutes): 0 to 2 days (inactive), 3 to 5 days (moderately active), and 6 to 7 days (active).

### 2.4. Statistical analysis in NHANES

Data were analyzed with EmpowerStats (X&Y Solutions Inc., Boston, version 6.0) and R (R Foundation, version 4.4.3). For baseline characteristics tabulated across NLR quartiles (Table 1): continuous variables were presented as median (IQR) and compared across quartiles using the Kruskal–Wallis *H* test; categorical variables were summarized as n (%) and compared using the chi-square test.

**Table 1:** Demographic and clinical characteristics of the study population according to quartiles of the neutrophil-to-lymphocyte ratio (NLR).

Variables	Total	Q1 (<0.98)	Q2 (0.99–1.29)	Q3 (1.30–1.75)	Q4 (>1.75)	*P*-value
Number	3571	888	894	895	894	
Age (yr)	10.00 (8.00–12.00)	9.00 (7.00–11.00)	10.00 (7.00–12.00)	10.00 (8.00–12.00)	11.00 (8.00–13.00)	<.001
6–8	1156 (32.37%)	370 (41.67%)	318 (35.57%)	233 (26.03%)	235 (26.29%)	
9–14	2415 (67.63%)	518 (58.33%)	576 (64.43%)	662 (73.97%)	659 (73.71%)	
Sex						.18
Male	1838 (51.47%)	467 (52.59%)	465 (52.01%)	474 (52.96%)	432 (48.32%)	
Female	1733 (48.53%)	421 (47.41%)	429 (47.99%)	421 (47.04%)	462 (51.68%)	
Country of birth						.494
Unite State	3325 (93.11%)	819 (92.23%)	833 (93.18%)	832 (92.96%)	841 (94.07%)	
Other country	246 (6.89%)	69 (7.77%)	61 (6.82%)	63 (7.04%)	53 (5.93%)	
Race/ethnicity						<.001
Mexican American	774 (21.67%)	134 (15.09%)	203 (22.71%)	196 (21.90%)	241 (26.96%)	
Non-Hispanic White	1050 (29.40%)	243 (27.36%)	264 (29.53%)	277 (30.95%)	266 (29.75%)	
Non-Hispanic Black	761 (21.31%)	283 (31.87%)	192 (21.48%)	160 (17.88%)	126 (14.09%)	
Other Hispanic	474 (13.27%)	101 (11.37%)	115 (12.86%)	121 (13.52%)	137 (15.32%)	
Other Race	512 (14.34%)	127 (14.30%)	120 (13.42%)	141 (15.75%)	124 (13.87%)	
Examination period						.01
November 1–April 30	1769 (49.54%)	402 (45.27%)	437 (48.88%)	458 (51.17%)	472 (52.80%)	
May 1–October 31	1802 (50.46%)	486 (54.73%)	457 (51.12%)	437 (48.83%)	422 (47.20%)	
Physical activity						<.001
Inactive	612 (17.14%)	132 (14.86%)	129 (14.43%)	161 (17.99%)	190 (21.25%)	
Moderately active	1199 (33.58%)	256 (28.83%)	325 (36.35%)	312 (34.86%)	306 (34.23%)	
Active	1760 (49.29%)	500 (56.31%)	440 (49.22%)	422 (47.15%)	398 (44.52%)	
Pubertal state						<.001
Pubertal	1319 (36.94%)	252 (28.38%)	306 (34.23%)	374 (41.79%)	387 (43.29%)	
Prepubertal	2252 (63.06%)	636 (71.62%)	588 (65.77%)	521 (58.21%)	507 (56.71%)	
PIR	1.54 (0.82–3.13)	1.56 (0.82–3.37)	1.65 (0.85–3.24)	1.54 (0.82–3.08)	1.46 (0.78–2.85)	.007
BMI (kg/m^2^)	19.10 (16.40–22.90)	17.50 (15.80–20.40)	18.80 (16.40–22.00)	19.70 (16.70–23.50)	20.90 (17.60–25.20)	<.001
HDL (mmol/L)	1.37 (1.19–1.63)	1.47 (1.27–1.71)	1.40 (1.19–1.63)	1.34 (1.19–1.60)	1.33 (1.14–1.55)	<.001
TC (mmol/L)	3.98 (3.54–4.47)	4.03 (3.62–4.53)	4.01 (3.57–4.47)	3.93 (3.54–4.45)	3.93 (3.52–4.45)	.04
25(OH)D (nmol/L)	60.40 (49.30–73.00)	62.15 (50.85–74.70)	61.75 (50.35–73.18)	59.40 (48.15–72.45)	59.20 (48.30–70.00)	.012
WBC (10^9^/L)	6.90 (5.70–8.30)	6.00 (5.00–7.20)	6.40 (5.50–7.70)	7.00 (6.00–8.20)	8.10 (6.90–9.50)	<.001
LYM (10^9^/L)	2.50 (2.10–3.10)	2.90 (2.40–3.60)	2.60 (2.20–3.10)	2.40 (2.10–2.90)	2.20 (1.80–2.60)	<.001
NEU# (10^9^/L)	3.40 (2.50–4.40)	2.30 (1.80–2.80)	3.00 (2.50–3.60)	3.70 (3.10–4.40)	5.00 (4.10–6.00)	<.001
MONO# (10^9^/L)	0.50 (0.40–0.70)	0.50 (0.40–0.60)	0.50 (0.40–0.60)	0.60 (0.50–0.70)	0.60 (0.50–0.70)	<.001
HGB (g/dL)	13.10 (12.50–13.80)	13.00 (12.40–13.60)	13.10 (12.50–13.80)	13.20 (12.60–13.90)	13.20 (12.60–13.80)	<.001
RBC (10^12^/L)	4.68 (4.46–4.93)	4.66 (4.45–4.87)	4.64 (4.44–4.92)	4.71 (4.47–4.96)	4.70 (4.47–4.97)	<.001
PLT (10^9^/L)	277.00 (241.00–317.00)	280.50 (243.00–319.00)	276.00 (238.00–314.00)	274.00 (241.00–315.00)	278.00 (240.00–319.00)	.517
MLR	0.21 (0.17–0.27)	0.17 (0.14–0.20)	0.19 (0.16–0.23)	0.22 (0.19–0.27)	0.28 (0.23–0.33)	<.001
PLR	108.95 (88.79–134.26)	94.67 (79.04–115.44)	102.32 (85.71–125.95)	112.08 (92.57–134.13)	128.73 (106.67–156.86)	<.001
SII	361.06 (258.08–501.64)	215.37 (176.58–256.56)	313.52 (264.85–362.18)	413.10 (357.32–478.47)	631.65 (515.51–775.25)	<.001
SIRI	0.70 (0.47–1.05)	0.38 (0.28–0.48)	0.59 (0.48–0.73)	0.82 (0.67–1.02)	1.40 (1.06–1.85)	<.001
PIV	194.52 (126.80–303.37)	107.24 (74.15–142.57)	162.48 (122.17–208.32)	224.57 (17 6.11–298.45)	390.29 (278.58–539.52)	<.001

Data are presented as median (interquartile range) for continuous variables and as number (percentage) for categorical variables. The NLR was calculated as the absolute neutrophil count divided by the absolute lymphocyte count. Participants were stratified into 4 groups based on NLR quartiles: Q1 (≤ 0.98), Q2 (0.99–1.29), Q3 (1.30–1.75), and Q4 (> 1.75).

25(OH)D = 25-hydroxyvitamin D, BMI = body mass index, HDL = high-density lipoprotein, HGB = hemoglobin, LYM = absolute lymphocyte count, MLR = monocyte-to-lymphocyte ratio, MONO# = absolute monocyte count, NEU# = absolute neutrophil count, PIV = pan-immune inflammation value (neutrophil × monocyte × platelet/lymphocyte), PIR = poverty- income ratio, PLR = platelet-to-lymphocyte ratio, PLT = platelet, RBC = red blood cell, SII = systemic immune-inflammation index (platelet × neutrophil/lymphocyte), SIRI = systemic inflammation response index (neutrophil × monocyte/lymphocyte), TC = total cholesterol, WBC = white blood cell.

For the primary analysis of pubertal status (binary outcome: pubertal vs prepubertal), we applied multivariable logistic regression. Inflammatory indices were entered either as continuous variables or as categorical quartile variables. To evaluate the association between these indices and pubertal status, we constructed 3 sequential logistic regression models with increasing levels of covariate adjustment. Model 1 was unadjusted. Model 2 was adjusted for demographic factors, including age, sex, race/ethnicity, country of birth, examination period, and PIR. Model 3 was fully adjusted, incorporating all variables from model 2 plus PA level, BMI, HDL, TC, WBC, HGB, RBC, and 25(OH)D.

To test robustness, subgroup analyses were performed and visualized with forest plots. Finally, sensitivity analyses were conducted by rerunning the logistic regression models after excluding outliers (BMI > 35 kg/m^-2^- or WBC < 4 × 10^-9^-/L or > 20 × 10^-9^-/L).

### 2.5. Data sources for MR analysis

For inflammatory proteins, we utilized summary-level protein quantitative trait locus data from a study of 14,824 Europeans by Zhao et al.^[20]^ For PP, genome-wide association study summary statistics were derived from the FinnGen consortium R11 (202 cases, 434,894 controls).^[21]^ Ethical approval was inherent in the original studies, thereby waiving the need for further ethical review of this analysis.

### 2.6. Instrumental variable selection

As the number of SNPs available under the *P* < 5 × 10^−8^ threshold was inadequate for robust MR analysis, we adopted a more lenient threshold (*P* < 5 × 10^−6^) to select SNPs significantly associated with the exposure as potential instrumental variables. Clumping was performed to ensure independence (*r*^2^ < 0.001, distance = 10,000 kb), and SNPs with a minor allele frequency ≤ 0.01 were excluded. Instrument strength was quantified by the *F*-statistic^[22]^; variants with *F* < 10 were considered weak and removed. To minimize pleiotropy, we manually queried the IEU genome-wide association study database for associations between selected SNPs and potential confounders (e.g., BMI, alcohol consumption, smoking, sex hormones, type 2 diabetes), excluding any with significant associations.

### 2.7. MR analysis

We first conducted forward MR analyses to explore potential causal effects of the 91 inflammatory proteins on PP, adhering to the standard MR assumptions (Fig. 1). Reverse MR analyses were then performed to examine causality in the opposite direction. The inverse-variance weighted (IVW) method served as the primary analytical approach. To evaluate robustness and potential pleiotropy, we supplemented IVW with several sensitivity methods: weighted median, weighted mode, MR-Egger regression, and simple mode.

We assessed heterogeneity by applying Cochran’s *Q* statistic from the IVW and MR-Egger methods, considering *P* > .05 as evidence of its absence. Horizontal pleiotropy was evaluated via the MR-Egger intercept, where a value close to 0 indicates negligible pleiotropy. Furthermore, the Mendelian Randomization Pleiotropy RESidual Sum and Outlier test was implemented to detect global pleiotropy, identify outlier SNPs, and provide corrected estimates after their removal. A Bonferroni correction was applied to IVW-derived P-values, setting the significance threshold at 5.49 × 10^−4^ (0.05/91 tests). *P*-values below this threshold were considered strong evidence of causality, whereas those between .05 and the threshold were deemed to be potentially significant.

## 3. Results

### 3.1. Baseline characteristics

The analysis included 3571 children and adolescents, who were stratified into 4 groups by NLR quartiles for subsequent analysis (Q1: <0.98; Q2: 0.99–1.29; Q3: 1.30–1.75; Q4: >1.75; Table 1). The sex distribution was balanced (51.47% male, 48.53% female), and 93.11% of participants were born in the United States. Comparative analyses across NLR quartiles revealed statistically significant differences in several variables. As NLR levels increased, the study population showed a trend towards older age (median age increased from 9 to 11 years), and the proportion of individuals classified as having reached pubertal state significantly increased (from 28.38 to 43.29%). Concurrently, median BMI increased with higher NLR quartiles (from 17.50 kg/m^2^ to 20.90 kg/m^2^), whereas levels of HDL and 25(OH)D decreased. Regarding PA, the proportion of “Active” individuals decreased (from 56.31 to 44.52%), while the proportion of “Inactive” individuals increased (from 14.86 to 21.25%) in higher NLR quartiles. Consistent with the increase in NLR quartiles, other inflammatory indices also demonstrated progressive changes. The median values of MLR, PLR, SII, SIRI, and PIV increased across increasing NLR quartiles (Q1–Q4), as shown in Table 1, with all comparisons reaching statistical significance (all *P* < .001). However, no significant differences were observed across NLR quartiles for sex, country of birth, or platelet.

### 3.2. Association between inflammatory indices and pubertal status

The associations between various blood cell-based inflammatory indices and pubertal status were investigated using multivariable logistic regression models across different adjustment levels, as presented in Table 2. In the unadjusted Model 1, PLR (odds ratio [OR] = 1.002, 95% confidence interval [CI]: 1.001–1.004, *P* = .004), MLR (OR = 2.732, 95% CI: 1.376–5.425, *P* = .004), NLR (OR = 1.122, 95% CI: 1.036–1.214, *P* = .004), and PIV (OR = 1.000, 95% CI: 0.999–1.000, *P* = .043) showed statistically significant associations. After adjusting for demographic and socioeconomic factors (age, sex, country of birth, race/ethnicity, PIR, survey cycle) in model 2, NLR (*P* = .049) and PIV (*P* = .013) remained significant, while the significance for PLR and MLR was attenuated. In the fully adjusted model 3, which additionally included PA, BMI, HDL, TC, WBC, HGB, RBC, and 25(OH)D levels, NLR (OR = 1.198, 95% CI: 1.095–1.311, *P* < .001), SIRI (OR = 1.250, 95% CI: 1.033–1.512, *P* = .022), and SII (OR = 1.000, 95% CI: 1.000–1.001, *P* = .025) exhibited significant positive associations. Overall, NLR demonstrated a robust and consistent association across all models. In contrast, SIRI and SII only showed significant associations after comprehensive adjustment for potential confounders.

**Table 2:** Logistic regression analysis of immune-inflammatory biomarkers and pubertal status.

Immune-inflammatory biomarkers	Model 1		Model 2		Model 3	
	OR (95% CI)	*P*-value	OR (95% CI)	*P*-value	OR (95% CI)	*P*-value
PLR	1.002 (1.001, 1.004)	.004	1.002 (1.000, 1.004)	.051	0.999 (0.997, 1.002)	.667
MLR	2.732 (1.376, 5.425)	.004	1.822 (0.712, 4.659)	.211	1.466 (0.485, 4.428)	.498
NLR	1.122 (1.036, 1.214)	.004	1.100 (1.001, 1.210)	.049	1.198 (1.095, 1.311)	<.001
SIRI	0.997 (0.913, 1.088)	.943	0.929 (0.813, 1.062)	.280	1.250 (1.033, 1.512)	.022
SII	1.000 (1.000, 1.000)	.767	1.000 (1.000, 1.000)	.532	1.000 (1.000, 1.001)	.025
PIV	1.000 (0.999, 1.000)	.043	0.999 (0.999, 1.000)	.013	1.000 (1.000, 1.001)	.193

Model 1: no covariates were adjusted; Model 2: adjusted for age, sex, race/ethnicity, country of birth, examination period, PIR; Model 3: adjusted for age, sex, race/ethnicity, country of birth, examination period, PIR, physical activity, BMI, HDL, TC, WBC, HGB, RBC, and 25(OH)D.

25(OH)D = 25-hydroxyvitamin D, BMI = body mass index, CI = confidence interval, HDL = high-density lipoprotein, HGB = hemoglobin, MLR = monocyte-to-lymphocyte ratio, NLR = neutrophil-to-lymphocyte ratio, OR = odds ratio, PIR = poverty-income ratio, PIV = pan-immune inflammation value (neutrophil × monocyte × platelet/lymphocyte), PLR = platelet-to-lymphocyte ratio, RBC = red blood cell, SII = systemic immune-inflammation index (platelet × neutrophil/lymphocyte), SIRI = systemic inflammation response index (neutrophil × monocyte/lymphocyte), TC = total cholesterol, WBC = white blood cell.

For inflammatory indices significant in Model 3, we performed quartile-based analyses. A significant dose-response relationship was observed for NLR (Supplement 1, Supplemental Digital Content 1), with the highest quartile (Q4) associated with increased odds of puberty versus Q1 (OR = 1.670, 95% CI: 1.224–2.279, *P* = .001). Mirroring its continuous results, SIRI also showed elevated odds in Q3 (OR = 1.408, 95% CI: 1.044–1.901, *P* = .025) and Q4 (OR = 1.445, 95% CI: 1.018–2.052, *P* = .040), albeit without a significant trend. In contrast, the SII demonstrated a less stable association; while significant as a continuous variable, neither its quartile groups nor the trend test reached significance. The positive association between NLR and pubertal status was consistently observed across adolescents of different sexes, ages, levels of PA, and BMI groups (Supplement 2, Supplemental Digital Content 2). No significant effect modification was identified (all *P*-for-interaction > .05).

### 3.3. Sensitivity analysis

Sensitivity analyses excluding participants with BMI > 35 or out-of-range WBC counts were performed to evaluate the robustness of the findings. As shown in Supplement 3, Supplemental Digital Content 3, the positive association between NLR and pubertal status persisted in model 3 (OR = 1.188, 95% CI: 1.084–1.302, *P* < .001). The stability of the effect estimate reinforces the validity of NLR as an associated inflammatory marker. The SIRI (OR = 1.236, 95% CI: 1.017–1.504, *P* = .034) and SII (OR = 1.000, 95% CI: 1.000–1.001, *P* = .034) also continued to show significant positive associations, although their *P*-values showed a slight increase compared to the primary analysis. In contrast, neither the PLR nor the MLR reached significance in model 3 within this sensitivity analysis.

### 3.4. Mendelian randomization analysis

To address the limitations of observational studies, a bidirectional 2-sample MR approach was employed to systematically explore potential causal effects of 91 inflammatory proteins on PP. Detailed information on the instrumental variables and full results are available in Supplement 4, Supplemental Digital Content 4 and Supplement 5, Supplemental Digital Content 5.

In the forward MR analysis, the primary IVW method identified significant potential causal associations for 3 inflammatory proteins (Fig. 2). Specifically, matrix metalloproteinase-10 (MMP-10; OR = 2.213, 95% CI: 1.218–4.024, *P* = .009) and osteoprotegerin (OPG; OR = 2.010, 95% CI: 1.082–3.734, *P* = .027) were linked to a higher risk of PP, while TNF-β (OR = 0.678, 95% CI: 0.479–0.959, *P* = .028) showed a negative association with the risk of PP.

**Figure 2.**
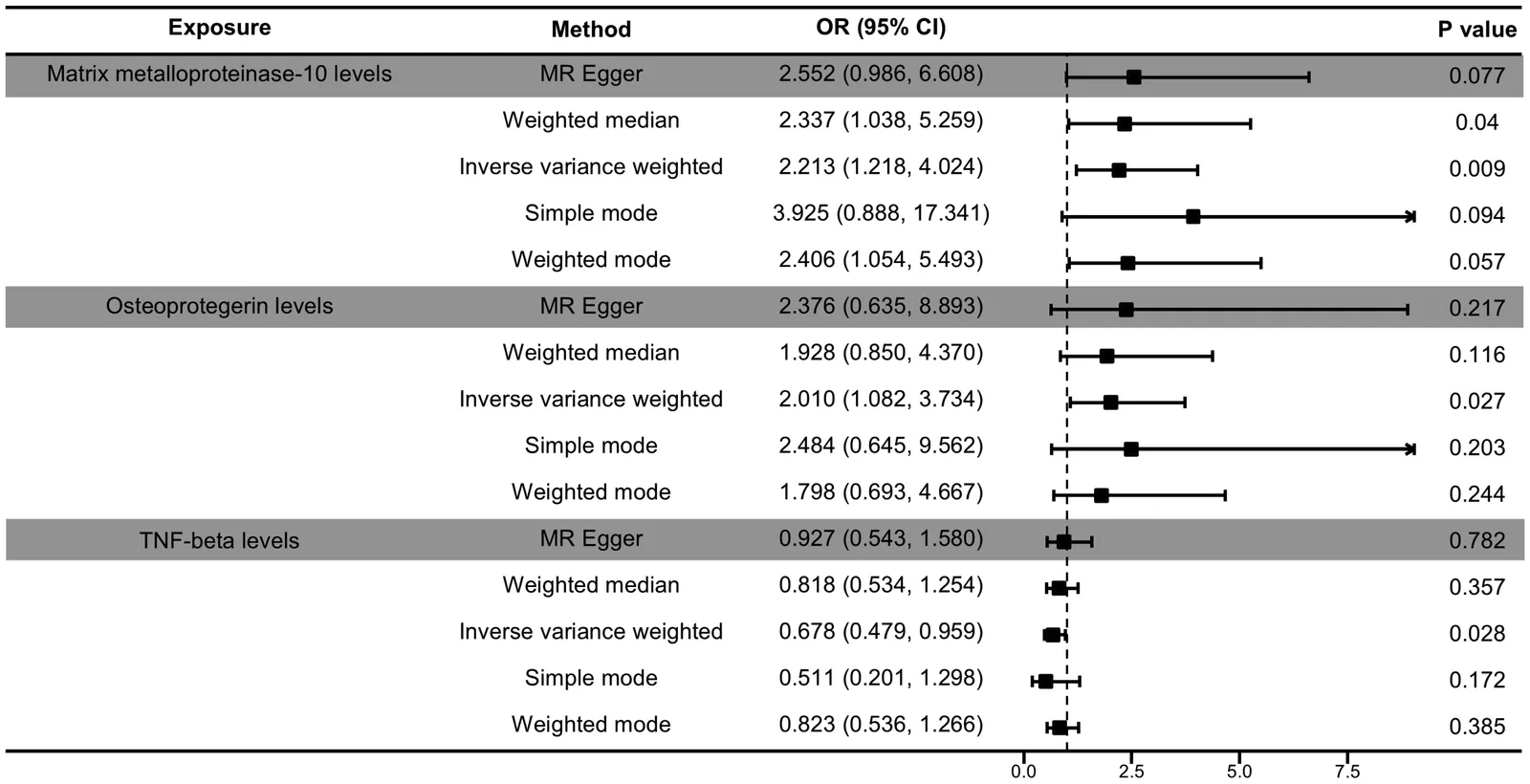
Forest plot of the causal effects of inflammatory proteins on precocious puberty. CI = confidence interval, MR = Mendelian randomization, OR = odds ratio, TNF = tumor necrosis factor.

Reverse MR analysis identified PP as being causally linked to altered levels of 7 inflammatory proteins (Fig. 3). Specifically, PP showed a positive association with levels of C-X-C motif chemokine ligand 1 (β = 0.032, 95% CI: 0.002–0.062, *P* = .037), FGF-19 (β = 0.037, 95% CI: 0.004–0.070, *P* = .030), FGF-5 (β = 0.044, 95% CI: 0.011–0.076, *P* = .009), IL-15RA (β = 0.045, 95% CI: 0.007–0.082, *P* = .019), interleukin-7 (IL-7; β = 0.034, 95% CI: 0.004–0.064, *P* = .025), and neurturin (β = 0.045, 95% CI: 0.010–0.080, *P* = .012). A negative association was observed between PP and tumor necrosis factor-related activation-induced cytokine (β = −0.045, 95% CI: −0.075–-0.015, *P* = .004).

**Figure 3.**
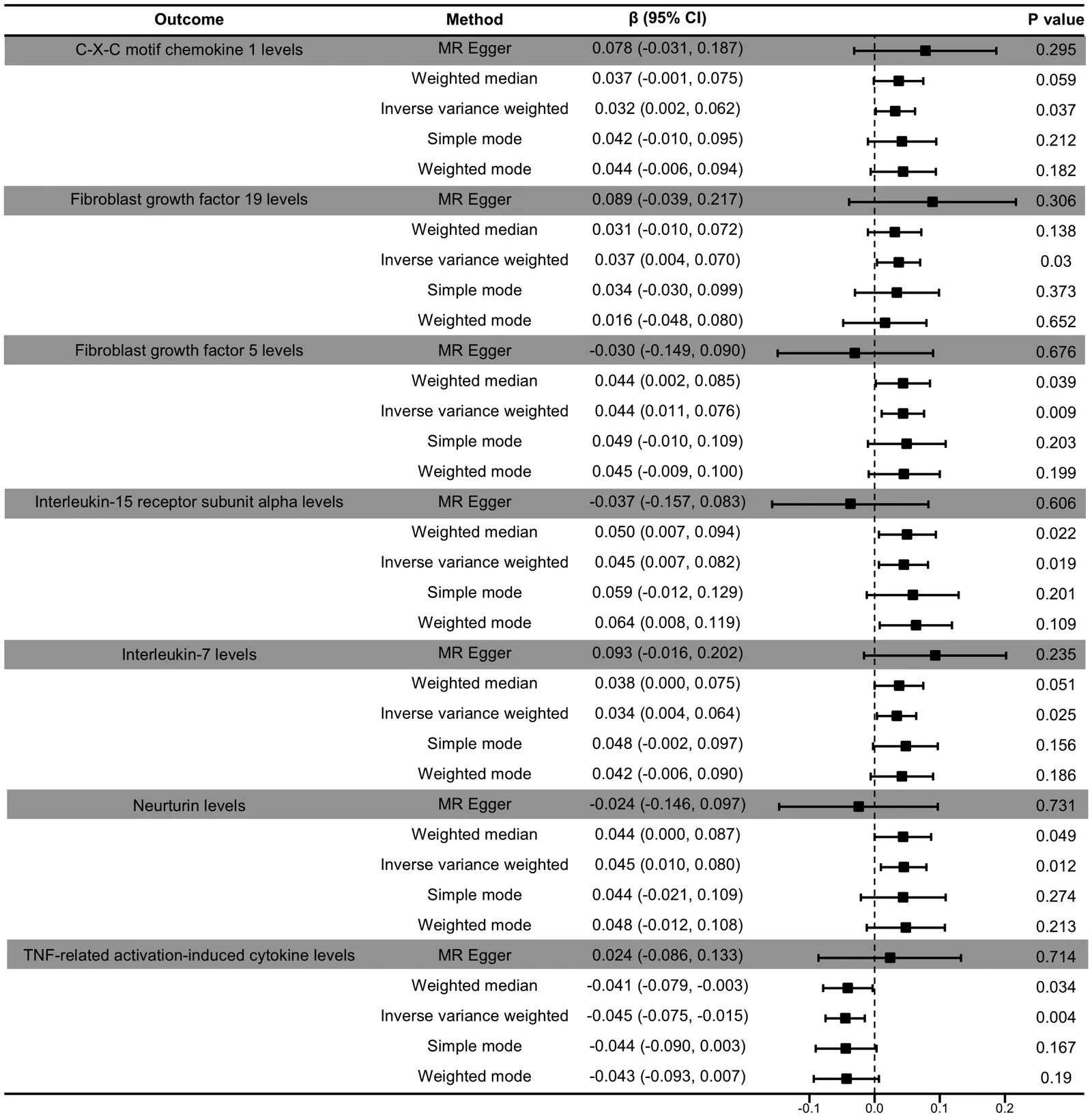
Forest plot of the causal effects of precocious puberty on inflammatory protein levels. β: Regression coefficient; CI = confidence interval, MR = Mendelian randomization, TNF = tumor necrosis factor.

Sensitivity analyses supported the robustness of all significant associations. We found no evidence of heterogeneity (Cochran’s *Q*, all *P* > .05) or horizontal pleiotropy (MR-Egger intercept and Mendelian Randomization Pleiotropy RESidual Sum and Outlier global tests, all *P* > .05; Table 3). Moreover, a Bonferroni correction was applied to the *P*-values from the IVW method, with all core results showing *P*-values ranging from 5.49 × 10^−4^ to .05.

**Table 3:** Sensitivity analysis of Mendelian randomization.

Exposure	Outcome	Heterogeneity				Pleiotropy		
		IVW		MR-Egger		MR-Egger		MR-PRESSO
		*Q*	*P*	*Q*	*P*	Intercept	*P*	*P*
Matrix metalloproteinase-10 levels	Precocious puberty	10.506	.652	10.363	.584	−0.027	.713	.701
Osteoprotegerin levels	Precocious puberty	7.097	.982	7.018	.973	−0.023	.782	.982
TNF-beta levels	Precocious puberty	22.916	0.406	20.699	.477	−0.091	.151	.368
Precocious puberty	C-X-C motif chemokine 1 levels	1.791	.617	1.044	.593	−0.026	.479	.616
Precocious puberty	Fibroblast growth factor 19 levels	3.649	.302	2.713	.258	−0.030	.493	.380
Precocious puberty	Fibroblast growth factor 5 levels	1.948	.583	0.396	.821	0.042	.339	.629
Precocious puberty	Interleukin-15 receptor subunit alpha levels	3.899	.273	1.964	.375	0.046	.299	.402
Precocious puberty	Interleukin-7 levels	1.309	.727	0.089	.957	−0.034	.384	.770
Precocious puberty	Neurturin levels	3.278	.351	1.915	.384	0.040	.363	.448
Precocious puberty	TNF-related activation-induced cytokine levels	1.676	.642	0.059	.971	−0.039	.331	.719

This table presents sensitivity analyses to assess the robustness of the causal estimates from the bidirectional MR analysis. The heterogeneity among individual SNP estimates was evaluated using Cochran’s *Q* statistic in both the IVW and MR-Egger methods (all *P* > .05, indicating no significant heterogeneity). Horizontal pleiotropy was assessed using the MR-Egger intercept test (all *P* > .05) and the MR-PRESSO global test (all *P* > .05), suggesting no evidence of directional pleiotropy. The instrumental variables for inflammatory proteins were derived from a large-scale genome-wide association study (GWAS) of 14,824 European individuals, and summary statistics for precocious puberty were obtained from the FinnGen consortium (R11 release), comprising 202 cases and 4,34,894 controls of European ancestry.

GWAS = genome-wide association study, IVW = Inverse-variance weighted, MR = Mendelian randomization, MR-PRESSO = Mendelian Randomization Pleiotropy RESidual Sum and Outlier, *P* = *P*-value, Q = statistics Q, SNP = single nucleotide polymorphism, TNF = tumour necrosis factor.

## 4. Discussion

Combining cross-sectional NHANES data and bidirectional MR analysis, this study investigated the inflammation-puberty link and its causal nature. Observational results showed that NLR, SII, and SIRI were positively associated with pubertal status, with NLR emerging as the most robust marker, exhibiting a pronounced dose-response relationship. The MR analysis further identified that elevated levels of MMP-10 and OPG might increase the risk of PP, whereas TNF-β appeared to exert a protective effect. The reverse MR analysis suggested that PP itself could influence the circulating levels of several inflammatory proteins, providing suggestive exploratory evidence for a potential bidirectional association between inflammation and puberty.

In the observational analysis, we found that NLR, SII, and SIRI, which are calculated from routine blood counts, were significantly associated with pubertal status. It should be clarified that these indicators are surrogate markers of systemic inflammation reflecting the balance between innate and adaptive immunity, rather than direct inflammatory mediators such as interleukin-6 (IL-6) or TNF-α. As the most robust marker among them, an elevated NLR typically indicates a relative increase in neutrophils (innate immunity) and a relative decrease in lymphocytes (adaptive immunity), representing a state of relative pro-inflammatory predominance. This finding aligns with previous research and supports the role of chronic low-grade inflammation in neuroendocrine regulation.^[23]^ For instance, existing studies have shown that pro-inflammatory cytokines such as IL-6 and TNF-α can directly promote pubertal onset,^[24]^ while resistin has been found not only to act on multiple levels of the HPG axis but also to amplify the synthesis of IL-6 and TNF-α through a positive feedback mechanism, thereby accelerating pubertal progression.^[25,26]^ Another cross-sectional study observed elevated WBC and neutrophil counts in girls during puberty, further confirming the concomitant relationship between inflammatory burden and pubertal progression.^[27]^ Building upon this evidence, our study further clarifies the critical role of the neutrophil-lymphocyte balance in this process through a comprehensive comparison of NLR, SII, and SIRI.

However, the cross-sectional design precludes determining the directionality of the association between inflammatory indices and pubertal status; it remains unclear whether elevated NLR drives pubertal onset or is a secondary consequence of endocrine changes during puberty. To investigate causality, we leveraged an MR analysis to evaluate the relationship between inflammatory proteins and PP. MMP-10 was implicated as a potential risk factor for PP in our study. MMP-10 has been demonstrated to directly promote inflammation by activating the NF-κB signaling pathway. In a model of peritoneal inflammation, the absence of MMP-10 significantly attenuated inflammation and fibrosis, a mechanism linked to the inhibition of NF-κB subunit p65 phosphorylation and the expression of downstream inflammatory mediators.^[28]^ In the context of cardiovascular disease, MMP-10 levels correlate positively with inflammatory factors like TNF-α and can amplify the inflammatory process by inducing the expression of pro-inflammatory cytokines such as IL-1β via the Akt phosphorylation pathway.^[29]^ Given that pro-inflammatory cytokines (e.g., TNF-α, IL-1β) have been reported to promote HPG axis activation, MMP-10 might contribute to creating a pro-inflammatory microenvironment conducive to earlier puberty through these mechanisms.^[24]^ Similarly, OPG showed a positive association with PP risk in our analysis. Although OPG exerts anti-inflammatory effects in models like rheumatoid arthritis by inhibiting RANKL, its biological function is context-dependent.^[30]^ In an asthma model, OPG was identified as a key factor inducing Th2-polarized allergic inflammation, and its deficiency significantly suppressed pulmonary inflammatory cell infiltration, the expression of Th2 cytokines, and mucus hypersecretion.^[31]^ Moreover, in patients with chronic inflammation, plasma OPG levels correlate positively with pro-inflammatory mediators like IL-6 and hsCRP.^[32]^ These findings suggest that, in specific contexts, OPG may play a pro-inflammatory role. We therefore hypothesize that OPG might increase susceptibility to PP by promoting a Th2-biased inflammatory state, indirectly affecting HPG axis function or the local immune environment in gonadal tissues. On the other hand, the protective effect observed for TNF-β further underscores the complexity and potential antagonistic roles of different inflammatory pathways in pubertal regulation. This context-dependent dual functionality is echoed for related molecules: for instance, the homologous molecule LIGHT promotes synovial inflammation in rheumatoid arthritis via the LTβR pathway,^[33]^ whereas membrane-bound TNF-β expressed on T cells can suppress excessive intestinal inflammation in an acute colitis model via LTβR signaling, the absence of which exacerbates inflammation.^[34]^ In light of this, our findings suggest that, within the specific context of pubertal initiation, TNF-β might exert its protective effect against PP primarily by activating similar immunoregulatory circuits or antagonizing other pro-inflammatory signals.

The reverse MR analysis indicated that a genetic predisposition to PP leads to elevated levels of several circulating inflammatory proteins. The underlying mechanism likely involves multi-system collaboration: the increase in estrogen and testosterone levels, driven by the premature activation of the HPG axis, can regulate immune cell function and may potentially promote the release of inflammatory mediators such as C-X-C motif chemokine ligand 1, IL-7, and interleukin-15 receptor subunit alpha (IL-15Rα).^[35,36]^ Among these, the elevated levels of IL-7 and IL-15Rα may suggest a concurrent immune system maturation process during puberty. IL-7 is considered essential for lymphocyte development, while IL-15Rα is thought to play a critical role in maintaining NK cells and memory CD8+ T cells.^[37,38]^ Concurrently, tissue growth and remodeling processes accompanying puberty might explain the increase in growth factors like fibroblast growth factor 5 and fibroblast growth factor 19, which can subsequently activate neutrophil chemotaxis and related inflammatory responses.^[39,40]^ Furthermore, sex hormones may reprogram adipose tissue metabolism, triggering the release of pro-inflammatory adipokines and thereby fueling a state of systemic inflammation.^[41,42]^ Our observations also indicated that PP leads to decreased tumor necrosis factor-related activation-induced cytokine levels. This is interpreted as a protective adaptation to suppress bone resorption through restrained osteoclast activity. During the pubertal growth spurt, a period demanding massive new bone formation, this adaptation shifts the balance toward osteoblast-dominated accumulation, providing a mechanistic explanation for the advanced bone age seen in children with PP.^[43]^

This study systematically explored the complex relationship between inflammatory markers and pubertal status. Its primary strength lies in integrating evidence from different dimensions: first, revealing observational associations using large-scale cross-sectional data, then employing bidirectional MR analysis to infer causal directions from a genetic perspective, and, for the first time, suggesting a potential bidirectional regulatory relationship, providing new insights into the underlying biological mechanisms. However, several limitations warrant consideration. First, despite adjusting for known confounders in both the observational and MR analyses, the possibility of residual bias from unmeasured confounding factors cannot be entirely ruled out. Second, the data primarily originate from European and American populations; thus, the generalizability of our findings to other ethnicities and populations requires further validation. Furthermore, in the MR analysis, we applied the Bonferroni correction with a significance threshold set at *P* < 5.49 × 10^−4^. All reported associations had P-values falling between this threshold and 0.05. While these do not meet the stringent standard for strong genetic evidence, they nonetheless suggest potential associations between the exposures and outcomes, providing valuable clues for future research. Finally, our MR analysis pinpoints potential roles for specific inflammatory proteins in PP, shedding further light on the complex interplay between inflammatory pathways and puberty. Future studies should validate the specific mechanistic roles of these proteins in pubertal regulation through animal experiments or prospective clinical cohorts. Such work is crucial to ascertain their translational value as potential targets for preventing or treating PP.

## 5. Conclusion

By integrating cross-sectional observational analysis with genetic evidence exploration, this study systematically elucidates a significant association between peripheral blood inflammatory indices and pubertal development. Our observational findings indicate that IIBs, particularly the NLR, are independently associated with pubertal status. Further MR analysis provided suggestive evidence that genetically predicted levels of MMP-10 and OPG were associated with an increased risk of PP, while genetically predicted TNF-β levels showed a negative association with PP risk. The reverse MR analysis provides suggestive exploratory evidence that PP may reciprocally modulate immune activity by altering inflammatory mediator levels, pointing toward a potential bidirectional pattern between inflammatory pathways and pubertal timing. These findings enhance our pathophysiological understanding of pubertal onset and provide a foundation for functional studies aimed at elucidating the specific molecular pathways involved. Furthermore, they highlight the potential of inflammatory markers as predictive biomarkers or therapeutic targets for PP.

## Acknowledgments

We thank those who participated in and supported this study.

## Author contributions

**Conceptualization:** Jie Qin, Ben Liu.

**Data curation:** Lei Qian, Peng Mei.

**Formal analysis:** Daye Chen, Lei Qian, Ben Liu.

**Funding acquisition:** Jie Qin, Ben Liu.

**Investigation:** Daye Chen, Jie Qin.

**Methodology:** Daye Chen.

**Software:** Lei Qian, Peng Mei.

**Supervision:** Jie Qin, Ben Liu.

**Visualization:** Peng Mei, Ben Liu.

**Writing – original draft:** Daye Chen, Lei Qian, Peng Mei, Jie Qin, Ben Liu.

**Writing – review & editing:** Jie Qin, Ben Liu.










