# Supplementary material for: The association between inflammation and pubertal development: Insights from NHANES and Mendelian randomization analyses
Source: Medicine (Baltimore). 2026 Oct 23;105(43):e51303. doi: 10.1097/MD.0000000000051303 (PMC13706194; doi:10.1097/MD.0000000000051303)
Supplement: Supplementary file 1 [file medi-105-e51303-s001.docx]

**Supplement 1.** Logistic regression analysis of NLR, SIRI, SII and pubertal status.

| **Model** | **Model 1**  **OR (95% CI) P-value** | **Model 2**  **OR (95% CI) P-value** | **Model 3**  **OR (95% CI) P-value** |
| --- | --- | --- | --- |
| NLR | 1.122 (1.036, 1.214) 0.004 | 1.100 (1.001, 1.210) 0.049 | 1.198 (1.095, 1.311) <0.001 |
| Q1 | Reference | Reference | Reference |
| Q2 | 1.313 (1.074, 1.606) 0.008 | 1.278 (0.995, 1.640) 0.055 | 1.264 (0.948, 1.685) 0.111 |
| Q3 | 1.812 (1.487, 2.207) <0.001 | 1.402 (1.097, 1.791) 0.007 | 1.462 (1.096, 1.949) 0.010 |
| Q4 | 1.926 (1.582, 2.346) <0.001 | 1.462 (1.140, 1.875) 0.003 | 1.670 (1.224, 2.279) 0.001 |
| P for trend | <0.001 | 0.006 | 0.001 |
| SIRI | 0.997 (0.913, 1.088) 0.94328 | 0.929 (0.813, 1.062) 0.27978 | 1.250 (1.033, 1.512) 0.02166 |
| Q1 | Reference | Reference | Reference |
| Q2 | 1.104 (0.908, 1.342) 0.322 | 0.973 (0.764, 1.241) 0.828 | 1.223 (0.921, 1.624) 0.163 |
| Q3 | 1.316 (1.086, 1.595) 0.005 | 1.182 (0.927, 1.508) 0.178 | 1.408 (1.044, 1.901) 0.025 |
| Q4 | 1.156 (0.952, 1.403) 0.143 | 0.892 (0.699, 1.138) 0.358 | 1.445 (1.018, 2.052) 0.040 |
| P for trend | 0.161 | 0.356 | 0.066 |
| SII | 1.000 (1.000, 1.000) 0.767 | 1.000 (1.000, 1.000) 0.532 | 1.000 (1.000, 1.001) 0.025 |
| Q1 | Reference | Reference | Reference |
| Q2 | 1.022 (0.841, 1.241) 0.829 | 1.013 (0.795, 1.289) 0.918 | 1.108 (0.839, 1.464) 0.471 |
| Q3 | 1.145 (0.944, 1.388) 0.169 | 1.029 (0.808, 1.312) 0.815 | 1.148 (0.859, 1.535) 0.350 |
| Q4 | 1.206 (0.995, 1.462) 0.056 | 0.954 (0.749, 1.215) 0.704 | 1.263 (0.924, 1.725) 0.143 |
| P for trend | 0.032 | 0.655 | 0.156 |

Model 1: no covariates were adjusted; Model 2: adjusted for age, sex, race/ethnicity, country of birth, examination period, PIR; Model 3: adjusted for age, sex, race/ethnicity, country of birth, examination period, PIR, physical activity, BMI, HDL, TC, WBC, HGB, RBC, and 25(OH)D. 95% CI: 95% confidence interval; OR, odds ratio; NLR, neutrophil-to-lymphocyte ratio; SIRI, systemic inflammation response index (neutrophil × monocyte / lymphocyte); SII, systemic immune-inflammation index (platelet × neutrophil / lymphocyte); PIR, income-to-poverty ratio; BMI, body mass index; HDL, high-density lipoprotein; TC, total cholesterol; 25(OH)D, 25-hydroxyvitamin D; WBC, white blood cell count; HGB, hemoglobin; RBC, red blood cell count.
