# Supplementary material for: The association between inflammation and pubertal development: Insights from NHANES and Mendelian randomization analyses
Source: Medicine (Baltimore). 2026 Oct 23;105(43):e51303. doi: 10.1097/MD.0000000000051303 (PMC13706194; doi:10.1097/MD.0000000000051303)
Supplement: Supplementary file 2 [file medi-105-e51303-s002.docx]

**Supplement 2.** Subgroup analysis of the association between NLR and pubertal status.

Model 1: no covariates were adjusted; Model 2: adjusted for age, sex, race/ethnicity, country of birth, examination period, PIR; Model 3: adjusted for age, sex, race/ethnicity, country of birth, examination period, PIR, physical activity, BMI, HDL, TC, WBC, HGB, RBC, and 25(OH)D. 95% CI: 95% confidence interval; OR, odds ratio; NLR, neutrophil-to-lymphocyte ratio; PIR, income-to-poverty ratio; BMI, body mass index; HDL, high-density lipoprotein; TC, total cholesterol; 25(OH)D, 25-hydroxyvitamin D; WBC, white blood cell count; HGB, hemoglobin; RBC, red blood cell count.
