# Supplementary material for: The association between inflammation and pubertal development: Insights from NHANES and Mendelian randomization analyses
Source: Medicine (Baltimore). 2026 Oct 23;105(43):e51303. doi: 10.1097/MD.0000000000051303 (PMC13706194; doi:10.1097/MD.0000000000051303)
Supplement: Supplementary file 3 [file medi-105-e51303-s003.docx]

**Supplement 3.** Sensitivity analysis of immune-inflammatory biomarkers and pubertal status.

| **Model** | **Model 1**  **OR (95% CI) P-value** | **Model 2**  **OR (95% CI) P-value** | **Model 3**  **OR (95% CI) P-value** |
| --- | --- | --- | --- |
| PLR | 1.002 (1.001, 1.004) 0.007 | 1.002 (1.000, 1.004) 0.039 | 1.000 (0.997, 1.003) 0.903 |
| MLR | 2.737 (1.344, 5.572) 0.006 | 1.722 (0.655, 4.532) 0.271 | 1.268 (0.406, 3.965) 0.683 |
| NLR | 1.144 (1.051, 1.246) 0.002 | 1.105 (1.005, 1.215) 0.038 | 1.188 (1.084, 1.302) <0.001 |
| SIRI | 1.018 (0.922, 1.124) 0.722 | 0.933 (0.814, 1.071) 0.324 | 1.236 (1.017, 1.504) 0.034 |
| SII | 1.000 (1.000, 1.000) 0.588 | 1.000 (1.000, 1.000) 0.666 | 1.000 (1.000, 1.001) 0.034 |
| PIV | 1.000 (0.999, 1.000) 0.072 | 0.999 (0.999, 1.000) 0.019 | 1.000 (1.000, 1.001) 0.211 |

Model 1: no covariates were adjusted; Model 2: adjusted for age, sex, race/ethnicity, country of birth, examination period, PIR; Model 3: adjusted for age, sex, race/ethnicity, country of birth, examination period, PIR, physical activity, BMI, HDL, TC, WBC, HGB, RBC, and 25(OH)D. 95% CI: 95% confidence interval; OR, odds ratio; PLR, platelet-to-lymphocyte ratio; MLR, monocyte-to-lymphocyte ratio; NLR, neutrophil-to-lymphocyte ratio; SIRI, systemic inflammation response index (neutrophil × monocyte / lymphocyte); SII, systemic immune-inflammation index (platelet × neutrophil / lymphocyte); PIV, pan-immune inflammation value (neutrophil × monocyte × platelet / lymphocyte); PIR, income-to-poverty ratio; BMI, body mass index; HDL, high-density lipoprotein; TC, total cholesterol; 25(OH)D, 25-hydroxyvitamin D; WBC, white blood cell count; HGB, hemoglobin; RBC, red blood cell count.
