# Supplementary material for: The association between inflammation and pubertal development: Insights from NHANES and Mendelian randomization analyses
Source: Medicine (Baltimore). 2026 Oct 23;105(43):e51303. doi: 10.1097/MD.0000000000051303 (PMC13706194; doi:10.1097/MD.0000000000051303)
Supplement: Supplementary file 4 [file medi-105-e51303-s004.docx]

**Supplement 4.** Summary of SNPs included in the studies.

| **Exposure** | **SNP** | **CHR** | **EA** | **OA** | **EAF** | **β** | **SE** | **P-value** | **F-value** |
| --- | --- | --- | --- | --- | --- | --- | --- | --- | --- |
| **Matrix metalloproteinase-10 levels** | rs112062276 | 6 | T | G | 0.06 | 0.12 | 0.02 | 1.4E-06 | 23.34 |
|  | rs11225354 | 11 | T | C | 0.88 | 0.30 | 0.02 | 5.4E-60 | 266.90 |
|  | rs112327130 | 8 | C | G | 0.02 | -0.29 | 0.06 | 2.5E-06 | 22.16 |
|  | rs113958571 | 20 | A | G | 0.02 | -0.23 | 0.05 | 4.2E-06 | 21.18 |
|  | rs12567824 | 1 | A | C | 0.82 | 0.07 | 0.02 | 4.8E-06 | 20.93 |
|  | rs1356799 | 2 | T | G | 0.80 | -0.07 | 0.02 | 4.2E-06 | 21.16 |
|  | rs142915220 | 11 | A | G | 0.98 | -0.33 | 0.05 | 4.4E-11 | 43.44 |
|  | rs17099622 | 11 | A | G | 0.93 | -0.21 | 0.02 | 8.1E-19 | 78.47 |
|  | rs17860955 | 11 | T | C | 0.98 | 1.14 | 0.05 | 3E-129 | 585.33 |
|  | rs2584662 | 17 | A | C | 0.59 | -0.06 | 0.01 | 9.9E-07 | 23.95 |
|  | rs3742926 | 14 | T | C | 0.10 | -0.09 | 0.02 | 3.3E-06 | 21.65 |
|  | rs55804377 | 10 | A | G | 0.02 | -0.22 | 0.05 | 3.2E-06 | 21.70 |
|  | rs72699188 | 9 | C | G | 0.85 | -0.09 | 0.02 | 2E-08 | 31.45 |
|  | rs7966257 | 12 | C | G | 0.13 | -0.08 | 0.02 | 4.2E-06 | 21.17 |
| **Osteoprotegerin levels** | rs10142466 | 14 | A | G | 0.49 | 0.06 | 0.01 | 1.7E-07 | 27.35 |
|  | rs112635299 | 14 | T | G | 0.02 | 0.26 | 0.04 | 1.5E-09 | 36.56 |
|  | rs114165349 | 1 | C | G | 0.03 | -0.19 | 0.03 | 1E-08 | 32.83 |
|  | rs1230164 | 4 | C | G | 0.73 | -0.07 | 0.01 | 2E-07 | 27.06 |
|  | rs138408096 | 15 | A | G | 0.02 | -0.20 | 0.04 | 2.1E-06 | 22.51 |
|  | rs138557324 | 12 | T | C | 0.02 | -0.23 | 0.05 | 1.9E-06 | 22.69 |
|  | rs1437633 | 11 | A | C | 0.21 | -0.08 | 0.01 | 7.7E-08 | 28.88 |
|  | rs17600346 | 3 | T | C | 0.96 | -0.16 | 0.03 | 1.5E-07 | 27.54 |
|  | rs2247769 | 8 | T | C | 0.56 | -0.17 | 0.01 | 1.4E-50 | 223.69 |
|  | rs271665 | 17 | T | C | 0.75 | -0.07 | 0.01 | 1.9E-07 | 27.14 |
|  | rs28454196 | 4 | C | G | 0.98 | -0.22 | 0.05 | 4E-06 | 21.28 |
|  | rs376613127 | 12 | A | G | 0.01 | -0.27 | 0.06 | 2.1E-06 | 22.46 |
|  | rs61968587 | 13 | A | G | 0.98 | -0.27 | 0.06 | 2.5E-06 | 22.19 |
|  | rs6985207 | 8 | A | C | 0.56 | 0.05 | 0.01 | 4.7E-06 | 20.97 |
|  | rs704 | 17 | A | G | 0.47 | -0.14 | 0.01 | 1.1E-36 | 159.99 |
|  | rs74111611 | 12 | T | C | 0.96 | -0.15 | 0.03 | 8E-07 | 24.37 |
|  | rs77507094 | 10 | A | G | 0.98 | 0.20 | 0.04 | 4.6E-06 | 20.98 |
|  | rs9946923 | 18 | T | C | 0.97 | 0.17 | 0.04 | 4.8E-06 | 20.93 |
| **TNF-beta levels 40** | rs112317147 | 2 | T | C | 0.05 | -0.14 | 0.03 | 3.1E-06 | 21.75 |
|  | rs113244922 | 4 | T | C | 0.02 | 0.23 | 0.05 | 3.2E-06 | 21.69 |
|  | rs114250919 | 5 | T | C | 0.97 | 0.25 | 0.05 | 4.7E-07 | 25.39 |
|  | rs12743015 | 1 | C | G | 0.03 | -0.19 | 0.04 | 1.9E-06 | 22.65 |
|  | rs12883833 | 14 | A | G | 0.09 | -0.13 | 0.03 | 1.3E-06 | 23.37 |
|  | rs138471526 | 11 | T | C | 0.01 | -0.34 | 0.07 | 3.3E-06 | 21.63 |
|  | rs139511528 | 12 | A | G | 0.03 | -0.25 | 0.05 | 1.9E-06 | 22.75 |
|  | rs143019966 | 13 | T | C | 0.98 | 0.21 | 0.05 | 2.5E-06 | 22.14 |
|  | rs147423408 | 12 | A | G | 0.04 | 0.17 | 0.04 | 7.5E-07 | 24.49 |
|  | rs16852556 | 2 | T | C | 0.05 | 0.15 | 0.03 | 1.8E-06 | 22.81 |
|  | rs192303297 | 3 | T | C | 0.01 | -0.39 | 0.08 | 4.3E-06 | 21.15 |
|  | rs2074475 | 6 | T | C | 0.96 | 0.23 | 0.04 | 4.2E-08 | 30.05 |
|  | rs2364485 | 12 | A | C | 0.16 | 0.18 | 0.02 | 1.3E-20 | 86.62 |
|  | rs2523882 | 6 | T | C | 0.29 | -0.20 | 0.01 | 1.8E-43 | 191.17 |
|  | rs2844500 | 6 | T | C | 0.12 | -0.63 | 0.02 | 1E-200 | 1027.59 |
|  | rs28700215 | 15 | T | C | 0.58 | -0.06 | 0.01 | 4.2E-06 | 21.19 |
|  | rs537860389 | 13 | C | G | 0.97 | -0.23 | 0.05 | 3.1E-06 | 21.75 |
|  | rs55914821 | 17 | A | G | 0.76 | 0.07 | 0.02 | 2.6E-06 | 22.13 |
|  | rs72841573 | 6 | T | C | 0.98 | 0.28 | 0.06 | 5.1E-07 | 25.23 |
|  | rs74363807 | 7 | T | C | 0.02 | 0.25 | 0.05 | 2.7E-06 | 22.01 |
|  | rs7583622 | 2 | A | G | 0.21 | 0.07 | 0.02 | 3.4E-06 | 21.58 |
|  | rs76913603 | 5 | A | G | 0.97 | -0.17 | 0.04 | 4.4E-06 | 21.10 |
|  | rs77897196 | 1 | T | C | 0.97 | -0.19 | 0.04 | 2.9E-06 | 21.86 |
| **Precocious puberty** | rs146275068 | 2 | A | G | 0.01 | 1.40 | 0.30 | 3.4E-06 | 21.59 |
|  | rs711590 | 3 | T | C | 0.91 | -0.65 | 0.14 | 2.7E-06 | 22.04 |
|  | rs10213234 | 4 | C | T | 0.21 | -0.58 | 0.13 | 7.2E-06 | 20.13 |
|  | rs1022044 | 5 | T | C | 0.54 | 0.43 | 0.10 | 7E-06 | 20.19 |

SNP, single-nucleotide polymorphism; CHR, chromosome; EA, effect allele; OA, other allele; EAF, effect allele frequency; β, regression coefficient; SE, standard error; F-value: F=R2×(N−1−K)/((1−R2)×K).
