# Supplementary material for: The association between inflammation and pubertal development: Insights from NHANES and Mendelian randomization analyses
Source: Medicine (Baltimore). 2026 Oct 23;105(43):e51303. doi: 10.1097/MD.0000000000051303 (PMC13706194; doi:10.1097/MD.0000000000051303)
Supplement: Supplementary file 5 [file medi-105-e51303-s005.docx]

**Supplement 5.** Mendelian randomization analysis of the associations between 91 inflammatory proteins and precocious puberty.

| **ID** | **Exposure** | **Method** | **nSNP** | **β** | **SE** | **P-value** | **OR** | **LO_CI** | **UP_CI** |
| --- | --- | --- | --- | --- | --- | --- | --- | --- | --- |
| GCST90274758 | Eukaryotic translation initiation factor 4E-binding protein 1 levels | MR Egger | 11 | -2.30 | 1.05 | 0.0556 | 0.10 | 0.01 | 0.78 |
| GCST90274758 | Eukaryotic translation initiation factor 4E-binding protein 1 levels | Weighted median | 11 | -0.45 | 0.59 | 0.4472 | 0.64 | 0.20 | 2.03 |
| GCST90274758 | Eukaryotic translation initiation factor 4E-binding protein 1 levels | IVW | 11 | -0.45 | 0.44 | 0.3087 | 0.64 | 0.27 | 1.51 |
| GCST90274758 | Eukaryotic translation initiation factor 4E-binding protein 1 levels | Simple mode | 11 | -0.82 | 0.85 | 0.3574 | 0.44 | 0.08 | 2.34 |
| GCST90274758 | Eukaryotic translation initiation factor 4E-binding protein 1 levels | Weighted mode | 11 | -0.59 | 0.71 | 0.4293 | 0.56 | 0.14 | 2.25 |
| GCST90274759 | Adenosine Deaminase levels | MR Egger | 14 | -0.47 | 0.31 | 0.1519 | 0.62 | 0.34 | 1.14 |
| GCST90274759 | Adenosine Deaminase levels | Weighted median | 14 | -0.47 | 0.24 | 0.0530 | 0.62 | 0.39 | 1.01 |
| GCST90274759 | Adenosine Deaminase levels | IVW | 14 | -0.36 | 0.24 | 0.1331 | 0.70 | 0.44 | 1.11 |
| GCST90274759 | Adenosine Deaminase levels | Simple mode | 14 | -0.61 | 0.46 | 0.2091 | 0.54 | 0.22 | 1.34 |
| GCST90274759 | Adenosine Deaminase levels | Weighted mode | 14 | -0.49 | 0.25 | 0.0705 | 0.61 | 0.37 | 1.00 |
| GCST90274760 | Artemin levels | MR Egger | 17 | -1.78 | 0.96 | 0.0836 | 0.17 | 0.03 | 1.11 |
| GCST90274760 | Artemin levels | Weighted median | 17 | -0.32 | 0.52 | 0.5296 | 0.72 | 0.26 | 1.99 |
| GCST90274760 | Artemin levels | IVW | 17 | -0.62 | 0.37 | 0.0955 | 0.54 | 0.26 | 1.12 |
| GCST90274760 | Artemin levels | Simple mode | 17 | -0.21 | 0.89 | 0.8164 | 0.81 | 0.14 | 4.62 |
| GCST90274760 | Artemin levels | Weighted mode | 17 | -0.25 | 0.86 | 0.7697 | 0.78 | 0.14 | 4.15 |
| GCST90274761 | Axin-1 levels | MR Egger | 8 | -0.19 | 1.60 | 0.9074 | 0.82 | 0.04 | 18.89 |
| GCST90274761 | Axin-1 levels | Weighted median | 8 | -0.66 | 0.76 | 0.3877 | 0.52 | 0.12 | 2.30 |
| GCST90274761 | Axin-1 levels | IVW | 8 | -0.09 | 0.62 | 0.8833 | 0.91 | 0.27 | 3.05 |
| GCST90274761 | Axin-1 levels | Simple mode | 8 | -0.65 | 1.21 | 0.6097 | 0.52 | 0.05 | 5.65 |
| GCST90274761 | Axin-1 levels | Weighted mode | 8 | -0.75 | 1.05 | 0.4990 | 0.47 | 0.06 | 3.72 |
| GCST90274762 | beta-nerve growth factor levels | MR Egger | 21 | -1.21 | 1.00 | 0.2407 | 0.30 | 0.04 | 2.12 |
| GCST90274762 | beta-nerve growth factor levels | Weighted median | 21 | -0.47 | 0.55 | 0.3880 | 0.62 | 0.21 | 1.83 |
| GCST90274762 | beta-nerve growth factor levels | IVW | 21 | -0.60 | 0.39 | 0.1212 | 0.55 | 0.26 | 1.17 |
| GCST90274762 | beta-nerve growth factor levels | Simple mode | 21 | -0.38 | 0.91 | 0.6818 | 0.69 | 0.12 | 4.05 |
| GCST90274762 | beta-nerve growth factor levels | Weighted mode | 21 | -0.44 | 0.83 | 0.6010 | 0.64 | 0.13 | 3.29 |
| GCST90274763 | Caspase 8 levels | MR Egger | 14 | 1.12 | 0.76 | 0.1688 | 3.06 | 0.69 | 13.64 |
| GCST90274763 | Caspase 8 levels | Weighted median | 14 | 0.53 | 0.55 | 0.3294 | 1.70 | 0.58 | 4.96 |
| GCST90274763 | Caspase 8 levels | IVW | 14 | 0.25 | 0.44 | 0.5685 | 1.29 | 0.54 | 3.05 |
| GCST90274763 | Caspase 8 levels | Simple mode | 14 | 1.14 | 0.84 | 0.1989 | 3.12 | 0.60 | 16.23 |
| GCST90274763 | Caspase 8 levels | Weighted mode | 14 | 0.48 | 0.57 | 0.4157 | 1.62 | 0.53 | 4.95 |
| GCST90274764 | Eotaxin levels | MR Egger | 19 | 1.20 | 0.64 | 0.0761 | 3.32 | 0.96 | 11.55 |
| GCST90274764 | Eotaxin levels | Weighted median | 19 | 0.14 | 0.44 | 0.7476 | 1.15 | 0.48 | 2.75 |
| GCST90274764 | Eotaxin levels | IVW | 19 | 0.18 | 0.31 | 0.5674 | 1.20 | 0.65 | 2.22 |
| GCST90274764 | Eotaxin levels | Simple mode | 19 | -0.49 | 0.78 | 0.5382 | 0.61 | 0.13 | 2.83 |
| GCST90274764 | Eotaxin levels | Weighted mode | 19 | 0.98 | 0.53 | 0.0810 | 2.68 | 0.94 | 7.60 |
| GCST90274765 | C-C motif chemokine 19 levels | MR Egger | 20 | 0.03 | 0.38 | 0.9341 | 1.03 | 0.49 | 2.18 |
| GCST90274765 | C-C motif chemokine 19 levels | Weighted median | 20 | 0.29 | 0.32 | 0.3574 | 1.34 | 0.72 | 2.48 |
| GCST90274765 | C-C motif chemokine 19 levels | IVW | 20 | 0.19 | 0.25 | 0.4382 | 1.21 | 0.75 | 1.97 |
| GCST90274765 | C-C motif chemokine 19 levels | Simple mode | 20 | 0.17 | 0.59 | 0.7819 | 1.18 | 0.37 | 3.76 |
| GCST90274765 | C-C motif chemokine 19 levels | Weighted mode | 20 | 0.30 | 0.30 | 0.3339 | 1.35 | 0.75 | 2.44 |
| GCST90274766 | C-C motif chemokine 20 levels | MR Egger | 22 | -0.52 | 0.96 | 0.5952 | 0.60 | 0.09 | 3.90 |
| GCST90274766 | C-C motif chemokine 20 levels | Weighted median | 22 | 0.77 | 0.51 | 0.1305 | 2.16 | 0.80 | 5.86 |
| GCST90274766 | C-C motif chemokine 20 levels | IVW | 22 | 0.52 | 0.37 | 0.1640 | 1.68 | 0.81 | 3.48 |
| GCST90274766 | C-C motif chemokine 20 levels | Simple mode | 22 | -0.53 | 1.01 | 0.6085 | 0.59 | 0.08 | 4.28 |
| GCST90274766 | C-C motif chemokine 20 levels | Weighted mode | 22 | 1.20 | 0.86 | 0.1789 | 3.31 | 0.61 | 17.92 |
| GCST90274767 | C-C motif chemokine 23 levels | MR Egger | 22 | 0.33 | 0.32 | 0.3055 | 1.40 | 0.75 | 2.59 |
| GCST90274767 | C-C motif chemokine 23 levels | Weighted median | 22 | 0.22 | 0.28 | 0.4398 | 1.24 | 0.72 | 2.15 |
| GCST90274767 | C-C motif chemokine 23 levels | IVW | 22 | 0.13 | 0.22 | 0.5635 | 1.14 | 0.73 | 1.77 |
| GCST90274767 | C-C motif chemokine 23 levels | Simple mode | 22 | 0.63 | 0.61 | 0.3183 | 1.87 | 0.56 | 6.20 |
| GCST90274767 | C-C motif chemokine 23 levels | Weighted mode | 22 | 0.22 | 0.28 | 0.4390 | 1.25 | 0.72 | 2.17 |
| GCST90274768 | C-C motif chemokine 25 levels | MR Egger | 24 | -0.09 | 0.18 | 0.6280 | 0.92 | 0.64 | 1.30 |
| GCST90274768 | C-C motif chemokine 25 levels | Weighted median | 24 | -0.01 | 0.16 | 0.9727 | 0.99 | 0.73 | 1.35 |
| GCST90274768 | C-C motif chemokine 25 levels | IVW | 24 | -0.02 | 0.14 | 0.8588 | 0.98 | 0.74 | 1.28 |
| GCST90274768 | C-C motif chemokine 25 levels | Simple mode | 24 | 0.09 | 0.49 | 0.8552 | 1.09 | 0.42 | 2.87 |
| GCST90274768 | C-C motif chemokine 25 levels | Weighted mode | 24 | 0.02 | 0.15 | 0.9058 | 1.02 | 0.75 | 1.38 |
| GCST90274769 | C-C motif chemokine 28 levels | MR Egger | 20 | 0.84 | 0.85 | 0.3369 | 2.31 | 0.44 | 12.15 |
| GCST90274769 | C-C motif chemokine 28 levels | Weighted median | 20 | 0.44 | 0.54 | 0.4206 | 1.54 | 0.54 | 4.45 |
| GCST90274769 | C-C motif chemokine 28 levels | IVW | 20 | 0.18 | 0.40 | 0.6558 | 1.19 | 0.55 | 2.61 |
| GCST90274769 | C-C motif chemokine 28 levels | Simple mode | 20 | 1.15 | 1.01 | 0.2687 | 3.15 | 0.44 | 22.64 |
| GCST90274769 | C-C motif chemokine 28 levels | Weighted mode | 20 | 1.15 | 0.95 | 0.2437 | 3.15 | 0.49 | 20.40 |
| GCST90274770 | C-C motif chemokine 4 levels | MR Egger | 18 | -0.14 | 0.26 | 0.6104 | 0.87 | 0.52 | 1.46 |
| GCST90274770 | C-C motif chemokine 4 levels | Weighted median | 18 | -0.15 | 0.23 | 0.5131 | 0.86 | 0.55 | 1.34 |
| GCST90274770 | C-C motif chemokine 4 levels | IVW | 18 | -0.23 | 0.18 | 0.2028 | 0.80 | 0.56 | 1.13 |
| GCST90274770 | C-C motif chemokine 4 levels | Simple mode | 18 | -0.30 | 0.50 | 0.5501 | 0.74 | 0.28 | 1.96 |
| GCST90274770 | C-C motif chemokine 4 levels | Weighted mode | 18 | -0.14 | 0.20 | 0.4958 | 0.87 | 0.58 | 1.29 |
| GCST90274771 | Natural killer cell receptor 2B4 levels | MR Egger | 21 | -0.63 | 0.60 | 0.3075 | 0.53 | 0.16 | 1.73 |
| GCST90274771 | Natural killer cell receptor 2B4 levels | Weighted median | 21 | -0.05 | 0.42 | 0.9050 | 0.95 | 0.42 | 2.16 |
| GCST90274771 | Natural killer cell receptor 2B4 levels | IVW | 21 | 0.04 | 0.28 | 0.8931 | 1.04 | 0.60 | 1.81 |
| GCST90274771 | Natural killer cell receptor 2B4 levels | Simple mode | 21 | 0.22 | 0.75 | 0.7709 | 1.25 | 0.29 | 5.39 |
| GCST90274771 | Natural killer cell receptor 2B4 levels | Weighted mode | 21 | -0.12 | 0.45 | 0.7868 | 0.88 | 0.37 | 2.13 |
| GCST90274772 | CD40L receptor levels | MR Egger | 15 | -0.33 | 0.30 | 0.2956 | 0.72 | 0.40 | 1.30 |
| GCST90274772 | CD40L receptor levels | Weighted median | 15 | -0.02 | 0.23 | 0.9211 | 0.98 | 0.62 | 1.54 |
| GCST90274772 | CD40L receptor levels | IVW | 15 | -0.03 | 0.23 | 0.9083 | 0.97 | 0.63 | 1.52 |
| GCST90274772 | CD40L receptor levels | Simple mode | 15 | 1.27 | 0.84 | 0.1512 | 3.57 | 0.69 | 18.48 |
| GCST90274772 | CD40L receptor levels | Weighted mode | 15 | -0.02 | 0.25 | 0.9464 | 0.98 | 0.60 | 1.61 |
| GCST90274773 | T-cell surface glycoprotein CD5 levels | MR Egger | 21 | 0.26 | 0.81 | 0.7529 | 1.30 | 0.26 | 6.39 |
| GCST90274773 | T-cell surface glycoprotein CD5 levels | Weighted median | 21 | 0.42 | 0.42 | 0.3116 | 1.52 | 0.67 | 3.44 |
| GCST90274773 | T-cell surface glycoprotein CD5 levels | IVW | 21 | 0.26 | 0.30 | 0.3943 | 1.29 | 0.72 | 2.34 |
| GCST90274773 | T-cell surface glycoprotein CD5 levels | Simple mode | 21 | 0.32 | 0.69 | 0.6432 | 1.38 | 0.36 | 5.35 |
| GCST90274773 | T-cell surface glycoprotein CD5 levels | Weighted mode | 21 | 0.25 | 0.54 | 0.6444 | 1.29 | 0.45 | 3.70 |
| GCST90274774 | T-cell surface glycoprotein CD6 isoform levels | MR Egger | 18 | -0.30 | 0.31 | 0.3503 | 0.74 | 0.41 | 1.36 |
| GCST90274774 | T-cell surface glycoprotein CD6 isoform levels | Weighted median | 18 | -0.18 | 0.22 | 0.4085 | 0.83 | 0.54 | 1.29 |
| GCST90274774 | T-cell surface glycoprotein CD6 isoform levels | IVW | 18 | -0.08 | 0.18 | 0.6569 | 0.92 | 0.64 | 1.32 |
| GCST90274774 | T-cell surface glycoprotein CD6 isoform levels | Simple mode | 18 | -0.28 | 0.45 | 0.5448 | 0.76 | 0.32 | 1.82 |
| GCST90274774 | T-cell surface glycoprotein CD6 isoform levels | Weighted mode | 18 | -0.18 | 0.22 | 0.4233 | 0.84 | 0.55 | 1.28 |
| GCST90274775 | CUB domain-containing protein 1 levels | MR Egger | 25 | -0.02 | 0.39 | 0.9643 | 0.98 | 0.46 | 2.09 |
| GCST90274775 | CUB domain-containing protein 1 levels | Weighted median | 25 | -0.18 | 0.30 | 0.5499 | 0.83 | 0.46 | 1.51 |
| GCST90274775 | CUB domain-containing protein 1 levels | IVW | 25 | -0.33 | 0.22 | 0.1329 | 0.72 | 0.46 | 1.11 |
| GCST90274775 | CUB domain-containing protein 1 levels | Simple mode | 25 | -0.35 | 0.54 | 0.5316 | 0.71 | 0.24 | 2.06 |
| GCST90274775 | CUB domain-containing protein 1 levels | Weighted mode | 25 | -0.16 | 0.32 | 0.6166 | 0.85 | 0.45 | 1.59 |
| GCST90274776 | Macrophage colony-stimulating factor 1 levels | MR Egger | 16 | -0.38 | 1.30 | 0.7761 | 0.69 | 0.05 | 8.79 |
| GCST90274776 | Macrophage colony-stimulating factor 1 levels | Weighted median | 16 | 1.00 | 0.50 | 0.0445 | 2.73 | 1.02 | 7.25 |
| GCST90274776 | Macrophage colony-stimulating factor 1 levels | IVW | 16 | 0.00 | 0.50 | 0.9939 | 1.00 | 0.37 | 2.66 |
| GCST90274776 | Macrophage colony-stimulating factor 1 levels | Simple mode | 16 | 0.27 | 1.02 | 0.7928 | 1.31 | 0.18 | 9.75 |
| GCST90274776 | Macrophage colony-stimulating factor 1 levels | Weighted mode | 16 | 0.86 | 0.50 | 0.1023 | 2.37 | 0.90 | 6.29 |
| GCST90274777 | Cystatin D levels | MR Egger | 30 | -0.19 | 0.22 | 0.3952 | 0.83 | 0.54 | 1.27 |
| GCST90274777 | Cystatin D levels | Weighted median | 30 | -0.16 | 0.19 | 0.3897 | 0.85 | 0.58 | 1.23 |
| GCST90274777 | Cystatin D levels | IVW | 30 | -0.16 | 0.17 | 0.3364 | 0.85 | 0.61 | 1.18 |
| GCST90274777 | Cystatin D levels | Simple mode | 30 | -0.54 | 0.61 | 0.3846 | 0.58 | 0.18 | 1.93 |
| GCST90274777 | Cystatin D levels | Weighted mode | 30 | -0.20 | 0.20 | 0.3197 | 0.82 | 0.55 | 1.21 |
| GCST90274778 | Fractalkine levels | MR Egger | 21 | -0.73 | 0.94 | 0.4451 | 0.48 | 0.08 | 3.02 |
| GCST90274778 | Fractalkine levels | Weighted median | 21 | 0.01 | 0.50 | 0.9883 | 1.01 | 0.38 | 2.66 |
| GCST90274778 | Fractalkine levels | IVW | 21 | -0.23 | 0.36 | 0.5316 | 0.80 | 0.39 | 1.62 |
| GCST90274778 | Fractalkine levels | Simple mode | 21 | -0.29 | 0.88 | 0.7485 | 0.75 | 0.13 | 4.25 |
| GCST90274778 | Fractalkine levels | Weighted mode | 21 | -0.13 | 0.81 | 0.8735 | 0.88 | 0.18 | 4.26 |
| GCST90274779 | C-X-C motif chemokine 1 levels | MR Egger | 9 | -0.51 | 0.53 | 0.3682 | 0.60 | 0.21 | 1.70 |
| GCST90274779 | C-X-C motif chemokine 1 levels | Weighted median | 9 | -0.30 | 0.36 | 0.4088 | 0.74 | 0.36 | 1.51 |
| GCST90274779 | C-X-C motif chemokine 1 levels | IVW | 9 | -0.19 | 0.34 | 0.5693 | 0.82 | 0.42 | 1.60 |
| GCST90274779 | C-X-C motif chemokine 1 levels | Simple mode | 9 | -0.91 | 0.87 | 0.3278 | 0.40 | 0.07 | 2.22 |
| GCST90274779 | C-X-C motif chemokine 1 levels | Weighted mode | 9 | -0.34 | 0.37 | 0.3908 | 0.71 | 0.34 | 1.48 |
| GCST90274780 | C-X-C motif chemokine 10 levels | MR Egger | 23 | 0.34 | 0.61 | 0.5847 | 1.40 | 0.42 | 4.64 |
| GCST90274780 | C-X-C motif chemokine 10 levels | Weighted median | 23 | 0.24 | 0.43 | 0.5711 | 1.28 | 0.55 | 2.98 |
| GCST90274780 | C-X-C motif chemokine 10 levels | IVW | 23 | 0.35 | 0.33 | 0.2805 | 1.43 | 0.75 | 2.71 |
| GCST90274780 | C-X-C motif chemokine 10 levels | Simple mode | 23 | 1.07 | 0.86 | 0.2245 | 2.92 | 0.54 | 15.74 |
| GCST90274780 | C-X-C motif chemokine 10 levels | Weighted mode | 23 | 0.35 | 0.61 | 0.5730 | 1.42 | 0.43 | 4.67 |
| GCST90274781 | C-X-C motif chemokine 11 levels | MR Egger | 22 | -2.19 | 0.93 | 0.0292 | 0.11 | 0.02 | 0.70 |
| GCST90274781 | C-X-C motif chemokine 11 levels | Weighted median | 22 | -0.06 | 0.49 | 0.9086 | 0.95 | 0.36 | 2.47 |
| GCST90274781 | C-X-C motif chemokine 11 levels | IVW | 22 | -0.25 | 0.40 | 0.5223 | 0.78 | 0.36 | 1.68 |
| GCST90274781 | C-X-C motif chemokine 11 levels | Simple mode | 22 | 0.64 | 0.95 | 0.5072 | 1.90 | 0.29 | 12.33 |
| GCST90274781 | C-X-C motif chemokine 11 levels | Weighted mode | 22 | -0.79 | 0.54 | 0.1623 | 0.45 | 0.16 | 1.32 |
| GCST90274782 | C-X-C motif chemokine 5 levels | MR Egger | 14 | 0.12 | 0.41 | 0.7820 | 1.12 | 0.50 | 2.53 |
| GCST90274782 | C-X-C motif chemokine 5 levels | Weighted median | 14 | -0.05 | 0.29 | 0.8566 | 0.95 | 0.54 | 1.68 |
| GCST90274782 | C-X-C motif chemokine 5 levels | IVW | 14 | 0.00 | 0.25 | 0.9901 | 1.00 | 0.61 | 1.64 |
| GCST90274782 | C-X-C motif chemokine 5 levels | Simple mode | 14 | 0.55 | 0.65 | 0.4105 | 1.74 | 0.49 | 6.23 |
| GCST90274782 | C-X-C motif chemokine 5 levels | Weighted mode | 14 | 0.11 | 0.28 | 0.7113 | 1.11 | 0.64 | 1.93 |
| GCST90274783 | C-X-C motif chemokine 6 levels | MR Egger | 13 | -1.37 | 1.05 | 0.2166 | 0.25 | 0.03 | 1.98 |
| GCST90274783 | C-X-C motif chemokine 6 levels | Weighted median | 13 | 0.39 | 0.56 | 0.4851 | 1.48 | 0.49 | 4.43 |
| GCST90274783 | C-X-C motif chemokine 6 levels | IVW | 13 | -0.13 | 0.50 | 0.8012 | 0.88 | 0.33 | 2.36 |
| GCST90274783 | C-X-C motif chemokine 6 levels | Simple mode | 13 | 0.41 | 0.76 | 0.5963 | 1.51 | 0.34 | 6.69 |
| GCST90274783 | C-X-C motif chemokine 6 levels | Weighted mode | 13 | 0.31 | 0.60 | 0.6193 | 1.36 | 0.42 | 4.45 |
| GCST90274784 | C-X-C motif chemokine 9 levels | MR Egger | 21 | 0.15 | 0.82 | 0.8602 | 1.16 | 0.23 | 5.72 |
| GCST90274784 | C-X-C motif chemokine 9 levels | Weighted median | 21 | -0.33 | 0.47 | 0.4925 | 0.72 | 0.29 | 1.83 |
| GCST90274784 | C-X-C motif chemokine 9 levels | IVW | 21 | -0.18 | 0.34 | 0.5867 | 0.83 | 0.43 | 1.62 |
| GCST90274784 | C-X-C motif chemokine 9 levels | Simple mode | 21 | -0.61 | 0.77 | 0.4394 | 0.54 | 0.12 | 2.47 |
| GCST90274784 | C-X-C motif chemokine 9 levels | Weighted mode | 21 | -0.45 | 0.62 | 0.4709 | 0.64 | 0.19 | 2.13 |
| GCST90274785 | Delta and Notch-like epidermal growth factor-related receptor levels | MR Egger | 16 | -1.31 | 0.72 | 0.0894 | 0.27 | 0.07 | 1.10 |
| GCST90274785 | Delta and Notch-like epidermal growth factor-related receptor levels | Weighted median | 16 | -0.50 | 0.44 | 0.2527 | 0.61 | 0.26 | 1.43 |
| GCST90274785 | Delta and Notch-like epidermal growth factor-related receptor levels | IVW | 16 | -0.23 | 0.32 | 0.4737 | 0.80 | 0.43 | 1.49 |
| GCST90274785 | Delta and Notch-like epidermal growth factor-related receptor levels | Simple mode | 16 | -0.61 | 0.75 | 0.4303 | 0.55 | 0.13 | 2.36 |
| GCST90274785 | Delta and Notch-like epidermal growth factor-related receptor levels | Weighted mode | 16 | -0.66 | 0.46 | 0.1738 | 0.52 | 0.21 | 1.28 |
| GCST90274786 | Protein S100-A12 levels | MR Egger | 15 | 0.22 | 0.90 | 0.8122 | 1.25 | 0.21 | 7.32 |
| GCST90274786 | Protein S100-A12 levels | Weighted median | 15 | -0.19 | 0.54 | 0.7289 | 0.83 | 0.29 | 2.40 |
| GCST90274786 | Protein S100-A12 levels | IVW | 15 | -0.04 | 0.42 | 0.9279 | 0.96 | 0.42 | 2.21 |
| GCST90274786 | Protein S100-A12 levels | Simple mode | 15 | -0.24 | 0.84 | 0.7747 | 0.78 | 0.15 | 4.03 |
| GCST90274786 | Protein S100-A12 levels | Weighted mode | 15 | -0.16 | 0.58 | 0.7902 | 0.85 | 0.27 | 2.66 |
| GCST90274787 | Fibroblast growth factor 19 levels | MR Egger | 21 | -1.18 | 0.73 | 0.1249 | 0.31 | 0.07 | 1.30 |
| GCST90274787 | Fibroblast growth factor 19 levels | Weighted median | 21 | -0.64 | 0.43 | 0.1413 | 0.53 | 0.23 | 1.24 |
| GCST90274787 | Fibroblast growth factor 19 levels | IVW | 21 | -0.24 | 0.30 | 0.4289 | 0.79 | 0.44 | 1.42 |
| GCST90274787 | Fibroblast growth factor 19 levels | Simple mode | 21 | -0.62 | 0.74 | 0.4147 | 0.54 | 0.13 | 2.30 |
| GCST90274787 | Fibroblast growth factor 19 levels | Weighted mode | 21 | -0.67 | 0.51 | 0.2047 | 0.51 | 0.19 | 1.40 |
| GCST90274788 | Fibroblast growth factor 21 levels | MR Egger | 14 | 1.05 | 0.79 | 0.2060 | 2.86 | 0.61 | 13.39 |
| GCST90274788 | Fibroblast growth factor 21 levels | Weighted median | 14 | 0.09 | 0.45 | 0.8440 | 1.09 | 0.45 | 2.64 |
| GCST90274788 | Fibroblast growth factor 21 levels | IVW | 14 | 0.04 | 0.33 | 0.9048 | 1.04 | 0.54 | 2.00 |
| GCST90274788 | Fibroblast growth factor 21 levels | Simple mode | 14 | -0.29 | 0.75 | 0.7025 | 0.75 | 0.17 | 3.23 |
| GCST90274788 | Fibroblast growth factor 21 levels | Weighted mode | 14 | 0.13 | 0.51 | 0.7991 | 1.14 | 0.42 | 3.14 |
| GCST90274789 | Fibroblast growth factor 23 levels | MR Egger | 15 | 0.44 | 1.96 | 0.8253 | 1.55 | 0.03 | 71.88 |
| GCST90274789 | Fibroblast growth factor 23 levels | Weighted median | 15 | 1.15 | 0.61 | 0.0611 | 3.16 | 0.95 | 10.53 |
| GCST90274789 | Fibroblast growth factor 23 levels | IVW | 15 | 0.50 | 0.61 | 0.4146 | 1.65 | 0.50 | 5.47 |
| GCST90274789 | Fibroblast growth factor 23 levels | Simple mode | 15 | 1.94 | 1.38 | 0.1814 | 6.97 | 0.47 | 104.14 |
| GCST90274789 | Fibroblast growth factor 23 levels | Weighted mode | 15 | 1.16 | 1.05 | 0.2853 | 3.20 | 0.41 | 24.95 |
| GCST90274790 | Fibroblast growth factor 5 levels | MR Egger | 23 | -0.10 | 0.28 | 0.7266 | 0.91 | 0.52 | 1.57 |
| GCST90274790 | Fibroblast growth factor 5 levels | Weighted median | 23 | 0.06 | 0.20 | 0.7466 | 1.07 | 0.73 | 1.56 |
| GCST90274790 | Fibroblast growth factor 5 levels | IVW | 23 | -0.06 | 0.19 | 0.7533 | 0.94 | 0.65 | 1.37 |
| GCST90274790 | Fibroblast growth factor 5 levels | Simple mode | 23 | -0.45 | 0.67 | 0.5099 | 0.64 | 0.17 | 2.38 |
| GCST90274790 | Fibroblast growth factor 5 levels | Weighted mode | 23 | -0.01 | 0.20 | 0.9458 | 0.99 | 0.67 | 1.45 |
| GCST90274791 | Fms-related tyrosine kinase 3 ligand levels | MR Egger | 29 | -0.35 | 0.39 | 0.3773 | 0.70 | 0.33 | 1.52 |
| GCST90274791 | Fms-related tyrosine kinase 3 ligand levels | Weighted median | 29 | -0.15 | 0.37 | 0.6771 | 0.86 | 0.42 | 1.76 |
| GCST90274791 | Fms-related tyrosine kinase 3 ligand levels | IVW | 29 | -0.20 | 0.24 | 0.3990 | 0.82 | 0.51 | 1.30 |
| GCST90274791 | Fms-related tyrosine kinase 3 ligand levels | Simple mode | 29 | -0.82 | 0.74 | 0.2781 | 0.44 | 0.10 | 1.89 |
| GCST90274791 | Fms-related tyrosine kinase 3 ligand levels | Weighted mode | 29 | -0.30 | 0.39 | 0.4458 | 0.74 | 0.34 | 1.59 |
| GCST90274792 | Glial cell line-derived neurotrophic factor levels | MR Egger | 12 | 0.82 | 0.72 | 0.2812 | 2.27 | 0.55 | 9.26 |
| GCST90274792 | Glial cell line-derived neurotrophic factor levels | Weighted median | 12 | 0.24 | 0.41 | 0.5543 | 1.28 | 0.57 | 2.87 |
| GCST90274792 | Glial cell line-derived neurotrophic factor levels | IVW | 12 | 0.31 | 0.42 | 0.4585 | 1.36 | 0.60 | 3.10 |
| GCST90274792 | Glial cell line-derived neurotrophic factor levels | Simple mode | 12 | 0.78 | 0.76 | 0.3269 | 2.19 | 0.49 | 9.75 |
| GCST90274792 | Glial cell line-derived neurotrophic factor levels | Weighted mode | 12 | 0.28 | 0.44 | 0.5325 | 1.33 | 0.56 | 3.13 |
| GCST90274793 | Hepatocyte growth factor levels | MR Egger | 18 | -0.79 | 0.90 | 0.3911 | 0.45 | 0.08 | 2.63 |
| GCST90274793 | Hepatocyte growth factor levels | Weighted median | 18 | 0.47 | 0.53 | 0.3794 | 1.60 | 0.56 | 4.54 |
| GCST90274793 | Hepatocyte growth factor levels | IVW | 18 | 0.34 | 0.40 | 0.4064 | 1.40 | 0.63 | 3.08 |
| GCST90274793 | Hepatocyte growth factor levels | Simple mode | 18 | 0.95 | 0.92 | 0.3154 | 2.59 | 0.43 | 15.68 |
| GCST90274793 | Hepatocyte growth factor levels | Weighted mode | 18 | 0.45 | 0.78 | 0.5683 | 1.57 | 0.34 | 7.25 |
| GCST90274794 | Interferon gamma levels | MR Egger | 10 | -0.06 | 0.79 | 0.9406 | 0.94 | 0.20 | 4.46 |
| GCST90274794 | Interferon gamma levels | Weighted median | 10 | -0.01 | 0.56 | 0.9912 | 0.99 | 0.33 | 2.96 |
| GCST90274794 | Interferon gamma levels | IVW | 10 | -0.17 | 0.45 | 0.7044 | 0.84 | 0.35 | 2.04 |
| GCST90274794 | Interferon gamma levels | Simple mode | 10 | -0.02 | 0.90 | 0.9855 | 0.98 | 0.17 | 5.70 |
| GCST90274794 | Interferon gamma levels | Weighted mode | 10 | 0.04 | 0.90 | 0.9622 | 1.04 | 0.18 | 6.08 |
| GCST90274795 | Interleukin-10 levels | MR Egger | 18 | -0.72 | 0.73 | 0.3408 | 0.49 | 0.12 | 2.05 |
| GCST90274795 | Interleukin-10 levels | Weighted median | 18 | 0.38 | 0.49 | 0.4397 | 1.46 | 0.56 | 3.78 |
| GCST90274795 | Interleukin-10 levels | IVW | 18 | -0.04 | 0.37 | 0.9139 | 0.96 | 0.46 | 1.99 |
| GCST90274795 | Interleukin-10 levels | Simple mode | 18 | 0.58 | 0.86 | 0.5075 | 1.79 | 0.33 | 9.66 |
| GCST90274795 | Interleukin-10 levels | Weighted mode | 18 | 0.65 | 0.75 | 0.3994 | 1.92 | 0.44 | 8.40 |
| GCST90274796 | Interleukin-10 receptor subunit alpha levels | MR Egger | 10 | -0.36 | 0.62 | 0.5721 | 0.70 | 0.21 | 2.33 |
| GCST90274796 | Interleukin-10 receptor subunit alpha levels | Weighted median | 10 | 0.26 | 0.54 | 0.6321 | 1.30 | 0.45 | 3.76 |
| GCST90274796 | Interleukin-10 receptor subunit alpha levels | IVW | 10 | 0.04 | 0.38 | 0.9255 | 1.04 | 0.49 | 2.20 |
| GCST90274796 | Interleukin-10 receptor subunit alpha levels | Simple mode | 10 | 1.11 | 0.89 | 0.2454 | 3.03 | 0.53 | 17.47 |
| GCST90274796 | Interleukin-10 receptor subunit alpha levels | Weighted mode | 10 | -1.06 | 0.70 | 0.1657 | 0.35 | 0.09 | 1.37 |
| GCST90274797 | Interleukin-10 receptor subunit beta levels | MR Egger | 18 | 0.61 | 0.30 | 0.0553 | 1.85 | 1.03 | 3.31 |
| GCST90274797 | Interleukin-10 receptor subunit beta levels | Weighted median | 18 | 0.49 | 0.24 | 0.0420 | 1.63 | 1.02 | 2.61 |
| GCST90274797 | Interleukin-10 receptor subunit beta levels | IVW | 18 | 0.30 | 0.20 | 0.1397 | 1.35 | 0.91 | 2.01 |
| GCST90274797 | Interleukin-10 receptor subunit beta levels | Simple mode | 18 | 0.19 | 0.61 | 0.7542 | 1.21 | 0.37 | 3.99 |
| GCST90274797 | Interleukin-10 receptor subunit beta levels | Weighted mode | 18 | 0.51 | 0.26 | 0.0651 | 1.66 | 1.00 | 2.75 |
| GCST90274798 | Interleukin-12 subunit beta levels | MR Egger | 28 | 0.06 | 0.26 | 0.8028 | 1.07 | 0.65 | 1.76 |
| GCST90274798 | Interleukin-12 subunit beta levels | Weighted median | 28 | 0.19 | 0.21 | 0.3476 | 1.21 | 0.81 | 1.82 |
| GCST90274798 | Interleukin-12 subunit beta levels | IVW | 28 | 0.25 | 0.16 | 0.1309 | 1.28 | 0.93 | 1.76 |
| GCST90274798 | Interleukin-12 subunit beta levels | Simple mode | 28 | 0.85 | 0.52 | 0.1119 | 2.34 | 0.85 | 6.45 |
| GCST90274798 | Interleukin-12 subunit beta levels | Weighted mode | 28 | 0.22 | 0.19 | 0.2390 | 1.25 | 0.87 | 1.80 |
| GCST90274799 | Interleukin-13 levels | MR Egger | 11 | 0.78 | 0.88 | 0.3956 | 2.19 | 0.39 | 12.20 |
| GCST90274799 | Interleukin-13 levels | Weighted median | 11 | 0.04 | 0.50 | 0.9313 | 1.04 | 0.39 | 2.77 |
| GCST90274799 | Interleukin-13 levels | IVW | 11 | -0.29 | 0.46 | 0.5198 | 0.75 | 0.31 | 1.82 |
| GCST90274799 | Interleukin-13 levels | Simple mode | 11 | 0.15 | 0.65 | 0.8173 | 1.17 | 0.33 | 4.13 |
| GCST90274799 | Interleukin-13 levels | Weighted mode | 11 | 0.17 | 0.62 | 0.7896 | 1.19 | 0.35 | 4.00 |
| GCST90274800 | Interleukin-15 receptor subunit alpha levels | MR Egger | 12 | -0.17 | 0.37 | 0.6651 | 0.85 | 0.41 | 1.76 |
| GCST90274800 | Interleukin-15 receptor subunit alpha levels | Weighted median | 12 | -0.14 | 0.24 | 0.5654 | 0.87 | 0.54 | 1.40 |
| GCST90274800 | Interleukin-15 receptor subunit alpha levels | IVW | 12 | -0.26 | 0.22 | 0.2229 | 0.77 | 0.50 | 1.17 |
| GCST90274800 | Interleukin-15 receptor subunit alpha levels | Simple mode | 12 | 0.11 | 0.50 | 0.8245 | 1.12 | 0.42 | 2.98 |
| GCST90274800 | Interleukin-15 receptor subunit alpha levels | Weighted mode | 12 | -0.16 | 0.24 | 0.5191 | 0.85 | 0.53 | 1.36 |
| GCST90274801 | Interleukin-17A levels | MR Egger | 12 | 1.35 | 1.59 | 0.4167 | 3.86 | 0.17 | 87.83 |
| GCST90274801 | Interleukin-17A levels | Weighted median | 12 | 0.67 | 0.66 | 0.3096 | 1.96 | 0.54 | 7.17 |
| GCST90274801 | Interleukin-17A levels | IVW | 12 | 0.27 | 0.60 | 0.6540 | 1.31 | 0.40 | 4.29 |
| GCST90274801 | Interleukin-17A levels | Simple mode | 12 | 1.61 | 1.21 | 0.2103 | 4.98 | 0.47 | 52.99 |
| GCST90274801 | Interleukin-17A levels | Weighted mode | 12 | 1.47 | 1.14 | 0.2227 | 4.37 | 0.47 | 40.92 |
| GCST90274802 | Interleukin-17C levels | MR Egger | 17 | -0.24 | 1.14 | 0.8373 | 0.79 | 0.09 | 7.30 |
| GCST90274802 | Interleukin-17C levels | Weighted median | 17 | -0.22 | 0.50 | 0.6612 | 0.80 | 0.30 | 2.14 |
| GCST90274802 | Interleukin-17C levels | IVW | 17 | -0.22 | 0.37 | 0.5577 | 0.81 | 0.39 | 1.66 |
| GCST90274802 | Interleukin-17C levels | Simple mode | 17 | -0.20 | 0.92 | 0.8337 | 0.82 | 0.14 | 4.95 |
| GCST90274802 | Interleukin-17C levels | Weighted mode | 17 | -0.38 | 0.74 | 0.6164 | 0.68 | 0.16 | 2.93 |
| GCST90274803 | Interleukin-18 levels | MR Egger | 21 | 0.22 | 0.58 | 0.7039 | 1.25 | 0.40 | 3.90 |
| GCST90274803 | Interleukin-18 levels | Weighted median | 21 | -0.03 | 0.36 | 0.9342 | 0.97 | 0.48 | 1.96 |
| GCST90274803 | Interleukin-18 levels | IVW | 21 | 0.02 | 0.27 | 0.9429 | 1.02 | 0.60 | 1.73 |
| GCST90274803 | Interleukin-18 levels | Simple mode | 21 | -0.53 | 0.58 | 0.3677 | 0.59 | 0.19 | 1.82 |
| GCST90274803 | Interleukin-18 levels | Weighted mode | 21 | -0.06 | 0.39 | 0.8875 | 0.95 | 0.44 | 2.02 |
| GCST90274804 | interleukin-18 receptor 1 levels | MR Egger | 20 | -0.77 | 0.48 | 0.1240 | 0.46 | 0.18 | 1.18 |
| GCST90274804 | interleukin-18 receptor 1 levels | Weighted median | 20 | -0.54 | 0.26 | 0.0381 | 0.58 | 0.35 | 0.97 |
| GCST90274804 | interleukin-18 receptor 1 levels | IVW | 20 | -0.37 | 0.25 | 0.1341 | 0.69 | 0.42 | 1.12 |
| GCST90274804 | interleukin-18 receptor 1 levels | Simple mode | 20 | -0.19 | 0.54 | 0.7237 | 0.82 | 0.29 | 2.37 |
| GCST90274804 | interleukin-18 receptor 1 levels | Weighted mode | 20 | -0.50 | 0.26 | 0.0730 | 0.61 | 0.36 | 1.02 |
| GCST90274805 | Interleukin-1-alpha levels | MR Egger | 12 | 0.81 | 0.90 | 0.3893 | 2.25 | 0.38 | 13.16 |
| GCST90274805 | Interleukin-1-alpha levels | Weighted median | 12 | 0.25 | 0.60 | 0.6736 | 1.29 | 0.40 | 4.21 |
| GCST90274805 | Interleukin-1-alpha levels | IVW | 12 | -0.03 | 0.46 | 0.9558 | 0.97 | 0.39 | 2.42 |
| GCST90274805 | Interleukin-1-alpha levels | Simple mode | 12 | 0.42 | 0.98 | 0.6739 | 1.53 | 0.22 | 10.44 |
| GCST90274805 | Interleukin-1-alpha levels | Weighted mode | 12 | 0.35 | 0.66 | 0.6081 | 1.41 | 0.39 | 5.11 |
| GCST90274806 | Interleukin-2 levels | MR Egger | 15 | -0.43 | 1.03 | 0.6800 | 0.65 | 0.09 | 4.87 |
| GCST90274806 | Interleukin-2 levels | Weighted median | 15 | 0.12 | 0.52 | 0.8162 | 1.13 | 0.41 | 3.13 |
| GCST90274806 | Interleukin-2 levels | IVW | 15 | 0.06 | 0.39 | 0.8685 | 1.07 | 0.50 | 2.29 |
| GCST90274806 | Interleukin-2 levels | Simple mode | 15 | 0.51 | 0.91 | 0.5805 | 1.67 | 0.28 | 9.91 |
| GCST90274806 | Interleukin-2 levels | Weighted mode | 15 | -0.10 | 0.94 | 0.9132 | 0.90 | 0.14 | 5.69 |
| GCST90274807 | Interleukin-20 levels | MR Egger | 11 | -0.17 | 1.02 | 0.8717 | 0.84 | 0.11 | 6.25 |
| GCST90274807 | Interleukin-20 levels | Weighted median | 11 | -0.03 | 0.64 | 0.9649 | 0.97 | 0.28 | 3.38 |
| GCST90274807 | Interleukin-20 levels | IVW | 11 | 0.33 | 0.48 | 0.4911 | 1.39 | 0.54 | 3.54 |
| GCST90274807 | Interleukin-20 levels | Simple mode | 11 | 0.58 | 0.95 | 0.5564 | 1.78 | 0.28 | 11.52 |
| GCST90274807 | Interleukin-20 levels | Weighted mode | 11 | -0.12 | 0.83 | 0.8842 | 0.88 | 0.17 | 4.51 |
| GCST90274808 | Interleukin-20 receptor subunit alpha levels | MR Egger | 11 | -0.56 | 0.89 | 0.5464 | 0.57 | 0.10 | 3.29 |
| GCST90274808 | Interleukin-20 receptor subunit alpha levels | Weighted median | 11 | -0.51 | 0.55 | 0.3586 | 0.60 | 0.20 | 1.78 |
| GCST90274808 | Interleukin-20 receptor subunit alpha levels | IVW | 11 | -0.45 | 0.42 | 0.2918 | 0.64 | 0.28 | 1.47 |
| GCST90274808 | Interleukin-20 receptor subunit alpha levels | Simple mode | 11 | -0.19 | 0.87 | 0.8283 | 0.82 | 0.15 | 4.50 |
| GCST90274808 | Interleukin-20 receptor subunit alpha levels | Weighted mode | 11 | -0.57 | 0.71 | 0.4361 | 0.56 | 0.14 | 2.25 |
| GCST90274809 | Interleukin-22 receptor subunit alpha-1 levels | MR Egger | 11 | -0.58 | 1.00 | 0.5760 | 0.56 | 0.08 | 3.97 |
| GCST90274809 | Interleukin-22 receptor subunit alpha-1 levels | Weighted median | 11 | -0.84 | 0.71 | 0.2337 | 0.43 | 0.11 | 1.72 |
| GCST90274809 | Interleukin-22 receptor subunit alpha-1 levels | IVW | 11 | -0.75 | 0.53 | 0.1538 | 0.47 | 0.17 | 1.33 |
| GCST90274809 | Interleukin-22 receptor subunit alpha-1 levels | Simple mode | 11 | -0.37 | 0.96 | 0.7077 | 0.69 | 0.10 | 4.54 |
| GCST90274809 | Interleukin-22 receptor subunit alpha-1 levels | Weighted mode | 11 | -1.87 | 0.89 | 0.0616 | 0.15 | 0.03 | 0.88 |
| GCST90274810 | Interleukin-24 levels | MR Egger | 10 | 1.14 | 1.71 | 0.5234 | 3.12 | 0.11 | 88.28 |
| GCST90274810 | Interleukin-24 levels | Weighted median | 10 | -0.92 | 0.84 | 0.2709 | 0.40 | 0.08 | 2.05 |
| GCST90274810 | Interleukin-24 levels | IVW | 10 | -0.15 | 0.71 | 0.8295 | 0.86 | 0.21 | 3.43 |
| GCST90274810 | Interleukin-24 levels | Simple mode | 10 | -2.03 | 1.61 | 0.2388 | 0.13 | 0.01 | 3.08 |
| GCST90274810 | Interleukin-24 levels | Weighted mode | 10 | -1.59 | 1.23 | 0.2278 | 0.20 | 0.02 | 2.26 |
| GCST90274811 | Interleukin-2 receptor subunit beta levels | MR Egger | 12 | -1.99 | 1.02 | 0.0803 | 0.14 | 0.02 | 1.01 |
| GCST90274811 | Interleukin-2 receptor subunit beta levels | Weighted median | 12 | -0.52 | 0.67 | 0.4396 | 0.59 | 0.16 | 2.22 |
| GCST90274811 | Interleukin-2 receptor subunit beta levels | IVW | 12 | -0.26 | 0.50 | 0.6057 | 0.77 | 0.29 | 2.05 |
| GCST90274811 | Interleukin-2 receptor subunit beta levels | Simple mode | 12 | -0.33 | 1.12 | 0.7767 | 0.72 | 0.08 | 6.54 |
| GCST90274811 | Interleukin-2 receptor subunit beta levels | Weighted mode | 12 | -0.65 | 1.04 | 0.5480 | 0.52 | 0.07 | 4.06 |
| GCST90274812 | Interleukin-33 levels | MR Egger | 11 | 1.01 | 1.37 | 0.4806 | 2.75 | 0.19 | 40.59 |
| GCST90274812 | Interleukin-33 levels | Weighted median | 11 | 0.28 | 0.65 | 0.6692 | 1.32 | 0.37 | 4.72 |
| GCST90274812 | Interleukin-33 levels | IVW | 11 | 0.42 | 0.51 | 0.4095 | 1.52 | 0.56 | 4.16 |
| GCST90274812 | Interleukin-33 levels | Simple mode | 11 | -0.48 | 1.28 | 0.7142 | 0.62 | 0.05 | 7.61 |
| GCST90274812 | Interleukin-33 levels | Weighted mode | 11 | -0.57 | 1.38 | 0.6871 | 0.56 | 0.04 | 8.45 |
| GCST90274813 | Interleukin-4 levels | MR Egger | 9 | 1.76 | 1.25 | 0.2022 | 5.82 | 0.50 | 67.68 |
| GCST90274813 | Interleukin-4 levels | Weighted median | 9 | 0.48 | 0.69 | 0.4857 | 1.62 | 0.42 | 6.23 |
| GCST90274813 | Interleukin-4 levels | IVW | 9 | 0.29 | 0.50 | 0.5629 | 1.33 | 0.50 | 3.53 |
| GCST90274813 | Interleukin-4 levels | Simple mode | 9 | 0.93 | 1.11 | 0.4262 | 2.54 | 0.29 | 22.47 |
| GCST90274813 | Interleukin-4 levels | Weighted mode | 9 | 1.08 | 1.03 | 0.3274 | 2.94 | 0.39 | 22.33 |
| GCST90274814 | Interleukin-5 levels | MR Egger | 11 | 0.49 | 0.81 | 0.5606 | 1.63 | 0.33 | 7.99 |
| GCST90274814 | Interleukin-5 levels | Weighted median | 11 | 0.07 | 0.61 | 0.9061 | 1.07 | 0.33 | 3.52 |
| GCST90274814 | Interleukin-5 levels | IVW | 11 | -0.24 | 0.43 | 0.5816 | 0.79 | 0.34 | 1.83 |
| GCST90274814 | Interleukin-5 levels | Simple mode | 11 | 0.37 | 0.83 | 0.6632 | 1.45 | 0.28 | 7.40 |
| GCST90274814 | Interleukin-5 levels | Weighted mode | 11 | 0.16 | 0.72 | 0.8302 | 1.17 | 0.28 | 4.83 |
| GCST90274815 | Interleukin-6 levels | MR Egger | 8 | 0.13 | 0.90 | 0.8889 | 1.14 | 0.20 | 6.60 |
| GCST90274815 | Interleukin-6 levels | Weighted median | 8 | 0.15 | 0.54 | 0.7790 | 1.16 | 0.41 | 3.32 |
| GCST90274815 | Interleukin-6 levels | IVW | 8 | 0.06 | 0.45 | 0.9034 | 1.06 | 0.43 | 2.57 |
| GCST90274815 | Interleukin-6 levels | Simple mode | 8 | 0.31 | 0.81 | 0.7111 | 1.37 | 0.28 | 6.76 |
| GCST90274815 | Interleukin-6 levels | Weighted mode | 8 | 0.18 | 0.58 | 0.7710 | 1.19 | 0.38 | 3.71 |
| GCST90274816 | Interleukin-7 levels | MR Egger | 12 | 2.02 | 1.59 | 0.2328 | 7.51 | 0.33 | 168.36 |
| GCST90274816 | Interleukin-7 levels | Weighted median | 12 | -0.85 | 0.71 | 0.2294 | 0.43 | 0.11 | 1.71 |
| GCST90274816 | Interleukin-7 levels | IVW | 12 | -0.63 | 0.51 | 0.2154 | 0.53 | 0.20 | 1.44 |
| GCST90274816 | Interleukin-7 levels | Simple mode | 12 | -1.03 | 1.26 | 0.4301 | 0.36 | 0.03 | 4.21 |
| GCST90274816 | Interleukin-7 levels | Weighted mode | 12 | -1.03 | 1.23 | 0.4189 | 0.36 | 0.03 | 3.96 |
| GCST90274817 | Interleukin-8 levels | MR Egger | 17 | 0.44 | 0.81 | 0.5911 | 1.56 | 0.32 | 7.55 |
| GCST90274817 | Interleukin-8 levels | Weighted median | 17 | 0.53 | 0.55 | 0.3382 | 1.69 | 0.58 | 4.98 |
| GCST90274817 | Interleukin-8 levels | IVW | 17 | 0.32 | 0.40 | 0.4310 | 1.37 | 0.62 | 3.04 |
| GCST90274817 | Interleukin-8 levels | Simple mode | 17 | 0.31 | 0.91 | 0.7398 | 1.36 | 0.23 | 8.12 |
| GCST90274817 | Interleukin-8 levels | Weighted mode | 17 | 0.73 | 0.76 | 0.3516 | 2.07 | 0.47 | 9.11 |
| GCST90274818 | Latency-associated peptide transforming growth factor beta 1 levels | MR Egger | 19 | 1.14 | 0.57 | 0.0611 | 3.11 | 1.03 | 9.45 |
| GCST90274818 | Latency-associated peptide transforming growth factor beta 1 levels | Weighted median | 19 | 0.50 | 0.46 | 0.2820 | 1.65 | 0.66 | 4.09 |
| GCST90274818 | Latency-associated peptide transforming growth factor beta 1 levels | IVW | 19 | 0.58 | 0.33 | 0.0770 | 1.79 | 0.94 | 3.40 |
| GCST90274818 | Latency-associated peptide transforming growth factor beta 1 levels | Simple mode | 19 | 0.28 | 0.67 | 0.6781 | 1.32 | 0.36 | 4.88 |
| GCST90274818 | Latency-associated peptide transforming growth factor beta 1 levels | Weighted mode | 19 | 0.42 | 0.49 | 0.4110 | 1.52 | 0.58 | 4.00 |
| GCST90274819 | Leukemia inhibitory factor levels | MR Egger | 11 | 1.45 | 1.04 | 0.1988 | 4.26 | 0.55 | 32.96 |
| GCST90274819 | Leukemia inhibitory factor levels | Weighted median | 11 | -0.13 | 0.65 | 0.8382 | 0.88 | 0.25 | 3.12 |
| GCST90274819 | Leukemia inhibitory factor levels | IVW | 11 | 0.21 | 0.52 | 0.6832 | 1.23 | 0.45 | 3.39 |
| GCST90274819 | Leukemia inhibitory factor levels | Simple mode | 11 | 0.91 | 1.19 | 0.4611 | 2.49 | 0.24 | 25.64 |
| GCST90274819 | Leukemia inhibitory factor levels | Weighted mode | 11 | -0.43 | 0.94 | 0.6553 | 0.65 | 0.10 | 4.08 |
| GCST90274820 | Leukemia inhibitory factor receptor levels | MR Egger | 16 | 0.59 | 0.52 | 0.2696 | 1.81 | 0.66 | 4.99 |
| GCST90274820 | Leukemia inhibitory factor receptor levels | Weighted median | 16 | -0.40 | 0.36 | 0.2701 | 0.67 | 0.33 | 1.36 |
| GCST90274820 | Leukemia inhibitory factor receptor levels | IVW | 16 | -0.44 | 0.27 | 0.1074 | 0.64 | 0.38 | 1.10 |
| GCST90274820 | Leukemia inhibitory factor receptor levels | Simple mode | 16 | -0.90 | 0.67 | 0.2029 | 0.41 | 0.11 | 1.53 |
| GCST90274820 | Leukemia inhibitory factor receptor levels | Weighted mode | 16 | -0.01 | 0.36 | 0.9808 | 0.99 | 0.49 | 2.02 |
| GCST90274821 | Monocyte chemoattractant protein-1 levels | MR Egger | 22 | 0.27 | 0.42 | 0.5182 | 1.31 | 0.58 | 2.96 |
| GCST90274821 | Monocyte chemoattractant protein-1 levels | Weighted median | 22 | 0.12 | 0.34 | 0.7368 | 1.12 | 0.57 | 2.20 |
| GCST90274821 | Monocyte chemoattractant protein-1 levels | IVW | 22 | -0.02 | 0.24 | 0.9247 | 0.98 | 0.61 | 1.57 |
| GCST90274821 | Monocyte chemoattractant protein-1 levels | Simple mode | 22 | 0.53 | 0.50 | 0.3032 | 1.70 | 0.63 | 4.58 |
| GCST90274821 | Monocyte chemoattractant protein-1 levels | Weighted mode | 22 | -0.04 | 0.35 | 0.9027 | 0.96 | 0.48 | 1.91 |
| GCST90274822 | Monocyte chemoattractant protein 2 levels | MR Egger | 28 | -0.22 | 0.20 | 0.2887 | 0.80 | 0.54 | 1.19 |
| GCST90274822 | Monocyte chemoattractant protein 2 levels | Weighted median | 28 | 0.15 | 0.21 | 0.4892 | 1.16 | 0.76 | 1.77 |
| GCST90274822 | Monocyte chemoattractant protein 2 levels | IVW | 28 | -0.03 | 0.16 | 0.8622 | 0.97 | 0.71 | 1.33 |
| GCST90274822 | Monocyte chemoattractant protein 2 levels | Simple mode | 28 | 0.85 | 0.42 | 0.0524 | 2.33 | 1.03 | 5.29 |
| GCST90274822 | Monocyte chemoattractant protein 2 levels | Weighted mode | 28 | 0.09 | 0.18 | 0.6240 | 1.09 | 0.77 | 1.56 |
| GCST90274823 | Monocyte chemoattractant protein-3 levels | MR Egger | 17 | 0.27 | 0.75 | 0.7292 | 1.30 | 0.30 | 5.70 |
| GCST90274823 | Monocyte chemoattractant protein-3 levels | Weighted median | 17 | 0.56 | 0.43 | 0.1967 | 1.74 | 0.75 | 4.05 |
| GCST90274823 | Monocyte chemoattractant protein-3 levels | IVW | 17 | 0.25 | 0.34 | 0.4677 | 1.28 | 0.66 | 2.51 |
| GCST90274823 | Monocyte chemoattractant protein-3 levels | Simple mode | 17 | 0.66 | 0.83 | 0.4358 | 1.94 | 0.38 | 9.81 |
| GCST90274823 | Monocyte chemoattractant protein-3 levels | Weighted mode | 17 | 0.89 | 0.58 | 0.1437 | 2.44 | 0.78 | 7.62 |
| GCST90274824 | Monocyte chemoattractant protein-4 levels | MR Egger | 18 | -0.34 | 0.50 | 0.5012 | 0.71 | 0.27 | 1.88 |
| GCST90274824 | Monocyte chemoattractant protein-4 levels | Weighted median | 18 | -0.36 | 0.35 | 0.2927 | 0.69 | 0.35 | 1.37 |
| GCST90274824 | Monocyte chemoattractant protein-4 levels | IVW | 18 | -0.25 | 0.24 | 0.2986 | 0.78 | 0.48 | 1.25 |
| GCST90274824 | Monocyte chemoattractant protein-4 levels | Simple mode | 18 | -0.71 | 0.55 | 0.2153 | 0.49 | 0.17 | 1.45 |
| GCST90274824 | Monocyte chemoattractant protein-4 levels | Weighted mode | 18 | -0.35 | 0.38 | 0.3688 | 0.71 | 0.34 | 1.48 |
| GCST90274825 | Macrophage inflammatory protein 1a levels | MR Egger | 14 | -0.08 | 0.46 | 0.8692 | 0.93 | 0.38 | 2.28 |
| GCST90274825 | Macrophage inflammatory protein 1a levels | Weighted median | 14 | -0.02 | 0.30 | 0.9399 | 0.98 | 0.54 | 1.77 |
| GCST90274825 | Macrophage inflammatory protein 1a levels | IVW | 14 | -0.06 | 0.28 | 0.8171 | 0.94 | 0.55 | 1.61 |
| GCST90274825 | Macrophage inflammatory protein 1a levels | Simple mode | 14 | -0.33 | 0.60 | 0.5855 | 0.72 | 0.22 | 2.31 |
| GCST90274825 | Macrophage inflammatory protein 1a levels | Weighted mode | 14 | -0.03 | 0.30 | 0.9336 | 0.97 | 0.54 | 1.75 |
| GCST90274826 | Matrix metalloproteinase-1 levels | MR Egger | 16 | -0.04 | 0.60 | 0.9414 | 0.96 | 0.30 | 3.09 |
| GCST90274826 | Matrix metalloproteinase-1 levels | Weighted median | 16 | -0.39 | 0.44 | 0.3678 | 0.67 | 0.29 | 1.59 |
| GCST90274826 | Matrix metalloproteinase-1 levels | IVW | 16 | -0.06 | 0.33 | 0.8662 | 0.95 | 0.50 | 1.80 |
| GCST90274826 | Matrix metalloproteinase-1 levels | Simple mode | 16 | -0.56 | 0.70 | 0.4350 | 0.57 | 0.15 | 2.24 |
| GCST90274826 | Matrix metalloproteinase-1 levels | Weighted mode | 16 | -0.48 | 0.53 | 0.3732 | 0.62 | 0.22 | 1.73 |
| GCST90274827 | Matrix metalloproteinase-10 levels | MR Egger | 14 | 0.94 | 0.49 | 0.0775 | 2.55 | 0.99 | 6.61 |
| GCST90274827 | Matrix metalloproteinase-10 levels | Weighted median | 14 | 0.85 | 0.41 | 0.0403 | 2.34 | 1.04 | 5.26 |
| GCST90274827 | Matrix metalloproteinase-10 levels | IVW | 14 | 0.79 | 0.30 | 0.0092 | 2.21 | 1.22 | 4.02 |
| GCST90274827 | Matrix metalloproteinase-10 levels | Simple mode | 14 | 1.37 | 0.76 | 0.0945 | 3.93 | 0.89 | 17.34 |
| GCST90274827 | Matrix metalloproteinase-10 levels | Weighted mode | 14 | 0.88 | 0.42 | 0.0574 | 2.41 | 1.05 | 5.49 |
| GCST90274828 | Neurturin levels | MR Egger | 14 | -0.61 | 0.62 | 0.3414 | 0.54 | 0.16 | 1.82 |
| GCST90274828 | Neurturin levels | Weighted median | 14 | -0.23 | 0.48 | 0.6350 | 0.80 | 0.31 | 2.03 |
| GCST90274828 | Neurturin levels | IVW | 14 | -0.32 | 0.35 | 0.3523 | 0.73 | 0.37 | 1.43 |
| GCST90274828 | Neurturin levels | Simple mode | 14 | -0.17 | 0.73 | 0.8196 | 0.84 | 0.20 | 3.51 |
| GCST90274828 | Neurturin levels | Weighted mode | 14 | -0.22 | 0.65 | 0.7403 | 0.80 | 0.23 | 2.86 |
| GCST90274829 | Neurotrophin-3 levels | MR Egger | 17 | 1.26 | 0.89 | 0.1785 | 3.53 | 0.61 | 20.27 |
| GCST90274829 | Neurotrophin-3 levels | Weighted median | 17 | 0.14 | 0.57 | 0.8043 | 1.15 | 0.38 | 3.54 |
| GCST90274829 | Neurotrophin-3 levels | IVW | 17 | 0.05 | 0.40 | 0.9077 | 1.05 | 0.48 | 2.30 |
| GCST90274829 | Neurotrophin-3 levels | Simple mode | 17 | 0.39 | 1.04 | 0.7097 | 1.48 | 0.19 | 11.44 |
| GCST90274829 | Neurotrophin-3 levels | Weighted mode | 17 | 0.41 | 0.94 | 0.6660 | 1.51 | 0.24 | 9.57 |
| GCST90274830 | Osteoprotegerin levels | MR Egger | 18 | 0.87 | 0.67 | 0.2172 | 2.38 | 0.63 | 8.89 |
| GCST90274830 | Osteoprotegerin levels | Weighted median | 18 | 0.66 | 0.42 | 0.1161 | 1.93 | 0.85 | 4.37 |
| GCST90274830 | Osteoprotegerin levels | IVW | 18 | 0.70 | 0.32 | 0.0271 | 2.01 | 1.08 | 3.73 |
| GCST90274830 | Osteoprotegerin levels | Simple mode | 18 | 0.91 | 0.69 | 0.2032 | 2.48 | 0.65 | 9.56 |
| GCST90274830 | Osteoprotegerin levels | Weighted mode | 18 | 0.59 | 0.49 | 0.2444 | 1.80 | 0.69 | 4.67 |
| GCST90274831 | Oncostatin-M levels | MR Egger | 15 | -0.50 | 0.77 | 0.5300 | 0.61 | 0.14 | 2.75 |
| GCST90274831 | Oncostatin-M levels | Weighted median | 15 | 0.39 | 0.49 | 0.4198 | 1.48 | 0.57 | 3.84 |
| GCST90274831 | Oncostatin-M levels | IVW | 15 | 0.16 | 0.35 | 0.6599 | 1.17 | 0.59 | 2.33 |
| GCST90274831 | Oncostatin-M levels | Simple mode | 15 | 0.58 | 0.87 | 0.5190 | 1.78 | 0.32 | 9.87 |
| GCST90274831 | Oncostatin-M levels | Weighted mode | 15 | 0.56 | 0.77 | 0.4751 | 1.76 | 0.39 | 7.93 |
| GCST90274832 | Programmed cell death 1 ligand 1 levels | MR Egger | 16 | 0.22 | 0.90 | 0.8141 | 1.24 | 0.21 | 7.28 |
| GCST90274832 | Programmed cell death 1 ligand 1 levels | Weighted median | 16 | 0.64 | 0.50 | 0.2061 | 1.89 | 0.70 | 5.09 |
| GCST90274832 | Programmed cell death 1 ligand 1 levels | IVW | 16 | 0.51 | 0.37 | 0.1679 | 1.67 | 0.81 | 3.47 |
| GCST90274832 | Programmed cell death 1 ligand 1 levels | Simple mode | 16 | 0.72 | 0.87 | 0.4218 | 2.05 | 0.37 | 11.21 |
| GCST90274832 | Programmed cell death 1 ligand 1 levels | Weighted mode | 16 | 0.58 | 0.78 | 0.4688 | 1.79 | 0.38 | 8.34 |
| GCST90274833 | Stem cell factor levels | MR Egger | 32 | 0.25 | 0.37 | 0.5030 | 1.29 | 0.62 | 2.68 |
| GCST90274833 | Stem cell factor levels | Weighted median | 32 | 0.14 | 0.32 | 0.6603 | 1.15 | 0.61 | 2.17 |
| GCST90274833 | Stem cell factor levels | IVW | 32 | 0.29 | 0.21 | 0.1649 | 1.34 | 0.89 | 2.02 |
| GCST90274833 | Stem cell factor levels | Simple mode | 32 | 0.90 | 0.54 | 0.1097 | 2.45 | 0.84 | 7.14 |
| GCST90274833 | Stem cell factor levels | Weighted mode | 32 | 0.16 | 0.34 | 0.6460 | 1.17 | 0.60 | 2.26 |
| GCST90274834 | SIR2-like protein 2 levels | MR Egger | 13 | -1.42 | 0.82 | 0.1105 | 0.24 | 0.05 | 1.20 |
| GCST90274834 | SIR2-like protein 2 levels | Weighted median | 13 | -0.84 | 0.59 | 0.1535 | 0.43 | 0.14 | 1.37 |
| GCST90274834 | SIR2-like protein 2 levels | IVW | 13 | 0.02 | 0.41 | 0.9682 | 1.02 | 0.45 | 2.27 |
| GCST90274834 | SIR2-like protein 2 levels | Simple mode | 13 | -0.97 | 1.06 | 0.3804 | 0.38 | 0.05 | 3.05 |
| GCST90274834 | SIR2-like protein 2 levels | Weighted mode | 13 | -0.97 | 0.71 | 0.2007 | 0.38 | 0.09 | 1.54 |
| GCST90274835 | Signaling lymphocytic activation molecule levels | MR Egger | 25 | 0.27 | 0.61 | 0.6596 | 1.32 | 0.39 | 4.39 |
| GCST90274835 | Signaling lymphocytic activation molecule levels | Weighted median | 25 | -0.17 | 0.44 | 0.6958 | 0.84 | 0.36 | 1.99 |
| GCST90274835 | Signaling lymphocytic activation molecule levels | IVW | 25 | -0.32 | 0.29 | 0.2711 | 0.73 | 0.42 | 1.28 |
| GCST90274835 | Signaling lymphocytic activation molecule levels | Simple mode | 25 | 0.03 | 0.73 | 0.9727 | 1.03 | 0.25 | 4.28 |
| GCST90274835 | Signaling lymphocytic activation molecule levels | Weighted mode | 25 | -0.05 | 0.47 | 0.9216 | 0.95 | 0.38 | 2.38 |
| GCST90274836 | Sulfotransferase 1A1 levels | MR Egger | 23 | -0.04 | 0.63 | 0.9540 | 0.96 | 0.28 | 3.31 |
| GCST90274836 | Sulfotransferase 1A1 levels | Weighted median | 23 | 0.19 | 0.37 | 0.6133 | 1.21 | 0.58 | 2.49 |
| GCST90274836 | Sulfotransferase 1A1 levels | IVW | 23 | 0.30 | 0.26 | 0.2416 | 1.35 | 0.82 | 2.24 |
| GCST90274836 | Sulfotransferase 1A1 levels | Simple mode | 23 | -0.01 | 0.62 | 0.9841 | 0.99 | 0.29 | 3.36 |
| GCST90274836 | Sulfotransferase 1A1 levels | Weighted mode | 23 | 0.20 | 0.40 | 0.6272 | 1.22 | 0.55 | 2.69 |
| GCST90274837 | STAM binding protein levels | MR Egger | 11 | -2.82 | 1.60 | 0.1115 | 0.06 | 0.00 | 1.37 |
| GCST90274837 | STAM binding protein levels | Weighted median | 11 | 0.36 | 0.70 | 0.6112 | 1.43 | 0.36 | 5.69 |
| GCST90274837 | STAM binding protein levels | IVW | 11 | 0.10 | 0.56 | 0.8648 | 1.10 | 0.37 | 3.30 |
| GCST90274837 | STAM binding protein levels | Simple mode | 11 | -1.13 | 1.31 | 0.4095 | 0.32 | 0.02 | 4.23 |
| GCST90274837 | STAM binding protein levels | Weighted mode | 11 | 0.65 | 1.06 | 0.5525 | 1.92 | 0.24 | 15.29 |
| GCST90274838 | Transforming growth factor-alpha levels | MR Egger | 14 | 0.90 | 0.94 | 0.3582 | 2.46 | 0.39 | 15.65 |
| GCST90274838 | Transforming growth factor-alpha levels | Weighted median | 14 | 0.65 | 0.57 | 0.2574 | 1.91 | 0.62 | 5.85 |
| GCST90274838 | Transforming growth factor-alpha levels | IVW | 14 | 0.25 | 0.41 | 0.5367 | 1.29 | 0.58 | 2.86 |
| GCST90274838 | Transforming growth factor-alpha levels | Simple mode | 14 | 0.96 | 0.92 | 0.3176 | 2.60 | 0.43 | 15.78 |
| GCST90274838 | Transforming growth factor-alpha levels | Weighted mode | 14 | 0.79 | 1.04 | 0.4594 | 2.21 | 0.29 | 16.98 |
| GCST90274839 | Tumor necrosis factor levels | MR Egger | 18 | -0.26 | 0.65 | 0.6930 | 0.77 | 0.22 | 2.75 |
| GCST90274839 | Tumor necrosis factor levels | Weighted median | 18 | -0.47 | 0.51 | 0.3609 | 0.63 | 0.23 | 1.71 |
| GCST90274839 | Tumor necrosis factor levels | IVW | 18 | -0.07 | 0.35 | 0.8436 | 0.93 | 0.47 | 1.86 |
| GCST90274839 | Tumor necrosis factor levels | Simple mode | 18 | 0.62 | 0.86 | 0.4806 | 1.86 | 0.34 | 10.13 |
| GCST90274839 | Tumor necrosis factor levels | Weighted mode | 18 | -0.54 | 0.60 | 0.3825 | 0.58 | 0.18 | 1.89 |
| GCST90274840 | TNF-beta levels | MR Egger | 23 | -0.08 | 0.27 | 0.7823 | 0.93 | 0.54 | 1.58 |
| GCST90274840 | TNF-beta levels | Weighted median | 23 | -0.20 | 0.22 | 0.3573 | 0.82 | 0.53 | 1.25 |
| GCST90274840 | TNF-beta levels | IVW | 23 | -0.39 | 0.18 | 0.0282 | 0.68 | 0.48 | 0.96 |
| GCST90274840 | TNF-beta levels | Simple mode | 23 | -0.67 | 0.48 | 0.1720 | 0.51 | 0.20 | 1.30 |
| GCST90274840 | TNF-beta levels | Weighted mode | 23 | -0.19 | 0.22 | 0.3854 | 0.82 | 0.54 | 1.27 |
| GCST90274841 | Tumor necrosis factor receptor superfamily member 9 levels | MR Egger | 27 | -0.03 | 0.60 | 0.9601 | 0.97 | 0.30 | 3.12 |
| GCST90274841 | Tumor necrosis factor receptor superfamily member 9 levels | Weighted median | 27 | 0.14 | 0.37 | 0.6981 | 1.15 | 0.56 | 2.36 |
| GCST90274841 | Tumor necrosis factor receptor superfamily member 9 levels | IVW | 27 | 0.09 | 0.27 | 0.7224 | 1.10 | 0.65 | 1.85 |
| GCST90274841 | Tumor necrosis factor receptor superfamily member 9 levels | Simple mode | 27 | 0.01 | 0.66 | 0.9902 | 1.01 | 0.28 | 3.69 |
| GCST90274841 | Tumor necrosis factor receptor superfamily member 9 levels | Weighted mode | 27 | 0.11 | 0.51 | 0.8308 | 1.12 | 0.41 | 3.06 |
| GCST90274842 | Tumor necrosis factor ligand superfamily member 14 levels | MR Egger | 25 | 0.87 | 0.41 | 0.0443 | 2.39 | 1.07 | 5.33 |
| GCST90274842 | Tumor necrosis factor ligand superfamily member 14 levels | Weighted median | 25 | 0.37 | 0.38 | 0.3403 | 1.44 | 0.68 | 3.07 |
| GCST90274842 | Tumor necrosis factor ligand superfamily member 14 levels | IVW | 25 | 0.36 | 0.25 | 0.1516 | 1.43 | 0.88 | 2.35 |
| GCST90274842 | Tumor necrosis factor ligand superfamily member 14 levels | Simple mode | 25 | 0.72 | 0.72 | 0.3284 | 2.06 | 0.50 | 8.49 |
| GCST90274842 | Tumor necrosis factor ligand superfamily member 14 levels | Weighted mode | 25 | 0.53 | 0.40 | 0.1954 | 1.70 | 0.78 | 3.73 |
| GCST90274843 | TNF-related apoptosis-inducing ligand levels | MR Egger | 27 | 0.06 | 0.30 | 0.8523 | 1.06 | 0.59 | 1.91 |
| GCST90274843 | TNF-related apoptosis-inducing ligand levels | Weighted median | 27 | 0.06 | 0.28 | 0.8335 | 1.06 | 0.62 | 1.82 |
| GCST90274843 | TNF-related apoptosis-inducing ligand levels | IVW | 27 | -0.16 | 0.19 | 0.3840 | 0.85 | 0.59 | 1.23 |
| GCST90274843 | TNF-related apoptosis-inducing ligand levels | Simple mode | 27 | 0.21 | 0.45 | 0.6505 | 1.23 | 0.51 | 2.96 |
| GCST90274843 | TNF-related apoptosis-inducing ligand levels | Weighted mode | 27 | 0.02 | 0.28 | 0.9439 | 1.02 | 0.59 | 1.75 |
| GCST90274844 | TNF-related activation-induced cytokine levels | MR Egger | 28 | 0.26 | 0.45 | 0.5680 | 1.29 | 0.54 | 3.11 |
| GCST90274844 | TNF-related activation-induced cytokine levels | Weighted median | 28 | 0.06 | 0.35 | 0.8563 | 1.07 | 0.54 | 2.12 |
| GCST90274844 | TNF-related activation-induced cytokine levels | IVW | 28 | 0.04 | 0.23 | 0.8513 | 1.04 | 0.66 | 1.65 |
| GCST90274844 | TNF-related activation-induced cytokine levels | Simple mode | 28 | -0.17 | 0.60 | 0.7840 | 0.85 | 0.26 | 2.73 |
| GCST90274844 | TNF-related activation-induced cytokine levels | Weighted mode | 28 | 0.16 | 0.37 | 0.6621 | 1.18 | 0.57 | 2.42 |
| GCST90274845 | Thymic stromal lymphopoietin levels | MR Egger | 15 | 0.18 | 0.90 | 0.8469 | 1.19 | 0.20 | 6.96 |
| GCST90274845 | Thymic stromal lymphopoietin levels | Weighted median | 15 | 0.13 | 0.54 | 0.8126 | 1.14 | 0.39 | 3.29 |
| GCST90274845 | Thymic stromal lymphopoietin levels | IVW | 15 | 0.00 | 0.41 | 0.9972 | 1.00 | 0.45 | 2.22 |
| GCST90274845 | Thymic stromal lymphopoietin levels | Simple mode | 15 | 0.22 | 0.83 | 0.7988 | 1.24 | 0.24 | 6.28 |
| GCST90274845 | Thymic stromal lymphopoietin levels | Weighted mode | 15 | 0.25 | 0.83 | 0.7702 | 1.28 | 0.25 | 6.56 |
| GCST90274846 | Tumor necrosis factor ligand superfamily member 12 levels | MR Egger | 26 | 0.35 | 0.61 | 0.5737 | 1.41 | 0.43 | 4.66 |
| GCST90274846 | Tumor necrosis factor ligand superfamily member 12 levels | Weighted median | 26 | 0.42 | 0.42 | 0.3229 | 1.52 | 0.66 | 3.48 |
| GCST90274846 | Tumor necrosis factor ligand superfamily member 12 levels | IVW | 26 | 0.52 | 0.28 | 0.0609 | 1.68 | 0.98 | 2.88 |
| GCST90274846 | Tumor necrosis factor ligand superfamily member 12 levels | Simple mode | 26 | 0.43 | 0.70 | 0.5453 | 1.53 | 0.39 | 6.01 |
| GCST90274846 | Tumor necrosis factor ligand superfamily member 12 levels | Weighted mode | 26 | 0.31 | 0.43 | 0.4687 | 1.37 | 0.59 | 3.15 |
| GCST90274847 | Urokinase-type plasminogen activator levels | MR Egger | 21 | -0.05 | 0.63 | 0.9396 | 0.95 | 0.28 | 3.28 |
| GCST90274847 | Urokinase-type plasminogen activator levels | Weighted median | 21 | -0.17 | 0.41 | 0.6737 | 0.84 | 0.38 | 1.87 |
| GCST90274847 | Urokinase-type plasminogen activator levels | IVW | 21 | -0.22 | 0.29 | 0.4454 | 0.80 | 0.45 | 1.41 |
| GCST90274847 | Urokinase-type plasminogen activator levels | Simple mode | 21 | 0.45 | 0.70 | 0.5311 | 1.57 | 0.39 | 6.21 |
| GCST90274847 | Urokinase-type plasminogen activator levels | Weighted mode | 21 | 0.25 | 0.59 | 0.6790 | 1.28 | 0.40 | 4.06 |
| GCST90274848 | Vascular endothelial growth factor A levels | MR Egger | 24 | -0.11 | 0.29 | 0.7173 | 0.90 | 0.51 | 1.59 |
| GCST90274848 | Vascular endothelial growth factor A levels | Weighted median | 24 | -0.07 | 0.24 | 0.7814 | 0.94 | 0.58 | 1.50 |
| GCST90274848 | Vascular endothelial growth factor A levels | IVW | 24 | -0.01 | 0.19 | 0.9780 | 0.99 | 0.68 | 1.45 |
| GCST90274848 | Vascular endothelial growth factor A levels | Simple mode | 24 | 0.73 | 0.63 | 0.2614 | 2.07 | 0.60 | 7.14 |
| GCST90274848 | Vascular endothelial growth factor A levels | Weighted mode | 24 | -0.10 | 0.24 | 0.6897 | 0.91 | 0.57 | 1.45 |

SNP, single-nucleotide polymorphism; β, regression coefficient; SE, standard error; OR, odds ratio; Lo_CI, odds ratio lower 95% confidence interval; UP_CI, odds ratio upper 95% confidence interval.

**Supplement 5.** Reversed mendelian randomization analysis of precocious puberty on 91 inflammatory proteins.

| **ID** | **Outcome** | **Method** | **nSNP** | **β** | **SE** | **P-value** | **LO_CI** | **UP_CI** |
| --- | --- | --- | --- | --- | --- | --- | --- | --- |
| GCST90274758 | Eukaryotic translation initiation factor 4E-binding protein 1 levels | MR Egger | 4 | -0.05 | 0.07 | 0.5592 | -0.19 | 0.09 |
| GCST90274758 | Eukaryotic translation initiation factor 4E-binding protein 1 levels | Weighted median | 4 | 0.04 | 0.02 | 0.0660 | 0.00 | 0.08 |
| GCST90274758 | Eukaryotic translation initiation factor 4E-binding protein 1 levels | IVW | 4 | 0.02 | 0.02 | 0.4029 | -0.02 | 0.05 |
| GCST90274758 | Eukaryotic translation initiation factor 4E-binding protein 1 levels | Simple mode | 4 | 0.03 | 0.03 | 0.3617 | -0.03 | 0.10 |
| GCST90274758 | Eukaryotic translation initiation factor 4E-binding protein 1 levels | Weighted mode | 4 | 0.04 | 0.02 | 0.2242 | -0.01 | 0.08 |
| GCST90274759 | Adenosine Deaminase levels | MR Egger | 4 | -0.06 | 0.06 | 0.3616 | -0.17 | 0.04 |
| GCST90274759 | Adenosine Deaminase levels | Weighted median | 4 | 0.02 | 0.02 | 0.3702 | -0.02 | 0.06 |
| GCST90274759 | Adenosine Deaminase levels | IVW | 4 | 0.01 | 0.02 | 0.5058 | -0.02 | 0.04 |
| GCST90274759 | Adenosine Deaminase levels | Simple mode | 4 | 0.02 | 0.02 | 0.5040 | -0.03 | 0.06 |
| GCST90274759 | Adenosine Deaminase levels | Weighted mode | 4 | 0.02 | 0.02 | 0.4316 | -0.02 | 0.06 |
| GCST90274760 | Artemin levels | MR Egger | 4 | -0.08 | 0.06 | 0.3338 | -0.20 | 0.04 |
| GCST90274760 | Artemin levels | Weighted median | 4 | -0.01 | 0.02 | 0.5885 | -0.06 | 0.03 |
| GCST90274760 | Artemin levels | IVW | 4 | 0.00 | 0.02 | 0.9080 | -0.04 | 0.03 |
| GCST90274760 | Artemin levels | Simple mode | 4 | -0.01 | 0.03 | 0.6604 | -0.07 | 0.04 |
| GCST90274760 | Artemin levels | Weighted mode | 4 | -0.01 | 0.03 | 0.6392 | -0.06 | 0.04 |
| GCST90274761 | Axin-1 levels | MR Egger | 4 | 0.00 | 0.06 | 0.9968 | -0.12 | 0.12 |
| GCST90274761 | Axin-1 levels | Weighted median | 4 | 0.03 | 0.02 | 0.1109 | -0.01 | 0.08 |
| GCST90274761 | Axin-1 levels | IVW | 4 | 0.03 | 0.02 | 0.1105 | -0.01 | 0.06 |
| GCST90274761 | Axin-1 levels | Simple mode | 4 | 0.04 | 0.03 | 0.2927 | -0.02 | 0.09 |
| GCST90274761 | Axin-1 levels | Weighted mode | 4 | 0.04 | 0.03 | 0.2669 | -0.02 | 0.09 |
| GCST90274762 | beta-nerve growth factor levels | MR Egger | 4 | -0.01 | 0.05 | 0.8278 | -0.12 | 0.09 |
| GCST90274762 | beta-nerve growth factor levels | Weighted median | 4 | 0.03 | 0.02 | 0.0601 | 0.00 | 0.07 |
| GCST90274762 | beta-nerve growth factor levels | IVW | 4 | 0.03 | 0.01 | 0.0701 | 0.00 | 0.06 |
| GCST90274762 | beta-nerve growth factor levels | Simple mode | 4 | 0.03 | 0.02 | 0.2390 | -0.01 | 0.08 |
| GCST90274762 | beta-nerve growth factor levels | Weighted mode | 4 | 0.03 | 0.02 | 0.2066 | -0.01 | 0.08 |
| GCST90274763 | Caspase 8 levels | MR Egger | 4 | -0.06 | 0.06 | 0.4134 | -0.17 | 0.05 |
| GCST90274763 | Caspase 8 levels | Weighted median | 4 | 0.00 | 0.02 | 0.9123 | -0.04 | 0.04 |
| GCST90274763 | Caspase 8 levels | IVW | 4 | 0.01 | 0.02 | 0.6359 | -0.02 | 0.04 |
| GCST90274763 | Caspase 8 levels | Simple mode | 4 | -0.01 | 0.03 | 0.6333 | -0.07 | 0.04 |
| GCST90274763 | Caspase 8 levels | Weighted mode | 4 | 0.00 | 0.02 | 0.9501 | -0.05 | 0.04 |
| GCST90274764 | Eotaxin levels | MR Egger | 4 | -0.04 | 0.05 | 0.5558 | -0.15 | 0.07 |
| GCST90274764 | Eotaxin levels | Weighted median | 4 | -0.01 | 0.02 | 0.6387 | -0.04 | 0.03 |
| GCST90274764 | Eotaxin levels | IVW | 4 | 0.00 | 0.02 | 0.9312 | -0.03 | 0.03 |
| GCST90274764 | Eotaxin levels | Simple mode | 4 | -0.01 | 0.02 | 0.6757 | -0.06 | 0.04 |
| GCST90274764 | Eotaxin levels | Weighted mode | 4 | -0.01 | 0.02 | 0.7154 | -0.06 | 0.04 |
| GCST90274765 | C-C motif chemokine 19 levels | MR Egger | 4 | 0.05 | 0.06 | 0.4657 | -0.06 | 0.17 |
| GCST90274765 | C-C motif chemokine 19 levels | Weighted median | 4 | 0.01 | 0.02 | 0.6920 | -0.03 | 0.05 |
| GCST90274765 | C-C motif chemokine 19 levels | IVW | 4 | 0.01 | 0.02 | 0.6107 | -0.02 | 0.04 |
| GCST90274765 | C-C motif chemokine 19 levels | Simple mode | 4 | 0.01 | 0.03 | 0.8244 | -0.05 | 0.06 |
| GCST90274765 | C-C motif chemokine 19 levels | Weighted mode | 4 | 0.01 | 0.02 | 0.7352 | -0.04 | 0.05 |
| GCST90274766 | C-C motif chemokine 20 levels | MR Egger | 4 | -0.01 | 0.06 | 0.9045 | -0.12 | 0.10 |
| GCST90274766 | C-C motif chemokine 20 levels | Weighted median | 4 | 0.01 | 0.02 | 0.7555 | -0.03 | 0.04 |
| GCST90274766 | C-C motif chemokine 20 levels | IVW | 4 | 0.00 | 0.02 | 0.8394 | -0.03 | 0.03 |
| GCST90274766 | C-C motif chemokine 20 levels | Simple mode | 4 | 0.01 | 0.02 | 0.8163 | -0.04 | 0.05 |
| GCST90274766 | C-C motif chemokine 20 levels | Weighted mode | 4 | 0.01 | 0.02 | 0.7373 | -0.04 | 0.05 |
| GCST90274767 | C-C motif chemokine 23 levels | MR Egger | 4 | -0.03 | 0.06 | 0.6306 | -0.14 | 0.08 |
| GCST90274767 | C-C motif chemokine 23 levels | Weighted median | 4 | -0.01 | 0.02 | 0.7233 | -0.04 | 0.03 |
| GCST90274767 | C-C motif chemokine 23 levels | IVW | 4 | -0.01 | 0.02 | 0.7218 | -0.04 | 0.02 |
| GCST90274767 | C-C motif chemokine 23 levels | Simple mode | 4 | 0.00 | 0.02 | 0.8650 | -0.05 | 0.04 |
| GCST90274767 | C-C motif chemokine 23 levels | Weighted mode | 4 | -0.01 | 0.02 | 0.8314 | -0.05 | 0.04 |
| GCST90274768 | C-C motif chemokine 25 levels | MR Egger | 4 | 0.04 | 0.05 | 0.5027 | -0.06 | 0.15 |
| GCST90274768 | C-C motif chemokine 25 levels | Weighted median | 4 | 0.01 | 0.02 | 0.4192 | -0.02 | 0.05 |
| GCST90274768 | C-C motif chemokine 25 levels | IVW | 4 | 0.01 | 0.02 | 0.6362 | -0.02 | 0.04 |
| GCST90274768 | C-C motif chemokine 25 levels | Simple mode | 4 | 0.02 | 0.02 | 0.5547 | -0.03 | 0.06 |
| GCST90274768 | C-C motif chemokine 25 levels | Weighted mode | 4 | 0.01 | 0.02 | 0.5562 | -0.03 | 0.06 |
| GCST90274769 | C-C motif chemokine 28 levels | MR Egger | 4 | -0.03 | 0.05 | 0.6384 | -0.13 | 0.08 |
| GCST90274769 | C-C motif chemokine 28 levels | Weighted median | 4 | 0.01 | 0.02 | 0.5039 | -0.02 | 0.05 |
| GCST90274769 | C-C motif chemokine 28 levels | IVW | 4 | 0.01 | 0.01 | 0.3625 | -0.02 | 0.04 |
| GCST90274769 | C-C motif chemokine 28 levels | Simple mode | 4 | 0.00 | 0.02 | 0.9444 | -0.04 | 0.05 |
| GCST90274769 | C-C motif chemokine 28 levels | Weighted mode | 4 | 0.01 | 0.02 | 0.6971 | -0.03 | 0.05 |
| GCST90274770 | C-C motif chemokine 4 levels | MR Egger | 4 | 0.06 | 0.07 | 0.5081 | -0.08 | 0.20 |
| GCST90274770 | C-C motif chemokine 4 levels | Weighted median | 4 | 0.00 | 0.02 | 0.9536 | -0.04 | 0.04 |
| GCST90274770 | C-C motif chemokine 4 levels | IVW | 4 | 0.01 | 0.02 | 0.7347 | -0.03 | 0.04 |
| GCST90274770 | C-C motif chemokine 4 levels | Simple mode | 4 | 0.00 | 0.04 | 0.9628 | -0.07 | 0.07 |
| GCST90274770 | C-C motif chemokine 4 levels | Weighted mode | 4 | -0.01 | 0.04 | 0.7356 | -0.09 | 0.06 |
| GCST90274771 | Natural killer cell receptor 2B4 levels | MR Egger | 4 | -0.03 | 0.06 | 0.6041 | -0.14 | 0.08 |
| GCST90274771 | Natural killer cell receptor 2B4 levels | Weighted median | 4 | 0.01 | 0.02 | 0.4567 | -0.02 | 0.05 |
| GCST90274771 | Natural killer cell receptor 2B4 levels | IVW | 4 | 0.01 | 0.02 | 0.4242 | -0.02 | 0.04 |
| GCST90274771 | Natural killer cell receptor 2B4 levels | Simple mode | 4 | 0.00 | 0.02 | 0.9834 | -0.04 | 0.04 |
| GCST90274771 | Natural killer cell receptor 2B4 levels | Weighted mode | 4 | 0.02 | 0.02 | 0.4979 | -0.03 | 0.06 |
| GCST90274772 | CD40L receptor levels | MR Egger | 4 | -0.02 | 0.06 | 0.7798 | -0.15 | 0.11 |
| GCST90274772 | CD40L receptor levels | Weighted median | 4 | 0.01 | 0.02 | 0.5653 | -0.03 | 0.05 |
| GCST90274772 | CD40L receptor levels | IVW | 4 | 0.01 | 0.02 | 0.5824 | -0.02 | 0.04 |
| GCST90274772 | CD40L receptor levels | Simple mode | 4 | 0.01 | 0.03 | 0.6162 | -0.04 | 0.07 |
| GCST90274772 | CD40L receptor levels | Weighted mode | 4 | 0.02 | 0.02 | 0.5654 | -0.03 | 0.06 |
| GCST90274773 | T-cell surface glycoprotein CD5 levels | MR Egger | 4 | 0.00 | 0.06 | 0.9969 | -0.11 | 0.11 |
| GCST90274773 | T-cell surface glycoprotein CD5 levels | Weighted median | 4 | -0.01 | 0.02 | 0.6759 | -0.04 | 0.03 |
| GCST90274773 | T-cell surface glycoprotein CD5 levels | IVW | 4 | -0.01 | 0.02 | 0.4780 | -0.04 | 0.02 |
| GCST90274773 | T-cell surface glycoprotein CD5 levels | Simple mode | 4 | 0.00 | 0.02 | 0.9399 | -0.05 | 0.05 |
| GCST90274773 | T-cell surface glycoprotein CD5 levels | Weighted mode | 4 | 0.00 | 0.02 | 0.9437 | -0.05 | 0.04 |
| GCST90274774 | T-cell surface glycoprotein CD6 isoform levels | MR Egger | 4 | -0.06 | 0.06 | 0.4228 | -0.19 | 0.06 |
| GCST90274774 | T-cell surface glycoprotein CD6 isoform levels | Weighted median | 4 | -0.01 | 0.02 | 0.5075 | -0.05 | 0.03 |
| GCST90274774 | T-cell surface glycoprotein CD6 isoform levels | IVW | 4 | -0.02 | 0.02 | 0.3544 | -0.05 | 0.02 |
| GCST90274774 | T-cell surface glycoprotein CD6 isoform levels | Simple mode | 4 | 0.01 | 0.03 | 0.8156 | -0.06 | 0.07 |
| GCST90274774 | T-cell surface glycoprotein CD6 isoform levels | Weighted mode | 4 | 0.01 | 0.03 | 0.7524 | -0.05 | 0.07 |
| GCST90274775 | CUB domain-containing protein 1 levels | MR Egger | 4 | -0.02 | 0.05 | 0.7796 | -0.12 | 0.09 |
| GCST90274775 | CUB domain-containing protein 1 levels | Weighted median | 4 | 0.01 | 0.02 | 0.4471 | -0.02 | 0.05 |
| GCST90274775 | CUB domain-containing protein 1 levels | IVW | 4 | 0.01 | 0.01 | 0.3697 | -0.02 | 0.04 |
| GCST90274775 | CUB domain-containing protein 1 levels | Simple mode | 4 | 0.02 | 0.02 | 0.5274 | -0.03 | 0.07 |
| GCST90274775 | CUB domain-containing protein 1 levels | Weighted mode | 4 | 0.01 | 0.02 | 0.5332 | -0.03 | 0.05 |
| GCST90274776 | Macrophage colony-stimulating factor 1 levels | MR Egger | 4 | -0.03 | 0.06 | 0.6324 | -0.14 | 0.08 |
| GCST90274776 | Macrophage colony-stimulating factor 1 levels | Weighted median | 4 | 0.01 | 0.02 | 0.7498 | -0.03 | 0.04 |
| GCST90274776 | Macrophage colony-stimulating factor 1 levels | IVW | 4 | 0.01 | 0.02 | 0.7087 | -0.02 | 0.04 |
| GCST90274776 | Macrophage colony-stimulating factor 1 levels | Simple mode | 4 | 0.01 | 0.02 | 0.7951 | -0.04 | 0.05 |
| GCST90274776 | Macrophage colony-stimulating factor 1 levels | Weighted mode | 4 | 0.01 | 0.02 | 0.7674 | -0.04 | 0.05 |
| GCST90274777 | Cystatin D levels | MR Egger | 4 | 0.00 | 0.05 | 0.9541 | -0.10 | 0.11 |
| GCST90274777 | Cystatin D levels | Weighted median | 4 | -0.01 | 0.02 | 0.5516 | -0.05 | 0.02 |
| GCST90274777 | Cystatin D levels | IVW | 4 | -0.01 | 0.02 | 0.5464 | -0.04 | 0.02 |
| GCST90274777 | Cystatin D levels | Simple mode | 4 | -0.01 | 0.02 | 0.5582 | -0.06 | 0.03 |
| GCST90274777 | Cystatin D levels | Weighted mode | 4 | -0.01 | 0.02 | 0.5973 | -0.05 | 0.03 |
| GCST90274778 | Fractalkine levels | MR Egger | 4 | 0.05 | 0.06 | 0.4998 | -0.06 | 0.15 |
| GCST90274778 | Fractalkine levels | Weighted median | 4 | 0.01 | 0.02 | 0.5843 | -0.03 | 0.05 |
| GCST90274778 | Fractalkine levels | IVW | 4 | 0.00 | 0.02 | 0.7787 | -0.03 | 0.03 |
| GCST90274778 | Fractalkine levels | Simple mode | 4 | 0.02 | 0.03 | 0.5037 | -0.03 | 0.07 |
| GCST90274778 | Fractalkine levels | Weighted mode | 4 | 0.02 | 0.03 | 0.4644 | -0.03 | 0.07 |
| GCST90274779 | C-X-C motif chemokine 1 levels | MR Egger | 4 | 0.08 | 0.06 | 0.2945 | -0.03 | 0.19 |
| GCST90274779 | C-X-C motif chemokine 1 levels | Weighted median | 4 | 0.04 | 0.02 | 0.0589 | 0.00 | 0.08 |
| GCST90274779 | C-X-C motif chemokine 1 levels | IVW | 4 | 0.03 | 0.02 | 0.0370 | 0.00 | 0.06 |
| GCST90274779 | C-X-C motif chemokine 1 levels | Simple mode | 4 | 0.04 | 0.03 | 0.2121 | -0.01 | 0.10 |
| GCST90274779 | C-X-C motif chemokine 1 levels | Weighted mode | 4 | 0.04 | 0.03 | 0.1821 | -0.01 | 0.09 |
| GCST90274780 | C-X-C motif chemokine 10 levels | MR Egger | 4 | 0.01 | 0.06 | 0.9350 | -0.10 | 0.11 |
| GCST90274780 | C-X-C motif chemokine 10 levels | Weighted median | 4 | -0.01 | 0.02 | 0.7164 | -0.04 | 0.03 |
| GCST90274780 | C-X-C motif chemokine 10 levels | IVW | 4 | 0.00 | 0.02 | 0.7843 | -0.03 | 0.03 |
| GCST90274780 | C-X-C motif chemokine 10 levels | Simple mode | 4 | -0.01 | 0.02 | 0.7552 | -0.05 | 0.04 |
| GCST90274780 | C-X-C motif chemokine 10 levels | Weighted mode | 4 | -0.01 | 0.02 | 0.6889 | -0.05 | 0.03 |
| GCST90274781 | C-X-C motif chemokine 11 levels | MR Egger | 4 | 0.06 | 0.06 | 0.4607 | -0.07 | 0.18 |
| GCST90274781 | C-X-C motif chemokine 11 levels | Weighted median | 4 | 0.01 | 0.02 | 0.6255 | -0.03 | 0.05 |
| GCST90274781 | C-X-C motif chemokine 11 levels | IVW | 4 | 0.02 | 0.02 | 0.1326 | -0.01 | 0.05 |
| GCST90274781 | C-X-C motif chemokine 11 levels | Simple mode | 4 | 0.00 | 0.03 | 0.9216 | -0.06 | 0.06 |
| GCST90274781 | C-X-C motif chemokine 11 levels | Weighted mode | 4 | 0.00 | 0.03 | 0.9819 | -0.06 | 0.06 |
| GCST90274782 | C-X-C motif chemokine 5 levels | MR Egger | 4 | 0.08 | 0.06 | 0.2971 | -0.03 | 0.19 |
| GCST90274782 | C-X-C motif chemokine 5 levels | Weighted median | 4 | 0.03 | 0.02 | 0.0986 | -0.01 | 0.07 |
| GCST90274782 | C-X-C motif chemokine 5 levels | IVW | 4 | 0.02 | 0.02 | 0.2509 | -0.01 | 0.05 |
| GCST90274782 | C-X-C motif chemokine 5 levels | Simple mode | 4 | 0.03 | 0.03 | 0.3224 | -0.02 | 0.09 |
| GCST90274782 | C-X-C motif chemokine 5 levels | Weighted mode | 4 | 0.03 | 0.03 | 0.2939 | -0.02 | 0.08 |
| GCST90274783 | C-X-C motif chemokine 6 levels | MR Egger | 4 | 0.03 | 0.06 | 0.6052 | -0.08 | 0.15 |
| GCST90274783 | C-X-C motif chemokine 6 levels | Weighted median | 4 | 0.01 | 0.02 | 0.4678 | -0.02 | 0.05 |
| GCST90274783 | C-X-C motif chemokine 6 levels | IVW | 4 | 0.02 | 0.02 | 0.2511 | -0.01 | 0.05 |
| GCST90274783 | C-X-C motif chemokine 6 levels | Simple mode | 4 | 0.00 | 0.03 | 0.9330 | -0.06 | 0.05 |
| GCST90274783 | C-X-C motif chemokine 6 levels | Weighted mode | 4 | 0.00 | 0.03 | 0.9032 | -0.06 | 0.05 |
| GCST90274784 | C-X-C motif chemokine 9 levels | MR Egger | 4 | 0.00 | 0.05 | 0.9532 | -0.10 | 0.11 |
| GCST90274784 | C-X-C motif chemokine 9 levels | Weighted median | 4 | 0.00 | 0.02 | 0.9184 | -0.03 | 0.04 |
| GCST90274784 | C-X-C motif chemokine 9 levels | IVW | 4 | 0.01 | 0.01 | 0.6609 | -0.02 | 0.04 |
| GCST90274784 | C-X-C motif chemokine 9 levels | Simple mode | 4 | 0.00 | 0.02 | 0.9637 | -0.04 | 0.04 |
| GCST90274784 | C-X-C motif chemokine 9 levels | Weighted mode | 4 | 0.00 | 0.02 | 0.9980 | -0.05 | 0.05 |
| GCST90274785 | Delta and Notch-like epidermal growth factor-related receptor levels | MR Egger | 4 | -0.05 | 0.12 | 0.7296 | -0.27 | 0.18 |
| GCST90274785 | Delta and Notch-like epidermal growth factor-related receptor levels | Weighted median | 4 | 0.01 | 0.02 | 0.6542 | -0.03 | 0.05 |
| GCST90274785 | Delta and Notch-like epidermal growth factor-related receptor levels | IVW | 4 | 0.01 | 0.03 | 0.7473 | -0.04 | 0.06 |
| GCST90274785 | Delta and Notch-like epidermal growth factor-related receptor levels | Simple mode | 4 | 0.02 | 0.03 | 0.6153 | -0.04 | 0.07 |
| GCST90274785 | Delta and Notch-like epidermal growth factor-related receptor levels | Weighted mode | 4 | 0.01 | 0.03 | 0.6360 | -0.04 | 0.06 |
| GCST90274786 | Protein S100-A12 levels | MR Egger | 4 | -0.03 | 0.06 | 0.6614 | -0.14 | 0.08 |
| GCST90274786 | Protein S100-A12 levels | Weighted median | 4 | 0.02 | 0.02 | 0.2785 | -0.02 | 0.06 |
| GCST90274786 | Protein S100-A12 levels | IVW | 4 | 0.01 | 0.02 | 0.3861 | -0.02 | 0.04 |
| GCST90274786 | Protein S100-A12 levels | Simple mode | 4 | 0.02 | 0.02 | 0.4376 | -0.02 | 0.06 |
| GCST90274786 | Protein S100-A12 levels | Weighted mode | 4 | 0.02 | 0.02 | 0.4150 | -0.02 | 0.07 |
| GCST90274787 | Fibroblast growth factor 19 levels | MR Egger | 4 | 0.09 | 0.07 | 0.3056 | -0.04 | 0.22 |
| GCST90274787 | Fibroblast growth factor 19 levels | Weighted median | 4 | 0.03 | 0.02 | 0.1380 | -0.01 | 0.07 |
| GCST90274787 | Fibroblast growth factor 19 levels | IVW | 4 | 0.04 | 0.02 | 0.0300 | 0.00 | 0.07 |
| GCST90274787 | Fibroblast growth factor 19 levels | Simple mode | 4 | 0.03 | 0.03 | 0.3731 | -0.03 | 0.10 |
| GCST90274787 | Fibroblast growth factor 19 levels | Weighted mode | 4 | 0.02 | 0.03 | 0.6524 | -0.05 | 0.08 |
| GCST90274788 | Fibroblast growth factor 21 levels | MR Egger | 4 | -0.10 | 0.06 | 0.2152 | -0.21 | 0.01 |
| GCST90274788 | Fibroblast growth factor 21 levels | Weighted median | 4 | -0.01 | 0.02 | 0.6050 | -0.05 | 0.03 |
| GCST90274788 | Fibroblast growth factor 21 levels | IVW | 4 | 0.00 | 0.02 | 0.9601 | -0.04 | 0.04 |
| GCST90274788 | Fibroblast growth factor 21 levels | Simple mode | 4 | -0.02 | 0.03 | 0.4980 | -0.09 | 0.04 |
| GCST90274788 | Fibroblast growth factor 21 levels | Weighted mode | 4 | -0.02 | 0.03 | 0.4862 | -0.08 | 0.03 |
| GCST90274789 | Fibroblast growth factor 23 levels | MR Egger | 4 | -0.02 | 0.07 | 0.8157 | -0.15 | 0.12 |
| GCST90274789 | Fibroblast growth factor 23 levels | Weighted median | 4 | -0.01 | 0.02 | 0.6868 | -0.05 | 0.03 |
| GCST90274789 | Fibroblast growth factor 23 levels | IVW | 4 | 0.00 | 0.02 | 0.8074 | -0.03 | 0.03 |
| GCST90274789 | Fibroblast growth factor 23 levels | Simple mode | 4 | -0.02 | 0.03 | 0.5988 | -0.07 | 0.04 |
| GCST90274789 | Fibroblast growth factor 23 levels | Weighted mode | 4 | -0.01 | 0.03 | 0.6715 | -0.06 | 0.04 |
| GCST90274790 | Fibroblast growth factor 5 levels | MR Egger | 4 | -0.03 | 0.06 | 0.6759 | -0.15 | 0.09 |
| GCST90274790 | Fibroblast growth factor 5 levels | Weighted median | 4 | 0.04 | 0.02 | 0.0389 | 0.00 | 0.08 |
| GCST90274790 | Fibroblast growth factor 5 levels | IVW | 4 | 0.04 | 0.02 | 0.0091 | 0.01 | 0.08 |
| GCST90274790 | Fibroblast growth factor 5 levels | Simple mode | 4 | 0.05 | 0.03 | 0.2030 | -0.01 | 0.11 |
| GCST90274790 | Fibroblast growth factor 5 levels | Weighted mode | 4 | 0.05 | 0.03 | 0.1995 | -0.01 | 0.10 |
| GCST90274791 | Fms-related tyrosine kinase 3 ligand levels | MR Egger | 4 | 0.02 | 0.06 | 0.7219 | -0.09 | 0.13 |
| GCST90274791 | Fms-related tyrosine kinase 3 ligand levels | Weighted median | 4 | -0.02 | 0.02 | 0.4011 | -0.05 | 0.02 |
| GCST90274791 | Fms-related tyrosine kinase 3 ligand levels | IVW | 4 | -0.02 | 0.02 | 0.3068 | -0.05 | 0.01 |
| GCST90274791 | Fms-related tyrosine kinase 3 ligand levels | Simple mode | 4 | -0.03 | 0.03 | 0.4185 | -0.09 | 0.03 |
| GCST90274791 | Fms-related tyrosine kinase 3 ligand levels | Weighted mode | 4 | -0.03 | 0.03 | 0.3392 | -0.09 | 0.02 |
| GCST90274792 | Glial cell line-derived neurotrophic factor levels | MR Egger | 4 | -0.06 | 0.05 | 0.4074 | -0.16 | 0.05 |
| GCST90274792 | Glial cell line-derived neurotrophic factor levels | Weighted median | 4 | 0.00 | 0.02 | 0.8020 | -0.04 | 0.03 |
| GCST90274792 | Glial cell line-derived neurotrophic factor levels | IVW | 4 | -0.01 | 0.02 | 0.5307 | -0.04 | 0.02 |
| GCST90274792 | Glial cell line-derived neurotrophic factor levels | Simple mode | 4 | -0.03 | 0.03 | 0.3579 | -0.09 | 0.02 |
| GCST90274792 | Glial cell line-derived neurotrophic factor levels | Weighted mode | 4 | 0.00 | 0.02 | 0.9611 | -0.04 | 0.04 |
| GCST90274793 | Hepatocyte growth factor levels | MR Egger | 4 | -0.11 | 0.06 | 0.1883 | -0.22 | 0.00 |
| GCST90274793 | Hepatocyte growth factor levels | Weighted median | 4 | 0.00 | 0.02 | 0.9016 | -0.04 | 0.04 |
| GCST90274793 | Hepatocyte growth factor levels | IVW | 4 | 0.00 | 0.02 | 0.8229 | -0.04 | 0.03 |
| GCST90274793 | Hepatocyte growth factor levels | Simple mode | 4 | -0.01 | 0.03 | 0.7198 | -0.07 | 0.04 |
| GCST90274793 | Hepatocyte growth factor levels | Weighted mode | 4 | 0.00 | 0.02 | 0.9696 | -0.04 | 0.05 |
| GCST90274794 | Interferon gamma levels | MR Egger | 4 | 0.05 | 0.06 | 0.5112 | -0.07 | 0.17 |
| GCST90274794 | Interferon gamma levels | Weighted median | 4 | 0.02 | 0.02 | 0.4070 | -0.02 | 0.06 |
| GCST90274794 | Interferon gamma levels | IVW | 4 | 0.02 | 0.02 | 0.3546 | -0.02 | 0.05 |
| GCST90274794 | Interferon gamma levels | Simple mode | 4 | 0.02 | 0.03 | 0.5662 | -0.04 | 0.08 |
| GCST90274794 | Interferon gamma levels | Weighted mode | 4 | 0.02 | 0.02 | 0.5269 | -0.03 | 0.06 |
| GCST90274795 | Interleukin-10 levels | MR Egger | 4 | 0.03 | 0.06 | 0.6155 | -0.08 | 0.14 |
| GCST90274795 | Interleukin-10 levels | Weighted median | 4 | 0.00 | 0.02 | 0.9484 | -0.03 | 0.04 |
| GCST90274795 | Interleukin-10 levels | IVW | 4 | 0.00 | 0.02 | 0.8706 | -0.03 | 0.03 |
| GCST90274795 | Interleukin-10 levels | Simple mode | 4 | 0.01 | 0.02 | 0.8069 | -0.04 | 0.05 |
| GCST90274795 | Interleukin-10 levels | Weighted mode | 4 | 0.01 | 0.03 | 0.7252 | -0.04 | 0.06 |
| GCST90274796 | Interleukin-10 receptor subunit alpha levels | MR Egger | 4 | -0.02 | 0.06 | 0.7397 | -0.15 | 0.10 |
| GCST90274796 | Interleukin-10 receptor subunit alpha levels | Weighted median | 4 | -0.02 | 0.02 | 0.3666 | -0.06 | 0.02 |
| GCST90274796 | Interleukin-10 receptor subunit alpha levels | IVW | 4 | -0.02 | 0.02 | 0.3315 | -0.05 | 0.02 |
| GCST90274796 | Interleukin-10 receptor subunit alpha levels | Simple mode | 4 | 0.00 | 0.03 | 0.8767 | -0.05 | 0.05 |
| GCST90274796 | Interleukin-10 receptor subunit alpha levels | Weighted mode | 4 | -0.03 | 0.03 | 0.3866 | -0.07 | 0.02 |
| GCST90274797 | Interleukin-10 receptor subunit beta levels | MR Egger | 4 | -0.06 | 0.06 | 0.3778 | -0.17 | 0.05 |
| GCST90274797 | Interleukin-10 receptor subunit beta levels | Weighted median | 4 | 0.00 | 0.02 | 0.8530 | -0.04 | 0.03 |
| GCST90274797 | Interleukin-10 receptor subunit beta levels | IVW | 4 | 0.00 | 0.02 | 0.7841 | -0.03 | 0.03 |
| GCST90274797 | Interleukin-10 receptor subunit beta levels | Simple mode | 4 | 0.00 | 0.02 | 0.9596 | -0.05 | 0.05 |
| GCST90274797 | Interleukin-10 receptor subunit beta levels | Weighted mode | 4 | 0.00 | 0.02 | 0.9362 | -0.04 | 0.04 |
| GCST90274798 | Interleukin-12 subunit beta levels | MR Egger | 4 | 0.00 | 0.06 | 0.9373 | -0.11 | 0.10 |
| GCST90274798 | Interleukin-12 subunit beta levels | Weighted median | 4 | 0.00 | 0.02 | 0.8346 | -0.04 | 0.03 |
| GCST90274798 | Interleukin-12 subunit beta levels | IVW | 4 | 0.00 | 0.02 | 0.8566 | -0.03 | 0.03 |
| GCST90274798 | Interleukin-12 subunit beta levels | Simple mode | 4 | -0.01 | 0.02 | 0.8111 | -0.05 | 0.04 |
| GCST90274798 | Interleukin-12 subunit beta levels | Weighted mode | 4 | 0.00 | 0.02 | 0.8489 | -0.05 | 0.04 |
| GCST90274799 | Interleukin-13 levels | MR Egger | 4 | -0.05 | 0.06 | 0.5156 | -0.17 | 0.07 |
| GCST90274799 | Interleukin-13 levels | Weighted median | 4 | -0.01 | 0.02 | 0.7205 | -0.05 | 0.03 |
| GCST90274799 | Interleukin-13 levels | IVW | 4 | 0.00 | 0.02 | 0.7744 | -0.03 | 0.04 |
| GCST90274799 | Interleukin-13 levels | Simple mode | 4 | -0.01 | 0.03 | 0.7862 | -0.07 | 0.05 |
| GCST90274799 | Interleukin-13 levels | Weighted mode | 4 | -0.01 | 0.03 | 0.7086 | -0.06 | 0.04 |
| GCST90274800 | Interleukin-15 receptor subunit alpha levels | MR Egger | 4 | -0.04 | 0.06 | 0.6062 | -0.16 | 0.08 |
| GCST90274800 | Interleukin-15 receptor subunit alpha levels | Weighted median | 4 | 0.05 | 0.02 | 0.0223 | 0.01 | 0.09 |
| GCST90274800 | Interleukin-15 receptor subunit alpha levels | IVW | 4 | 0.04 | 0.02 | 0.0191 | 0.01 | 0.08 |
| GCST90274800 | Interleukin-15 receptor subunit alpha levels | Simple mode | 4 | 0.06 | 0.04 | 0.2011 | -0.01 | 0.13 |
| GCST90274800 | Interleukin-15 receptor subunit alpha levels | Weighted mode | 4 | 0.06 | 0.03 | 0.1091 | 0.01 | 0.12 |
| GCST90274801 | Interleukin-17A levels | MR Egger | 4 | -0.01 | 0.06 | 0.8775 | -0.13 | 0.11 |
| GCST90274801 | Interleukin-17A levels | Weighted median | 4 | 0.00 | 0.02 | 0.9752 | -0.04 | 0.04 |
| GCST90274801 | Interleukin-17A levels | IVW | 4 | 0.00 | 0.02 | 0.9995 | -0.03 | 0.03 |
| GCST90274801 | Interleukin-17A levels | Simple mode | 4 | 0.00 | 0.02 | 0.9340 | -0.05 | 0.05 |
| GCST90274801 | Interleukin-17A levels | Weighted mode | 4 | 0.00 | 0.02 | 0.9618 | -0.04 | 0.05 |
| GCST90274802 | Interleukin-17C levels | MR Egger | 4 | -0.03 | 0.06 | 0.7021 | -0.15 | 0.09 |
| GCST90274802 | Interleukin-17C levels | Weighted median | 4 | 0.01 | 0.02 | 0.6287 | -0.03 | 0.05 |
| GCST90274802 | Interleukin-17C levels | IVW | 4 | 0.01 | 0.02 | 0.6043 | -0.02 | 0.04 |
| GCST90274802 | Interleukin-17C levels | Simple mode | 4 | 0.01 | 0.02 | 0.7003 | -0.04 | 0.06 |
| GCST90274802 | Interleukin-17C levels | Weighted mode | 4 | 0.01 | 0.02 | 0.6717 | -0.04 | 0.06 |
| GCST90274803 | Interleukin-18 levels | MR Egger | 4 | -0.11 | 0.06 | 0.1840 | -0.22 | 0.00 |
| GCST90274803 | Interleukin-18 levels | Weighted median | 4 | 0.00 | 0.02 | 0.9542 | -0.04 | 0.04 |
| GCST90274803 | Interleukin-18 levels | IVW | 4 | 0.00 | 0.02 | 0.9932 | -0.04 | 0.04 |
| GCST90274803 | Interleukin-18 levels | Simple mode | 4 | 0.00 | 0.03 | 0.8620 | -0.04 | 0.05 |
| GCST90274803 | Interleukin-18 levels | Weighted mode | 4 | 0.00 | 0.02 | 0.8663 | -0.04 | 0.05 |
| GCST90274804 | interleukin-18 receptor 1 levels | MR Egger | 4 | 0.03 | 0.06 | 0.6564 | -0.08 | 0.14 |
| GCST90274804 | interleukin-18 receptor 1 levels | Weighted median | 4 | 0.02 | 0.02 | 0.4151 | -0.02 | 0.05 |
| GCST90274804 | interleukin-18 receptor 1 levels | IVW | 4 | 0.01 | 0.02 | 0.3486 | -0.02 | 0.04 |
| GCST90274804 | interleukin-18 receptor 1 levels | Simple mode | 4 | 0.00 | 0.02 | 0.9200 | -0.04 | 0.05 |
| GCST90274804 | interleukin-18 receptor 1 levels | Weighted mode | 4 | 0.03 | 0.02 | 0.3456 | -0.02 | 0.07 |
| GCST90274805 | Interleukin-1-alpha levels | MR Egger | 4 | -0.02 | 0.06 | 0.7503 | -0.14 | 0.10 |
| GCST90274805 | Interleukin-1-alpha levels | Weighted median | 4 | -0.02 | 0.02 | 0.2594 | -0.07 | 0.02 |
| GCST90274805 | Interleukin-1-alpha levels | IVW | 4 | -0.03 | 0.02 | 0.1267 | -0.06 | 0.01 |
| GCST90274805 | Interleukin-1-alpha levels | Simple mode | 4 | -0.02 | 0.03 | 0.4582 | -0.07 | 0.03 |
| GCST90274805 | Interleukin-1-alpha levels | Weighted mode | 4 | -0.02 | 0.03 | 0.4347 | -0.07 | 0.03 |
| GCST90274806 | Interleukin-2 levels | MR Egger | 4 | -0.03 | 0.10 | 0.7754 | -0.24 | 0.17 |
| GCST90274806 | Interleukin-2 levels | Weighted median | 4 | 0.01 | 0.02 | 0.5781 | -0.03 | 0.06 |
| GCST90274806 | Interleukin-2 levels | IVW | 4 | 0.01 | 0.02 | 0.5889 | -0.04 | 0.06 |
| GCST90274806 | Interleukin-2 levels | Simple mode | 4 | 0.05 | 0.04 | 0.3142 | -0.03 | 0.13 |
| GCST90274806 | Interleukin-2 levels | Weighted mode | 4 | -0.02 | 0.03 | 0.6249 | -0.08 | 0.04 |
| GCST90274807 | Interleukin-20 levels | MR Egger | 4 | -0.05 | 0.06 | 0.4901 | -0.17 | 0.07 |
| GCST90274807 | Interleukin-20 levels | Weighted median | 4 | 0.02 | 0.02 | 0.3239 | -0.02 | 0.06 |
| GCST90274807 | Interleukin-20 levels | IVW | 4 | 0.01 | 0.02 | 0.4271 | -0.02 | 0.05 |
| GCST90274807 | Interleukin-20 levels | Simple mode | 4 | 0.02 | 0.03 | 0.5249 | -0.04 | 0.08 |
| GCST90274807 | Interleukin-20 levels | Weighted mode | 4 | 0.02 | 0.02 | 0.3845 | -0.02 | 0.07 |
| GCST90274808 | Interleukin-20 receptor subunit alpha levels | MR Egger | 4 | -0.13 | 0.06 | 0.1799 | -0.25 | 0.00 |
| GCST90274808 | Interleukin-20 receptor subunit alpha levels | Weighted median | 4 | -0.02 | 0.02 | 0.3659 | -0.07 | 0.02 |
| GCST90274808 | Interleukin-20 receptor subunit alpha levels | IVW | 4 | -0.02 | 0.02 | 0.2786 | -0.06 | 0.02 |
| GCST90274808 | Interleukin-20 receptor subunit alpha levels | Simple mode | 4 | -0.05 | 0.03 | 0.2580 | -0.11 | 0.02 |
| GCST90274808 | Interleukin-20 receptor subunit alpha levels | Weighted mode | 4 | -0.02 | 0.03 | 0.4785 | -0.08 | 0.03 |
| GCST90274809 | Interleukin-22 receptor subunit alpha-1 levels | MR Egger | 4 | -0.02 | 0.06 | 0.7994 | -0.14 | 0.10 |
| GCST90274809 | Interleukin-22 receptor subunit alpha-1 levels | Weighted median | 4 | -0.03 | 0.02 | 0.1794 | -0.07 | 0.01 |
| GCST90274809 | Interleukin-22 receptor subunit alpha-1 levels | IVW | 4 | -0.02 | 0.02 | 0.1851 | -0.06 | 0.01 |
| GCST90274809 | Interleukin-22 receptor subunit alpha-1 levels | Simple mode | 4 | -0.04 | 0.03 | 0.2830 | -0.11 | 0.02 |
| GCST90274809 | Interleukin-22 receptor subunit alpha-1 levels | Weighted mode | 4 | -0.04 | 0.03 | 0.2406 | -0.10 | 0.01 |
| GCST90274810 | Interleukin-24 levels | MR Egger | 4 | -0.08 | 0.06 | 0.3417 | -0.20 | 0.05 |
| GCST90274810 | Interleukin-24 levels | Weighted median | 4 | 0.01 | 0.02 | 0.7061 | -0.03 | 0.05 |
| GCST90274810 | Interleukin-24 levels | IVW | 4 | 0.00 | 0.02 | 0.8557 | -0.04 | 0.03 |
| GCST90274810 | Interleukin-24 levels | Simple mode | 4 | 0.01 | 0.03 | 0.7999 | -0.05 | 0.06 |
| GCST90274810 | Interleukin-24 levels | Weighted mode | 4 | 0.01 | 0.02 | 0.7159 | -0.04 | 0.06 |
| GCST90274811 | Interleukin-2 receptor subunit beta levels | MR Egger | 4 | -0.02 | 0.08 | 0.8106 | -0.18 | 0.13 |
| GCST90274811 | Interleukin-2 receptor subunit beta levels | Weighted median | 4 | 0.02 | 0.02 | 0.2856 | -0.02 | 0.07 |
| GCST90274811 | Interleukin-2 receptor subunit beta levels | IVW | 4 | 0.02 | 0.02 | 0.3896 | -0.02 | 0.05 |
| GCST90274811 | Interleukin-2 receptor subunit beta levels | Simple mode | 4 | 0.03 | 0.03 | 0.3496 | -0.03 | 0.09 |
| GCST90274811 | Interleukin-2 receptor subunit beta levels | Weighted mode | 4 | 0.03 | 0.02 | 0.3111 | -0.02 | 0.08 |
| GCST90274812 | Interleukin-33 levels | MR Egger | 4 | -0.05 | 0.06 | 0.4818 | -0.17 | 0.07 |
| GCST90274812 | Interleukin-33 levels | Weighted median | 4 | -0.01 | 0.02 | 0.6982 | -0.05 | 0.04 |
| GCST90274812 | Interleukin-33 levels | IVW | 4 | -0.01 | 0.02 | 0.4777 | -0.05 | 0.02 |
| GCST90274812 | Interleukin-33 levels | Simple mode | 4 | -0.04 | 0.03 | 0.2687 | -0.10 | 0.02 |
| GCST90274812 | Interleukin-33 levels | Weighted mode | 4 | 0.00 | 0.03 | 0.9793 | -0.05 | 0.05 |
| GCST90274813 | Interleukin-4 levels | MR Egger | 4 | -0.09 | 0.06 | 0.3015 | -0.21 | 0.04 |
| GCST90274813 | Interleukin-4 levels | Weighted median | 4 | -0.01 | 0.02 | 0.6702 | -0.05 | 0.03 |
| GCST90274813 | Interleukin-4 levels | IVW | 4 | 0.00 | 0.02 | 0.9532 | -0.03 | 0.03 |
| GCST90274813 | Interleukin-4 levels | Simple mode | 4 | -0.03 | 0.03 | 0.4850 | -0.09 | 0.04 |
| GCST90274813 | Interleukin-4 levels | Weighted mode | 4 | -0.02 | 0.03 | 0.6330 | -0.07 | 0.04 |
| GCST90274814 | Interleukin-5 levels | MR Egger | 4 | -0.01 | 0.06 | 0.9198 | -0.13 | 0.12 |
| GCST90274814 | Interleukin-5 levels | Weighted median | 4 | 0.00 | 0.02 | 0.9866 | -0.04 | 0.04 |
| GCST90274814 | Interleukin-5 levels | IVW | 4 | 0.00 | 0.02 | 0.8411 | -0.04 | 0.03 |
| GCST90274814 | Interleukin-5 levels | Simple mode | 4 | 0.01 | 0.03 | 0.6918 | -0.04 | 0.07 |
| GCST90274814 | Interleukin-5 levels | Weighted mode | 4 | 0.00 | 0.03 | 0.8786 | -0.05 | 0.06 |
| GCST90274815 | Interleukin-6 levels | MR Egger | 4 | -0.06 | 0.06 | 0.4199 | -0.16 | 0.05 |
| GCST90274815 | Interleukin-6 levels | Weighted median | 4 | -0.02 | 0.02 | 0.3907 | -0.05 | 0.02 |
| GCST90274815 | Interleukin-6 levels | IVW | 4 | -0.01 | 0.02 | 0.4807 | -0.04 | 0.02 |
| GCST90274815 | Interleukin-6 levels | Simple mode | 4 | -0.02 | 0.02 | 0.4127 | -0.07 | 0.03 |
| GCST90274815 | Interleukin-6 levels | Weighted mode | 4 | -0.02 | 0.02 | 0.4618 | -0.07 | 0.03 |
| GCST90274816 | Interleukin-7 levels | MR Egger | 4 | 0.09 | 0.06 | 0.2350 | -0.02 | 0.20 |
| GCST90274816 | Interleukin-7 levels | Weighted median | 4 | 0.04 | 0.02 | 0.0512 | 0.00 | 0.08 |
| GCST90274816 | Interleukin-7 levels | IVW | 4 | 0.03 | 0.02 | 0.0253 | 0.00 | 0.06 |
| GCST90274816 | Interleukin-7 levels | Simple mode | 4 | 0.05 | 0.03 | 0.1560 | 0.00 | 0.10 |
| GCST90274816 | Interleukin-7 levels | Weighted mode | 4 | 0.04 | 0.02 | 0.1856 | -0.01 | 0.09 |
| GCST90274817 | Interleukin-8 levels | MR Egger | 4 | 0.01 | 0.06 | 0.8713 | -0.10 | 0.12 |
| GCST90274817 | Interleukin-8 levels | Weighted median | 4 | -0.01 | 0.02 | 0.7832 | -0.04 | 0.03 |
| GCST90274817 | Interleukin-8 levels | IVW | 4 | 0.00 | 0.02 | 0.8292 | -0.03 | 0.03 |
| GCST90274817 | Interleukin-8 levels | Simple mode | 4 | -0.02 | 0.03 | 0.5330 | -0.07 | 0.03 |
| GCST90274817 | Interleukin-8 levels | Weighted mode | 4 | -0.02 | 0.03 | 0.5049 | -0.07 | 0.03 |
| GCST90274818 | Latency-associated peptide transforming growth factor beta 1 levels | MR Egger | 4 | -0.01 | 0.06 | 0.9406 | -0.13 | 0.12 |
| GCST90274818 | Latency-associated peptide transforming growth factor beta 1 levels | Weighted median | 4 | 0.01 | 0.02 | 0.5282 | -0.02 | 0.05 |
| GCST90274818 | Latency-associated peptide transforming growth factor beta 1 levels | IVW | 4 | 0.00 | 0.02 | 0.9211 | -0.03 | 0.03 |
| GCST90274818 | Latency-associated peptide transforming growth factor beta 1 levels | Simple mode | 4 | 0.01 | 0.03 | 0.6419 | -0.04 | 0.07 |
| GCST90274818 | Latency-associated peptide transforming growth factor beta 1 levels | Weighted mode | 4 | 0.01 | 0.02 | 0.5957 | -0.03 | 0.06 |
| GCST90274819 | Leukemia inhibitory factor levels | MR Egger | 4 | -0.07 | 0.06 | 0.3582 | -0.20 | 0.05 |
| GCST90274819 | Leukemia inhibitory factor levels | Weighted median | 4 | -0.02 | 0.02 | 0.4206 | -0.06 | 0.02 |
| GCST90274819 | Leukemia inhibitory factor levels | IVW | 4 | -0.01 | 0.02 | 0.6742 | -0.04 | 0.03 |
| GCST90274819 | Leukemia inhibitory factor levels | Simple mode | 4 | -0.03 | 0.03 | 0.4286 | -0.09 | 0.03 |
| GCST90274819 | Leukemia inhibitory factor levels | Weighted mode | 4 | -0.03 | 0.03 | 0.4257 | -0.09 | 0.03 |
| GCST90274820 | Leukemia inhibitory factor receptor levels | MR Egger | 4 | -0.10 | 0.11 | 0.4705 | -0.32 | 0.12 |
| GCST90274820 | Leukemia inhibitory factor receptor levels | Weighted median | 4 | 0.02 | 0.02 | 0.3918 | -0.02 | 0.06 |
| GCST90274820 | Leukemia inhibitory factor receptor levels | IVW | 4 | 0.01 | 0.03 | 0.8129 | -0.05 | 0.07 |
| GCST90274820 | Leukemia inhibitory factor receptor levels | Simple mode | 4 | 0.02 | 0.02 | 0.3900 | -0.02 | 0.07 |
| GCST90274820 | Leukemia inhibitory factor receptor levels | Weighted mode | 4 | 0.02 | 0.02 | 0.4219 | -0.02 | 0.06 |
| GCST90274821 | Monocyte chemoattractant protein-1 levels | MR Egger | 4 | -0.01 | 0.06 | 0.8965 | -0.13 | 0.11 |
| GCST90274821 | Monocyte chemoattractant protein-1 levels | Weighted median | 4 | -0.02 | 0.02 | 0.4548 | -0.06 | 0.02 |
| GCST90274821 | Monocyte chemoattractant protein-1 levels | IVW | 4 | -0.01 | 0.02 | 0.4761 | -0.04 | 0.02 |
| GCST90274821 | Monocyte chemoattractant protein-1 levels | Simple mode | 4 | -0.03 | 0.03 | 0.3909 | -0.09 | 0.03 |
| GCST90274821 | Monocyte chemoattractant protein-1 levels | Weighted mode | 4 | -0.03 | 0.03 | 0.3990 | -0.09 | 0.03 |
| GCST90274822 | Monocyte chemoattractant protein 2 levels | MR Egger | 4 | -0.05 | 0.08 | 0.5765 | -0.21 | 0.10 |
| GCST90274822 | Monocyte chemoattractant protein 2 levels | Weighted median | 4 | -0.01 | 0.02 | 0.5524 | -0.05 | 0.03 |
| GCST90274822 | Monocyte chemoattractant protein 2 levels | IVW | 4 | -0.01 | 0.02 | 0.7584 | -0.04 | 0.03 |
| GCST90274822 | Monocyte chemoattractant protein 2 levels | Simple mode | 4 | -0.01 | 0.03 | 0.7743 | -0.07 | 0.05 |
| GCST90274822 | Monocyte chemoattractant protein 2 levels | Weighted mode | 4 | -0.01 | 0.03 | 0.7965 | -0.06 | 0.04 |
| GCST90274823 | Monocyte chemoattractant protein-3 levels | MR Egger | 4 | -0.01 | 0.06 | 0.8813 | -0.13 | 0.11 |
| GCST90274823 | Monocyte chemoattractant protein-3 levels | Weighted median | 4 | 0.01 | 0.02 | 0.6840 | -0.03 | 0.05 |
| GCST90274823 | Monocyte chemoattractant protein-3 levels | IVW | 4 | 0.00 | 0.02 | 0.8597 | -0.03 | 0.04 |
| GCST90274823 | Monocyte chemoattractant protein-3 levels | Simple mode | 4 | 0.01 | 0.03 | 0.7593 | -0.04 | 0.06 |
| GCST90274823 | Monocyte chemoattractant protein-3 levels | Weighted mode | 4 | 0.01 | 0.02 | 0.7384 | -0.04 | 0.06 |
| GCST90274824 | Monocyte chemoattractant protein-4 levels | MR Egger | 4 | 0.01 | 0.06 | 0.8847 | -0.10 | 0.12 |
| GCST90274824 | Monocyte chemoattractant protein-4 levels | Weighted median | 4 | 0.00 | 0.02 | 0.8988 | -0.03 | 0.04 |
| GCST90274824 | Monocyte chemoattractant protein-4 levels | IVW | 4 | 0.00 | 0.02 | 0.8959 | -0.03 | 0.03 |
| GCST90274824 | Monocyte chemoattractant protein-4 levels | Simple mode | 4 | 0.01 | 0.02 | 0.7947 | -0.04 | 0.06 |
| GCST90274824 | Monocyte chemoattractant protein-4 levels | Weighted mode | 4 | 0.01 | 0.02 | 0.7445 | -0.03 | 0.05 |
| GCST90274825 | Macrophage inflammatory protein 1a levels | MR Egger | 4 | 0.06 | 0.06 | 0.3991 | -0.05 | 0.17 |
| GCST90274825 | Macrophage inflammatory protein 1a levels | Weighted median | 4 | 0.01 | 0.02 | 0.4912 | -0.03 | 0.05 |
| GCST90274825 | Macrophage inflammatory protein 1a levels | IVW | 4 | 0.01 | 0.02 | 0.5696 | -0.02 | 0.04 |
| GCST90274825 | Macrophage inflammatory protein 1a levels | Simple mode | 4 | 0.02 | 0.03 | 0.5597 | -0.04 | 0.08 |
| GCST90274825 | Macrophage inflammatory protein 1a levels | Weighted mode | 4 | 0.02 | 0.03 | 0.4896 | -0.03 | 0.07 |
| GCST90274826 | Matrix metalloproteinase-1 levels | MR Egger | 4 | 0.07 | 0.08 | 0.4343 | -0.08 | 0.22 |
| GCST90274826 | Matrix metalloproteinase-1 levels | Weighted median | 4 | 0.01 | 0.02 | 0.5291 | -0.03 | 0.05 |
| GCST90274826 | Matrix metalloproteinase-1 levels | IVW | 4 | 0.01 | 0.02 | 0.5035 | -0.03 | 0.05 |
| GCST90274826 | Matrix metalloproteinase-1 levels | Simple mode | 4 | 0.01 | 0.04 | 0.7211 | -0.06 | 0.08 |
| GCST90274826 | Matrix metalloproteinase-1 levels | Weighted mode | 4 | 0.02 | 0.04 | 0.7332 | -0.07 | 0.10 |
| GCST90274827 | Matrix metalloproteinase-10 levels | MR Egger | 4 | 0.01 | 0.06 | 0.8618 | -0.10 | 0.12 |
| GCST90274827 | Matrix metalloproteinase-10 levels | Weighted median | 4 | 0.00 | 0.02 | 0.8523 | -0.03 | 0.04 |
| GCST90274827 | Matrix metalloproteinase-10 levels | IVW | 4 | 0.01 | 0.02 | 0.6664 | -0.02 | 0.04 |
| GCST90274827 | Matrix metalloproteinase-10 levels | Simple mode | 4 | 0.00 | 0.03 | 0.8719 | -0.06 | 0.05 |
| GCST90274827 | Matrix metalloproteinase-10 levels | Weighted mode | 4 | 0.00 | 0.02 | 0.9187 | -0.04 | 0.04 |
| GCST90274828 | Neurturin levels | MR Egger | 4 | -0.02 | 0.06 | 0.7306 | -0.15 | 0.10 |
| GCST90274828 | Neurturin levels | Weighted median | 4 | 0.04 | 0.02 | 0.0493 | 0.00 | 0.09 |
| GCST90274828 | Neurturin levels | IVW | 4 | 0.04 | 0.02 | 0.0116 | 0.01 | 0.08 |
| GCST90274828 | Neurturin levels | Simple mode | 4 | 0.04 | 0.03 | 0.2738 | -0.02 | 0.11 |
| GCST90274828 | Neurturin levels | Weighted mode | 4 | 0.05 | 0.03 | 0.2132 | -0.01 | 0.11 |
| GCST90274829 | Neurotrophin-3 levels | MR Egger | 4 | -0.06 | 0.08 | 0.5257 | -0.20 | 0.09 |
| GCST90274829 | Neurotrophin-3 levels | Weighted median | 4 | 0.00 | 0.02 | 0.8935 | -0.04 | 0.04 |
| GCST90274829 | Neurotrophin-3 levels | IVW | 4 | 0.00 | 0.02 | 0.8233 | -0.04 | 0.03 |
| GCST90274829 | Neurotrophin-3 levels | Simple mode | 4 | 0.01 | 0.03 | 0.8238 | -0.06 | 0.08 |
| GCST90274829 | Neurotrophin-3 levels | Weighted mode | 4 | 0.02 | 0.03 | 0.6061 | -0.05 | 0.09 |
| GCST90274830 | Osteoprotegerin levels | MR Egger | 4 | -0.04 | 0.07 | 0.6705 | -0.17 | 0.10 |
| GCST90274830 | Osteoprotegerin levels | Weighted median | 4 | 0.02 | 0.02 | 0.2326 | -0.01 | 0.06 |
| GCST90274830 | Osteoprotegerin levels | IVW | 4 | 0.02 | 0.02 | 0.3152 | -0.02 | 0.05 |
| GCST90274830 | Osteoprotegerin levels | Simple mode | 4 | 0.03 | 0.02 | 0.3224 | -0.02 | 0.07 |
| GCST90274830 | Osteoprotegerin levels | Weighted mode | 4 | 0.02 | 0.02 | 0.3152 | -0.01 | 0.06 |
| GCST90274831 | Oncostatin-M levels | MR Egger | 4 | -0.02 | 0.06 | 0.7071 | -0.13 | 0.09 |
| GCST90274831 | Oncostatin-M levels | Weighted median | 4 | 0.01 | 0.02 | 0.4163 | -0.02 | 0.05 |
| GCST90274831 | Oncostatin-M levels | IVW | 4 | 0.00 | 0.02 | 0.8152 | -0.03 | 0.03 |
| GCST90274831 | Oncostatin-M levels | Simple mode | 4 | 0.01 | 0.02 | 0.5982 | -0.03 | 0.06 |
| GCST90274831 | Oncostatin-M levels | Weighted mode | 4 | 0.01 | 0.02 | 0.5290 | -0.03 | 0.06 |
| GCST90274832 | Programmed cell death 1 ligand 1 levels | MR Egger | 4 | 0.03 | 0.06 | 0.6887 | -0.08 | 0.13 |
| GCST90274832 | Programmed cell death 1 ligand 1 levels | Weighted median | 4 | 0.01 | 0.02 | 0.6395 | -0.03 | 0.05 |
| GCST90274832 | Programmed cell death 1 ligand 1 levels | IVW | 4 | 0.01 | 0.02 | 0.5603 | -0.02 | 0.04 |
| GCST90274832 | Programmed cell death 1 ligand 1 levels | Simple mode | 4 | 0.02 | 0.02 | 0.5286 | -0.03 | 0.06 |
| GCST90274832 | Programmed cell death 1 ligand 1 levels | Weighted mode | 4 | 0.01 | 0.02 | 0.6780 | -0.03 | 0.05 |
| GCST90274833 | Stem cell factor levels | MR Egger | 4 | 0.10 | 0.06 | 0.2051 | -0.01 | 0.21 |
| GCST90274833 | Stem cell factor levels | Weighted median | 4 | 0.00 | 0.02 | 0.8740 | -0.05 | 0.04 |
| GCST90274833 | Stem cell factor levels | IVW | 4 | 0.00 | 0.02 | 0.9236 | -0.04 | 0.04 |
| GCST90274833 | Stem cell factor levels | Simple mode | 4 | 0.04 | 0.03 | 0.3398 | -0.03 | 0.11 |
| GCST90274833 | Stem cell factor levels | Weighted mode | 4 | -0.01 | 0.02 | 0.5919 | -0.06 | 0.03 |
| GCST90274834 | SIR2-like protein 2 levels | MR Egger | 4 | -0.02 | 0.06 | 0.8063 | -0.12 | 0.09 |
| GCST90274834 | SIR2-like protein 2 levels | Weighted median | 4 | 0.02 | 0.02 | 0.2685 | -0.02 | 0.06 |
| GCST90274834 | SIR2-like protein 2 levels | IVW | 4 | 0.02 | 0.02 | 0.1851 | -0.01 | 0.05 |
| GCST90274834 | SIR2-like protein 2 levels | Simple mode | 4 | 0.01 | 0.03 | 0.6634 | -0.04 | 0.06 |
| GCST90274834 | SIR2-like protein 2 levels | Weighted mode | 4 | 0.02 | 0.02 | 0.3755 | -0.02 | 0.07 |
| GCST90274835 | Signaling lymphocytic activation molecule levels | MR Egger | 4 | 0.02 | 0.06 | 0.7612 | -0.09 | 0.13 |
| GCST90274835 | Signaling lymphocytic activation molecule levels | Weighted median | 4 | 0.00 | 0.02 | 0.8625 | -0.04 | 0.03 |
| GCST90274835 | Signaling lymphocytic activation molecule levels | IVW | 4 | 0.00 | 0.02 | 0.7981 | -0.03 | 0.03 |
| GCST90274835 | Signaling lymphocytic activation molecule levels | Simple mode | 4 | 0.00 | 0.02 | 0.9446 | -0.05 | 0.04 |
| GCST90274835 | Signaling lymphocytic activation molecule levels | Weighted mode | 4 | 0.00 | 0.02 | 0.9278 | -0.04 | 0.04 |
| GCST90274836 | Sulfotransferase 1A1 levels | MR Egger | 4 | -0.06 | 0.06 | 0.4288 | -0.18 | 0.06 |
| GCST90274836 | Sulfotransferase 1A1 levels | Weighted median | 4 | 0.01 | 0.02 | 0.7323 | -0.03 | 0.05 |
| GCST90274836 | Sulfotransferase 1A1 levels | IVW | 4 | 0.02 | 0.02 | 0.3711 | -0.02 | 0.05 |
| GCST90274836 | Sulfotransferase 1A1 levels | Simple mode | 4 | 0.00 | 0.03 | 0.9217 | -0.06 | 0.06 |
| GCST90274836 | Sulfotransferase 1A1 levels | Weighted mode | 4 | 0.00 | 0.03 | 0.9228 | -0.05 | 0.06 |
| GCST90274837 | STAM binding protein levels | MR Egger | 4 | -0.06 | 0.06 | 0.4199 | -0.16 | 0.05 |
| GCST90274837 | STAM binding protein levels | Weighted median | 4 | 0.02 | 0.02 | 0.3333 | -0.02 | 0.06 |
| GCST90274837 | STAM binding protein levels | IVW | 4 | 0.01 | 0.02 | 0.3553 | -0.02 | 0.04 |
| GCST90274837 | STAM binding protein levels | Simple mode | 4 | -0.01 | 0.03 | 0.6562 | -0.06 | 0.04 |
| GCST90274837 | STAM binding protein levels | Weighted mode | 4 | 0.03 | 0.02 | 0.3239 | -0.02 | 0.07 |
| GCST90274838 | Transforming growth factor-alpha levels | MR Egger | 4 | -0.02 | 0.06 | 0.7095 | -0.13 | 0.09 |
| GCST90274838 | Transforming growth factor-alpha levels | Weighted median | 4 | 0.01 | 0.02 | 0.6473 | -0.03 | 0.04 |
| GCST90274838 | Transforming growth factor-alpha levels | IVW | 4 | 0.01 | 0.02 | 0.6388 | -0.02 | 0.04 |
| GCST90274838 | Transforming growth factor-alpha levels | Simple mode | 4 | 0.01 | 0.02 | 0.6092 | -0.03 | 0.06 |
| GCST90274838 | Transforming growth factor-alpha levels | Weighted mode | 4 | 0.01 | 0.02 | 0.5898 | -0.03 | 0.06 |
| GCST90274839 | Tumor necrosis factor levels | MR Egger | 4 | 0.03 | 0.07 | 0.7183 | -0.11 | 0.17 |
| GCST90274839 | Tumor necrosis factor levels | Weighted median | 4 | -0.01 | 0.02 | 0.6658 | -0.05 | 0.03 |
| GCST90274839 | Tumor necrosis factor levels | IVW | 4 | -0.01 | 0.02 | 0.4417 | -0.05 | 0.02 |
| GCST90274839 | Tumor necrosis factor levels | Simple mode | 4 | 0.02 | 0.03 | 0.6512 | -0.05 | 0.08 |
| GCST90274839 | Tumor necrosis factor levels | Weighted mode | 4 | 0.00 | 0.03 | 0.9624 | -0.05 | 0.05 |
| GCST90274840 | TNF-beta levels | MR Egger | 4 | -0.02 | 0.06 | 0.7434 | -0.15 | 0.10 |
| GCST90274840 | TNF-beta levels | Weighted median | 4 | -0.01 | 0.02 | 0.7749 | -0.05 | 0.04 |
| GCST90274840 | TNF-beta levels | IVW | 4 | -0.01 | 0.02 | 0.6743 | -0.04 | 0.03 |
| GCST90274840 | TNF-beta levels | Simple mode | 4 | 0.00 | 0.03 | 0.9258 | -0.06 | 0.05 |
| GCST90274840 | TNF-beta levels | Weighted mode | 4 | 0.00 | 0.02 | 0.8674 | -0.05 | 0.04 |
| GCST90274841 | Tumor necrosis factor receptor superfamily member 9 levels | MR Egger | 4 | 0.02 | 0.06 | 0.7737 | -0.10 | 0.14 |
| GCST90274841 | Tumor necrosis factor receptor superfamily member 9 levels | Weighted median | 4 | -0.01 | 0.02 | 0.7607 | -0.05 | 0.03 |
| GCST90274841 | Tumor necrosis factor receptor superfamily member 9 levels | IVW | 4 | 0.00 | 0.02 | 0.8446 | -0.04 | 0.03 |
| GCST90274841 | Tumor necrosis factor receptor superfamily member 9 levels | Simple mode | 4 | -0.01 | 0.03 | 0.7890 | -0.06 | 0.04 |
| GCST90274841 | Tumor necrosis factor receptor superfamily member 9 levels | Weighted mode | 4 | -0.01 | 0.03 | 0.7782 | -0.06 | 0.04 |
| GCST90274842 | Tumor necrosis factor ligand superfamily member 14 levels | MR Egger | 4 | -0.05 | 0.06 | 0.5018 | -0.17 | 0.07 |
| GCST90274842 | Tumor necrosis factor ligand superfamily member 14 levels | Weighted median | 4 | 0.01 | 0.02 | 0.7549 | -0.04 | 0.05 |
| GCST90274842 | Tumor necrosis factor ligand superfamily member 14 levels | IVW | 4 | -0.01 | 0.02 | 0.7259 | -0.04 | 0.03 |
| GCST90274842 | Tumor necrosis factor ligand superfamily member 14 levels | Simple mode | 4 | 0.00 | 0.03 | 0.8968 | -0.05 | 0.06 |
| GCST90274842 | Tumor necrosis factor ligand superfamily member 14 levels | Weighted mode | 4 | 0.01 | 0.02 | 0.7455 | -0.04 | 0.06 |
| GCST90274843 | TNF-related apoptosis-inducing ligand levels | MR Egger | 4 | -0.07 | 0.06 | 0.3268 | -0.18 | 0.04 |
| GCST90274843 | TNF-related apoptosis-inducing ligand levels | Weighted median | 4 | 0.01 | 0.02 | 0.7615 | -0.03 | 0.05 |
| GCST90274843 | TNF-related apoptosis-inducing ligand levels | IVW | 4 | 0.00 | 0.02 | 0.8220 | -0.03 | 0.04 |
| GCST90274843 | TNF-related apoptosis-inducing ligand levels | Simple mode | 4 | -0.01 | 0.03 | 0.7250 | -0.07 | 0.05 |
| GCST90274843 | TNF-related apoptosis-inducing ligand levels | Weighted mode | 4 | 0.01 | 0.03 | 0.6590 | -0.04 | 0.06 |
| GCST90274844 | TNF-related activation-induced cytokine levels | MR Egger | 4 | 0.02 | 0.06 | 0.7145 | -0.09 | 0.13 |
| GCST90274844 | TNF-related activation-induced cytokine levels | Weighted median | 4 | -0.04 | 0.02 | 0.0336 | -0.08 | 0.00 |
| GCST90274844 | TNF-related activation-induced cytokine levels | IVW | 4 | -0.04 | 0.02 | 0.0035 | -0.07 | -0.01 |
| GCST90274844 | TNF-related activation-induced cytokine levels | Simple mode | 4 | -0.04 | 0.02 | 0.1668 | -0.09 | 0.00 |
| GCST90274844 | TNF-related activation-induced cytokine levels | Weighted mode | 4 | -0.04 | 0.03 | 0.1902 | -0.09 | 0.01 |
| GCST90274845 | Thymic stromal lymphopoietin levels | MR Egger | 4 | -0.04 | 0.06 | 0.5988 | -0.16 | 0.08 |
| GCST90274845 | Thymic stromal lymphopoietin levels | Weighted median | 4 | 0.00 | 0.02 | 0.8562 | -0.05 | 0.04 |
| GCST90274845 | Thymic stromal lymphopoietin levels | IVW | 4 | -0.01 | 0.02 | 0.3990 | -0.05 | 0.02 |
| GCST90274845 | Thymic stromal lymphopoietin levels | Simple mode | 4 | 0.00 | 0.03 | 0.8784 | -0.06 | 0.05 |
| GCST90274845 | Thymic stromal lymphopoietin levels | Weighted mode | 4 | 0.00 | 0.03 | 0.8844 | -0.05 | 0.05 |
| GCST90274846 | Tumor necrosis factor ligand superfamily member 12 levels | MR Egger | 4 | 0.01 | 0.06 | 0.8184 | -0.10 | 0.13 |
| GCST90274846 | Tumor necrosis factor ligand superfamily member 12 levels | Weighted median | 4 | 0.01 | 0.02 | 0.7475 | -0.03 | 0.04 |
| GCST90274846 | Tumor necrosis factor ligand superfamily member 12 levels | IVW | 4 | 0.00 | 0.02 | 0.9567 | -0.03 | 0.03 |
| GCST90274846 | Tumor necrosis factor ligand superfamily member 12 levels | Simple mode | 4 | -0.02 | 0.03 | 0.4738 | -0.08 | 0.03 |
| GCST90274846 | Tumor necrosis factor ligand superfamily member 12 levels | Weighted mode | 4 | 0.02 | 0.03 | 0.5705 | -0.04 | 0.07 |
| GCST90274847 | Urokinase-type plasminogen activator levels | MR Egger | 4 | 0.01 | 0.09 | 0.9259 | -0.16 | 0.18 |
| GCST90274847 | Urokinase-type plasminogen activator levels | Weighted median | 4 | -0.01 | 0.02 | 0.6529 | -0.05 | 0.03 |
| GCST90274847 | Urokinase-type plasminogen activator levels | IVW | 4 | -0.01 | 0.02 | 0.5956 | -0.05 | 0.03 |
| GCST90274847 | Urokinase-type plasminogen activator levels | Simple mode | 4 | -0.04 | 0.03 | 0.3046 | -0.11 | 0.02 |
| GCST90274847 | Urokinase-type plasminogen activator levels | Weighted mode | 4 | 0.00 | 0.03 | 0.8942 | -0.05 | 0.06 |
| GCST90274848 | Vascular endothelial growth factor A levels | MR Egger | 4 | -0.04 | 0.06 | 0.5069 | -0.15 | 0.06 |
| GCST90274848 | Vascular endothelial growth factor A levels | Weighted median | 4 | 0.00 | 0.02 | 0.8925 | -0.03 | 0.04 |
| GCST90274848 | Vascular endothelial growth factor A levels | IVW | 4 | 0.00 | 0.02 | 0.8860 | -0.03 | 0.03 |
| GCST90274848 | Vascular endothelial growth factor A levels | Simple mode | 4 | 0.00 | 0.02 | 0.9872 | -0.04 | 0.05 |
| GCST90274848 | Vascular endothelial growth factor A levels | Weighted mode | 4 | 0.01 | 0.02 | 0.8164 | -0.04 | 0.05 |

SNP, single-nucleotide polymorphism; β, regression coefficient; SE, standard error; OR, odds ratio; Lo_CI, odds ratio lower 95% confidence interval; UP_CI, odds ratio upper 95% confidence interval.
